# Meta-analysis of lipid-traits in Hispanics identifies novel loci, population-specific effects, and tissue-specific enrichment of eQTLs

**DOI:** 10.1038/srep19429

**Published:** 2016-01-19

**Authors:** Jennifer E. Below, Esteban J. Parra, Eric R. Gamazon, Jason Torres, S. Krithika, Sophie Candille, Yingchang Lu, Ani Manichakul, Jesus Peralta-Romero, Qing Duan, Yun Li, Andrew P. Morris, Omri Gottesman, Erwin Bottinger, Xin-Qun Wang, Kent D. Taylor, Y.-D. Ida Chen, Jerome I. Rotter, Stephen S. Rich, Ruth J. F. Loos, Hua Tang, Nancy J. Cox, Miguel Cruz, Craig L. Hanis, Adan Valladares-Salgado

**Affiliations:** 1 Division of epidemiology, Human Genetics & Environmental Sciences, University of Texas School of Public Health, Houston, Texas, USA; 2 Department of Anthropology, University of Toronto at Mississauga, Mississauga, Ontario, Canada; 3 Section of Genetic Medicine, Department of Medicine, University of Chicago, Illinois, USA; 4 Division of Genetic Medicine, Department of Medicine, Vanderbilt University, Nashville, TN; 5 Academic Medical Center, University of Amsterdam, Amsterdam, The Netherlands; 6 Department of Genetics, Stanford University School of Medicine, Stanford, California, USA; 7 The Charles Bronfman Institute for Personalized Medicine, The Icahn School of Medicine at Mount Sinai, New York, New York, USA; 8 The Genetics of Obesity and Related Metabolic Traits Program, The Icahn School of Medicine at Mount Sinai, New York, New York, USA; 9 Center for Public Health Genomics, University of Virginia, Charlottesville, Virginia, USA; 10 Unidad de Investigación Médica en Bioquímica, Hospital de Especialidades, Centro Médico Nacional Siglo XXI, IMSS, Mexico City, Mexico; 11 Department of Genetics and Department of Biostatistics, University of North Carolina at Chapel Hill, Chapel Hill, North Carolina, USA; 12 Department of Biostatistics, University of Liverpool, Liverpool, United Kingdom; 13 Department of Public Health Sciences, University of Virginia, Charlottesville, Virginia, USA; 14 Institute of Translational Genomics and Population Sciences, Los Angeles Biomedical Research Institute at Harbor/UCLA Medical Center, Torrance, California, USA

## Abstract

We performed genome-wide meta-analysis of lipid traits on three samples of Mexican and Mexican American ancestry comprising 4,383 individuals, and followed up significant and highly suggestive associations in three additional Hispanic samples comprising 7,876 individuals. Genome-wide significant signals were observed in or near *CELSR2*, *ZNF259/APOA5*, *KANK2/DOCK6* and *NCAN/MAU2* for total cholesterol, *LPL, ABCA1, ZNF259/APOA5*, *LIPC* and *CETP* for HDL cholesterol, *CELSR2, APOB* and *NCAN/MAU2* for LDL cholesterol, and *GCKR*, *TRIB1*, *ZNF259/APOA5* and NCAN/*MAU2* for triglycerides. Linkage disequilibrium and conditional analyses indicate that signals observed at *ABCA1* and *LIPC* for HDL cholesterol and *NCAN/MAU2* for triglycerides are independent of previously reported lead SNP associations. Analyses of lead SNPs from the European Global Lipids Genetics Consortium (GLGC) dataset in our Hispanic samples show remarkable concordance of direction of effects as well as strong correlation in effect sizes. A meta-analysis of the European GLGC and our Hispanic datasets identified five novel regions reaching genome-wide significance: two for total cholesterol (*FN1* and *SAMM50*), two for HDL cholesterol (*LOC100996634* and *COPB1*) and one for LDL cholesterol (*LINC00324/CTC1/PFAS*). The top meta-analysis signals were found to be enriched for SNPs associated with gene expression in a tissue-specific fashion, suggesting an enrichment of tissue-specific function in lipid-associated loci.

Cardiovascular disease (CVD) is one of the leading causes of death in the United States in every census category (non-Hispanic Whites, Blacks, Hispanic/Latino, Asian/Pacific Islanders and American Indian/Alaska Natives)^1^. Based on mortality data from 2010, it was estimated that more than 2,150 Americans die of CVD each day^1^. Similarly, in Mexico CVD is the leading cause of death, and the death rate attributed to CVD has increased from 53.4 per 100,000 in 1980 (4^th^ ranked cause of death) to 97.0 per 100,000 in 2010 (1^st^ ranked cause of death)^2^. Lipid concentrations are a major risk factor for CVD, and in the US there is a high prevalence of dyslipidemias^1^. In adults, the prevalence of total cholesterol concentrations ≥ 200 mg/dl was recently estimated as 43.4%, and the figures for LDL cholesterol ≥ 130 mg/dl, HDL cholesterol < 40 mg/dl and triglycerides ≥ 150 mg/dl were 31.1%, 21.8% and 27%, respectively^1^. In Mexico, a study based on the Mexican National Health and Nutrition Survey (ENSANUT 2006) indicated even higher prevalence at the same thresholds: 43.6% for total cholesterol concentrations, 46% for LDL cholesterol, 60.5% for HDL cholesterol, and 31.5% for triglycerides^3^. Given the relevance of dyslipidemia as a risk factor for CVD, and the important public health impact of this disease, it is not surprising that substantial efforts have been directed at deciphering the genetic architecture of lipid traits. In this respect, GWAS have proven to be very successful; and to date more than 150 loci have been associated with plasma lipid traits^4^,^5^. These efforts have led to the discovery of new regulatory pathways in lipid metabolism, opening new potential targets for therapeutic intervention, and demonstrated the relevance of the GWA approach from the clinical point of view^6^,^7^. Interestingly, several studies have shown that most of the SNPs identified in European populations have the same direction of association in other population groups^5^,^8^,^9^.

The remarkable advances described above have been primarily driven by studies in European populations, and the number of GWAS in other populations has been much more limited. To our knowledge, in the case of Hispanics, there have been only three GWAS published for lipid traits^10^,^11^,^12^. Here, we present the results of the largest effort to characterize the architecture of lipid traits in Hispanics, based on a combined sample of 12,259 individuals. In a first stage, we carried out a meta-analysis of lipid traits based on three samples of Mexican ancestry (from Mexico City and Starr County, Texas) comprising 4,383 individuals. In a second stage, we followed up the lead SNPs corresponding to the genome-wide and highly suggestive signals in three additional Hispanic samples (MESA, WHI and IPM BioMe Biobank) with 7,876 individuals.

## Materials and Methods

### Study participants

All study participants were originally ascertained for GWAS of type 2 diabetes, and so each cohort is enriched for type 2 diabetes patients. Descriptive statistics for each population for relevant traits are presented in Table 1.

Mexico City-sample 1: This sample comprises 967 individuals with type 2 diabetes and 343 controls. Detailed information about this sample is available in Parra *et al.*^13^ Informed consent was obtained from each participant, and the ethical research board (“Comité Local de Investigación”) of the Medical Center “Siglo XXI” approved the research, and the methods were carried out in accordance with the approved guidelines. The Ethics Review Office at the University of Toronto also approved this study.

Mexico City-sample 2: This sample comprises 898 individuals with type 2 diabetes and 889 controls. Informed consent was obtained from each participant, and the ethical research board (“Comité Local de Investigación”) of the Medical Center “Siglo XXI” approved the research, and the methods were carried out in accordance with the approved guidelines.

Starr County sample: This sample comprises 1,598 unrelated individuals with lipid measures and genetic data. We removed 312 individuals who were taking lipid medications and/or were missing one or more covariates for a final sample of 1,286 carried forward into analysis. Of these 536 are affected with type 2 diabetes and 740 are type 2 diabetes controls. All samples were collected in Starr County, Texas, and details of ascertainment can be found in Below *et al.*^14^. Informed consent was obtained from each participant, and the research was approved by the institutional review board of the University of Texas Health Science Center at Houston, and the methods were carried out in accordance with the approved guidelines.

### Measurement of plasma lipid concentrations

Mexico-City samples 1 and 2: Quantification of lipid traits (total cholesterol, HDL cholesterol, LDL cholesterol and triglycerides) was carried out with an IIab 300 Plus clinical chemistry analyser (Instrument Laboratory, Lexington, MA 02421-3125, USA), using standard protocols. The serum samples were obtained after fasting for at least 12 hours and the biochemical quantification took place within 3 hours of serum purification.

Starr County sample: EDTA plasma was obtained after overnight fast and placed on ice. Samples were spun and frozen within 30 minutes of collection and placed in a −80° freezer before the end of day. Serum HDL-C, triglycerides, and total cholesterol were measured using standard enzymatic methods. LDL-C was calculated using the Friedewald equation^15^, with missing values assigned for samples with triglyceride levels greater than 400 mg/dl.

### Genotyping

Mexico City-sample 1: This sample was genotyped with the Affymetrix Genome-wide Human SNP array 5.0 (Affymetrix, Santa Clara, CA) following standard protocols. Genotype calling was done with the Affymetrix PowerTools (APT) software package including the full sample set, and using two genotyping algorithms, the BRLMM-P and Birdseed algorithms. In order to minimize genotyping errors, the program PLINK v 1.06^16^ was used to merge the genotype results obtained with both algorithms, using the consensus call mode.

Mexico City-sample 2: This sample was genotyped with the Affymetrix Axiom LAT array (Affymetrix, Santa Clara, CA) following standard protocols. Genotype calling was done with the Affymetrix PowerTools (APT) software package, using the AxiomGT1/brlmm-p algorithm and the manufacturer recommended calling pipeline.

Starr County sample: This sample was genotyped according to manufacturer protocols using the Affymetrix Genome-Wide SNPArray 6.0 assay (Affymetrix, Santa Clara, CA, USA) at the Center for Inherited Disease Research. Case, impaired glucose tolerance, impaired fasting glucose, and control groups were randomized to plates. Genotypes were called for 1,890 samples passing quality control (QC) using both the Affymetrix supported Birdseed v2^167^ calling algorithm as well as the corrected robust linear model with maximum likelihood classification (CRLMM)^17^ and only calls in complete agreement between the two algorithms were used in analyses. An unrelated subset of 1,286 of these samples was included in the present analysis (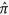 threshold of 0.28). Further details of the genotyping and quality control can be found in Below *et al.*^14^.

### Quality Control prior to imputation

Mexico City-sample 1: Prior to imputation, individuals were removed to clean the data of relationship structures at a threshold of 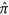 > 0.2. SNPs were removed from the initial list of autosomal markers (430,322 markers) based on the following criteria: 1) minor allele frequency < 1%, 2) Hardy-Weinberg p-values < 0.0001 in the control group and 3) missingness > 5% in the cases and the controls. After filtering, the final number of autosomal markers was 370,268. All the QC steps were carried out with the program PLINK^16^.

Mexico City-sample 2: Prior to imputation, individuals were removed to clean the data of relationship structures at a threshold of 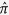 > 0.2. SNPs were removed from the initial list of autosomal markers (762,967 markers) based on the following criteria: 1) Markers classified as *CallRateBelowThreshold*, *OffTargetVariants* or *Other* by the program SNPolisher, using the human default thresholds (CR.cut ≥ 95, FLD.cut ≥ 3.6, HetSO.cut ≥ −0.1, HomRO2.cut ≥ 0.3, HomRO3.cut ≥ −0.9, nMinorAllele.cut ≥ 2), 2) minor allele frequency < 5%, 3) missingness < 0.01 and 4) Hardy-Weinberg p-values < 0.05 in the full sample. After filtering, the number of autosomal markers was 415,265. All the QC steps were carried out with the program PLINK^16^.

Starr County sample: Prior to imputation, individuals were removed to clean the data of relationship structures at a threshold of 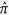 > 0.28 and ethnic outliers. SNPs were removed from the initial list of 868,155 genotyped autosomal markers based on the following criteria: 1) minor allele frequency < 1%, 2) Hardy-Weinberg p-values < 10^−4^ in the full sample 3) missingness > 10% in the full sample and 4) all ambiguous strand (AT/CG) SNPs. Individual-level missingness is < 5% in all samples. 603,042 scaffold SNPs were carried forward into imputation.

### Imputation

Mexico City-sample 1: Imputation was carried out with the program IMPUTE v2^17^,^18^ (see Web Resources) using the combined 1000 Genomes project samples as the reference sample (1000 Genomes Phase I integrated variant set, NCBI build b37, release date March 2012, no singletons). Imputations followed a two-step strategy: 1) pre-phasing using the program SHAPEIT^19^ (see Web Resources) and 2) imputation from the reference panel into the estimated haplotypes with IMPUTE v2^17^,^18^,^20^.

Mexico City-sample 2: Imputation was carried out with the program IMPUTE v2, using the combined 1000 Genomes project samples as the reference sample (1000 Genomes Phase I integrated haplotypes, NCBI build b37, release date December 2013, no singletons). Imputations followed a single-step strategy (simultaneous phasing and imputation)^17^.

Starr County sample: Imputation was carried out with the program IMPUTE v2 using the combined (all ancestries) 1000 Genomes Phase 1 integrated set March 2012 release. Imputations followed a two-step strategy similar to that applied to the Mexico City 1 sample.

### Population stratification

We used the program EIGENSOFT^21^ (see Web Resources) to perform a principal components analysis and evaluate population stratification after pruning markers in high LD and removing regions showing high LD or genomic complexity. The principal components analyses were carried out in two ways: 1) including only the individuals of the study samples (e.g. Mexico City and Starr County), in order to characterize the extent of population stratification, and 2) including the study samples and other reference populations, in order to gain a better perspective about population history.

### Association tests

Mexico City-sample 1: For this sample, detailed information is available about lipid-lowering medications for each participant (e.g. statins or fibrates). Prior to the statistical analysis, plasma lipid concentrations were adjusted based on the effect of the medication, as suggested by Wu *et al.*^22^. For participants taking statins, lipid concentrations were modified as follows: Total cholesterol (+52.1 mg/dl), LDL cholesterol (+49.9 mg/dl), HDL cholesterol (−2.3 mg/dl) and triglycerides (+18.4 mg/dl). For participants taking fibrates, lipid concentrations were modified as follows: Total cholesterol (+46.1 mg/dl), LDL cholesterol (+40.1 mg/dl), HDL cholesterol (−5.9 mg/dl) and triglycerides (+57.1 mg/dl). After adjusting lipid concentrations based on information about lipid treatment, we ran a linear regression using the lipid concentrations as dependent variable and sex, age, age^2^, BMI, first PC values and diabetes status as covariates. The residuals were then inverse normal transformed using the Blom method. Associations of the genetic markers with the transformed residuals were tested with the program SNPTEST v2^18^ (see Web Resources) using the frequentist association tests implemented in the program, based on an additive model. In order to control for genotype uncertainty, we used the missing data likelihood score test (the *score* method).

Mexico City-sample 2: For this sample, association tests were carried out using the same strategies described for Mexico City-sample 1 above.

Starr County sample: For this sample, individuals identified as taking lipid-lowering medications were removed from further analysis. This resulted in exclusion of 308 individuals, and analysis of 1,286 subjects not taking lipid-lowering medications. Association tests were carried out using the same strategies described for Mexico City-sample 1 above.

### Meta-analysis

The program META (see Web Resources) was used to carry out an inverse variance fixed-effects meta-analysis. We used the option –lambda to implement genomic control methods for the analysis of triglycerides, the only trait to show evidence of inflation (lambda values used for Mexico City sample 1, Mexico City sample 2, and Starr County were 1, 1, and 1.04, respectively). Because this correction had insignificant effects on top signals, all p-values reported are from uncorrected meta-analyses for consistency. All other lambdas were between 1.01 and 0.97.

### Identification of markers to follow-up and replication in independent Hispanic samples

Based on the results of the meta-analysis, we identified a list of regions to follow up, using the following criteria: 1) genome-wide significant (p < 5 × 10^−8^) or highly suggestive (p < 10^−5^) values in the meta-analysis, 2) valid p-values in the three samples and 3) good imputation quality (info scores > 0.7 in each individual study). The most significant SNP in each region was selected for follow-up in three independent Hispanic samples. The first sample comprises 3,587 Hispanic women from the Women’s Health Initiative SNP Health Association Resource (WHI-SHARe). The WHI is a U.S.-wide study focusing on common health issues in postmenopausal women. A total of 161,808 postmenopausal women aged 50–79 years old were recruited, including 12,151 self-identified African Americans and 5,469 self-identified Hispanics. A cohort of 3,642 self-identified Hispanic participants from WHI, who had consented to genetic research, were genotyped on the Affymetrix 6.0 array. After QC, the Hispanic sample available for replication of the lipid signals included 3,587 individuals. Imputation was carried out in two steps: 1) Phasing using MaCH1 and 2) Genotype imputation using minimac. For imputation, the 1000 Genomes Phase I Integrated Release Version 3 Haplotypes (2010–11 data freeze, 2012-03-14 haplotypes) were used as reference samples. Based on the LDL-lowering effects of statins, the pretreatment LDL concentrations for individuals on lipid-lowering medication were estimated by dividing treated LDL values by 0.75. The second sample comprises 2,127 Hispanic individuals from the Multi-Ethnic Study of Atherosclerosis (MESA) project. MESA is a study of the characteristics of subclinical cardiovascular disease (disease detected non-invasively before it has produced clinical signs and symptoms) and the risk factors that predict progression to clinically overt cardiovascular disease or progression of the subclinical disease. MESA researchers study a diverse, population-based sample of 6,814 asymptomatic men and women aged 45–84. Thirty-eight percent of the recruited participants are White, 28% African-American, 22% Hispanic, and 12% Asian, predominantly of Chinese descent. 2,128 additional individuals from 594 families were recruited through MESA Family. Participants were recruited from six field centers across the United States: Wake Forest University, Columbia University, Johns Hopkins University, University of Minnesota, Northwestern University and University of California - Los Angeles. All participants were genotyped using the Affymetrix Human SNP array 6.0, with 897,981 SNPs passing study specific quality control (QC). IMPUTE version 2.2.2 was used to perform imputation using the cosmopolitan 1,000 Genomes Phase 1 v3 March 2012 reference set. For the current analyses, we included 2,127 Hispanic individuals from the combined MESA and MESA Family samples^23^. Plasma lipid concentrations were adjusted for medication use as suggested by Wu *et al.*^24^. The third sample includes 2,162 individuals of Hispanic ancestry from the Charles Bronfman Institute for Personalized Medicine (IPM) BioMe Biobank. The BioMe is an Electronic Medical Record (EMR)-linked biobank that integrates research data and clinical care information for consented patients at The Mount Sinai Medical Center, which serves diverse local communities of upper Manhattan with broad health disparities. BioMe has enrolled over 31,500 participants between September 2007 and October 2014: 25% of African American ancestry (AA), 36% of Hispanic Latino ancestry (HL), 30% of white European ancestry (EA), and 9% of other ancestry. The IPM BioMe Biobank samples were genotyped using the Illumina HumanOmniExpress + v1.1 BeadChip and imputation was carried out with the program IMPUTE using the 1000 Genomes project Phase 1 integrated variant release v3, March 2012. Related individuals were removed from the sample using an IBD threshold of 0.185. The final Hispanic sample comprised 2,162 individuals. The lipid values were extracted from the outpatient lab test results from the 30-day period around enrollment (enrollment date + /− 15 days, value closest to enrollment date taken). LDL-cholesterol was calculated using the Friedewald equation; individuals with triglyceride levels greater than 400 mg/dl were assigned missing values. Information on use of lipid lowering medication (including information on use of statins and fibrate classes) prescribed at enrollment date + 15 days was also extracted. Plasma lipid concentrations were adjusted for medication use as suggested by Wu *et al.*^24^.

The statistical analyses in the replication samples were carried out following the same strategy described for the two Mexico City samples and the Starr County samples. For all lipid traits, the residuals of a linear regression including relevant covariates were transformed using the inverse normal transformation and these transformed residuals were used as dependent variables in association tests based on an additive model. In MESA, analysis was performed using a linear mixed effects model to account for family structure in the combined MESA and MESA family samples.

### Conditional analysis

In order to evaluate if the signals observed in the Hispanic samples are independent of the signals described in the Global Lipids Genetics Consortium (GLGC) European samples, we carried out conditional analyses in the Hispanic samples. Briefly, for the conditional analyses, we tested the association of the GLGC lead SNPs with the relevant lipid traits in each of our three Hispanic samples, using the Hispanic lead SNPs as covariates. We then used the program META to carry out an inverse variance fixed-effects meta-analysis and compared the p-values and effect sizes of the European lead SNP with and without conditioning on our lead SNP.

### Gene-based and pathway association tests

We conducted gene-based and pathway association tests using the program KGG3 (see Web Resources). For the gene-based association tests, we used the GATES approach^24^, using a window of 10Kb for the extended gene region length. This test only requires the p-values of SNPs within a gene, and does not use individual genotype data. The gene-based p values are obtained combining the p-values of the individual SNPs within the gene, using a modification of the Simes method that takes into account the pair-wise correlation coefficients for the SNPs, which are primarily determined by the LD patterns. In order to define the LD patterns relevant to our samples, we used the MXL (Mexican Americans from Los Angeles) 1000 Genomes sample. For the pathway association tests we used the HYST^25^ test implemented in the KGG3 program. We report only pathways that are significant using the HYST test and also a hypergeometric test enrolling genes with a p-value of 0.001. We also performed a pathway analysis using an alternative program, GSA-SNP^26^, using the PAGE (parametric analysis of gene set enrichment) method^27^. When using GSA-SNP, we selected the SNP with the 2^nd^ lowest p-value to define the gene p-values, and used a padding window of 10 kb. The program was run using both Gene Ontology (GO) and KEGG gene sets. We report only gene sets with False Discovery Rates (FDR) < 0.01.

### eQTL analyses

We investigated the extent to which expression-associated SNPs (eSNPs) identified in LCLs^28^, liver^29^, adipose, and muscle^30^ can be utilized to detect lipids-associated loci. We examined the (meta-analysis) association of the eSNPs identified in each tissue with each lipid trait and generated a quantile- quantile (Q-Q) plot from the distribution of p-values. We defined the false discovery rate (FDR) as follows^31^,^32^:


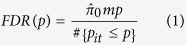


where *m* is the number of eSNPs tested, 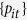 is the set of their p-values with a lipids trait *t* indexed by the eSNP *i*, and 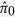 is the estimated proportion of null SNPs:


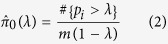


for a given tuning parameter 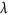 (selected, as in Storey *et al.*^33^, as 0.5). The eSNPs for each tissue that meet the Bonferroni corrected threshold as well as threshold for FDR < 0.05 for association with a lipid trait were identified.

### Web Resources

- The program IMPUTE2 and its documentation can be downloaded at https://mathgen.stats.ox.ac.uk/impute/impute_v2.html
- The program SHAPEIT and its documentation can be downloaded at https://mathgen.stats.ox.ac.uk/genetics_software/shapeit/shapeit.html
- The program EIGENSOFT and its documentation can be downloaded at http://genetics.med.harvard.edu/reich/Reich_Lab/Software.html
- The program SNPTEST and its documentation can be downloaded at https://mathgen.stats.ox.ac.uk/genetics_software/snptest/snptest.html
- The program META and its documentation can be downloaded at http://www.stats.ox.ac.uk/~jsliu/meta.html
- KGG:A systematic biological Knowledge-based mining system for Genome-wide Genetic studies can be accessed at http://statgenpro.psychiatry.hku.hk/limx/kgg/
- The study description for STAMPEED: Cardiovascular Health Study (CHS) GWAS to identify genetic variants associated with aging and CVD risk factors and events can be found at http://www.ncbi.nlm.nih.gov/projects/gap/cgi-bin/study.cgi ? study_id = phs000226.v3.p1
- The study description for the NHLBI Family Heart Study (FamHS-Visit1 and FamHS-Visit2) can be found at http://www.ncbi.nlm.nih.gov/projects/gap/cgi-bin/study.cgi ? study_id = phs000221.v1.p1
- Information about the COPB1 gene can be found at http://www.uniprot.org/uniprot/P53618

## Results

### Characterization of admixture in the samples

We carried out a principal components analysis of the Mexico City and Starr County samples using genome-wide data in order to identify population stratification and to characterize population history. The samples were plotted jointly with samples from other relevant populations. The plots for the three samples are depicted in Figure S1. The individuals from Mexico City are widely distributed between the clusters corresponding to the European and Native American samples (particularly, the Nahua Native American group from Central Mexico), clearly indicating that these two samples are primarily the result of admixture between European and Native American populations, with little evidence of African admixture. Native American reference samples were not plotted in the principal components representation of the Starr County samples. However, similarly to the individuals from Mexico City, the distribution of the samples from Starr County in the plots clearly indicate that there is a broad range of European/Native American ancestral contributions, with very few individuals showing evidence of African ancestry.

### Meta-analysis of lipid traits in samples of Mexican ancestry

We carried out a meta-analysis of lipid traits based on three genome-wide association studies including more than 4,700 individuals of Mexican ancestry (two samples from Mexico City and one sample from Starr County, Texas). Figures S2 and S3 show the Manhattan plots and QQ plots corresponding to the meta-analysis for each lipid trait. The genome-wide significant signals (p < 5 × 10^−8^) identified in this analysis are depicted in Table 2. The signals correspond to the following regions: *CELSR2* (lead SNP rs7528419, p = 7.9 × 10^−12^) and *NCAN* (lead SNP rs2238675, p = 1.4 × 10^−8^) for total cholesterol, *CETP* (lead SNP rs7499892, p = 4.2 × 10^−17^), *ABCA1* (lead SNP rs2472386, p = 5.1 × 10^−10^) and *ZNF259/APOA5* (lead SNP rs2367970, p = 5.8 × 10^−9^) for HDL cholesterol, *CELSR2* (lead SNP rs660240, p = 1.2 × 10^−12^) for LDL cholesterol, and *ZNF259/APOA5* (lead SNP rs964184, p = 5.3 × 10^−37^) and *MAU2* (lead SNP rs8102280, p = 9.9 × 10^−10^), which is located close to the *NCAN* gene on chromosome 19, for triglycerides. We also identified many suggestive signals (p < 10^−5^), including signals in well-known regions such as *TRIB1*, *ZNF259/APOA5* and *KANK2/DOCK6* for total cholesterol, *LPL* and *LIPC* for HDL cholesterol, *APOB* and *NCAN* for LDL cholesterol and *GCKR* and *TRIB1* for triglycerides. We prioritized a list of SNPs to follow up in independent Hispanic samples, based on the imputation scores and the presence of valid p-values in the three samples included in the meta-analysis. Supplemental File 1 shows the list of markers that were followed up, including information about the effect allele and estimated effect size, p-value of the meta-analysis and p-values of the individual studies (Starr County, Mexico City samples 1 and 2). For several regions, we observed different lead SNPs for different lipid traits: *CELSR* (Total cholesterol and LDL), *TRIB1* (Total cholesterol and triglycerides), *ZNF259* (Total cholesterol, HDL and LDL), and *NCAN/MAU2* (Total cholesterol, LDL and triglycerides). LocusZoom^33^ plots for top regions are presented in Supplemental Figures S4-S7. We carried out conditional analyses to evaluate whether these signals are independent. Supplemental File 2 depicts the results of these analyses.

### Follow-up of genome-wide significant and suggestive signals in independent Hispanic samples

Table 3 depicts the results of the follow-up for genome-wide significant SNPs, including the p-values, effect sizes and standard deviations observed in the discovery sample, the three replication cohorts (WHI, MESA, IPM BioMe Biobank), and the meta-analysis based on 12,259 samples. The table also indicates the p-values corresponding to Cochran’s Q test evaluating the heterogeneity of effect sizes. The results for the complete set of markers that were followed-up in the three replication cohorts are presented in Supplemental File 3. After combining the three replication samples, genome-wide significant signals were observed in *CELSR2*, *ZNF259/APOA5*, *KANK2/DOCK6* and *NCAN* for total cholesterol, *LPL, ABCA1, ZNF259/APOA5*, *LIPC* and *CETP* for HDL cholesterol, *CELSR2, APOB* and *NCAN* for LDL cholesterol, and *GCKR*, *TRIB1*, *ZNF259/APOA5* and *MAU2* for triglycerides. Another region between the genes *SCAMP1/AP3B1* was borderline genome-wide significant for triglycerides (rs4588572, p = 7.4 × 10^−8^).

### Comparing direction of effects and effect sizes of lead SNPs in European and Hispanic samples

We carried out a diverse set of analyses to evaluate the concordance of the signals observed in European and Hispanic samples. First, we investigated concordance in the direction of effects for markers showing genome-wide significance in the GLGC European samples^4^. In order to do this, we identified markers showing genome-wide significance for each trait in the GLGC dataset, and for the lead SNPs in each region, we retrieved information about effect sizes and direction of effects in our Hispanic dataset. The majority of the European lead SNPs have the same direction of effect in the Hispanic samples. For total cholesterol, 85.1% of the European lead SNPs (63 out of 74) have concordant effects (binomial p-value 2 × 10^−10^). For HDL cholesterol, LDL cholesterol and triglycerides, the percentage of markers showing concordant effects are 72.4% (55 out of 76, p-value = 4 × 10^−5^), 81.8% (54 out of 66, p-value = 7 × 10^−8^), and 80.4% (45 out of 56, p-value = 2 × 10^−6^), respectively. When we restrict the comparisons to the European lead SNPs that are also nominally significant in the Hispanic sample (p < 0.05), the concordance is almost perfect: 100% for total cholesterol (19/19, p-value = 2 × 10^−6^), 94.4% for HDL cholesterol (17/18, p-value = 7 × 10^−5^), 100% for LDL cholesterol (15/15, p-value = 3 × 10^−5^), and 100% for triglycerides (11/11, p-value = 5 × 10^−4^) (Supplemental File 4). The analysis described above provides interesting information about the concordance in direction of effects, but it does not explore the extent to which there may be heterogeneity in effect sizes. When we carried-out a meta-analysis of the European and Hispanic samples using the inverse variance method (based on fixed-effect models), only 5 out of 74 markers (6.8%) for total cholesterol, 9 out of 76 markers (11.8%) for HDL cholesterol, 8 out of 66 markers (12.1%) for LDL cholesterol, and 3 out of 56 markers (5.4%) for triglycerides showed evidence of heterogeneity in allele effects. We carried out a similar concordance analysis based on the genome-wide and highly suggestive signals that we selected for follow-up in our Hispanic sample. In agreement with the results for the GLGC European sample, for the list of followed-up markers we see a concordance higher than 50%, except for LDL cholesterol. For total cholesterol, 60% of the Hispanic lead SNPs present in the GLGC dataset had concordant effects (6 out of 10 SNPs, p-value = 0.2051). For HDL cholesterol, LDL cholesterol and triglycerides the concordance was 66.6% (6 out 9 SNPs, p-value = 0.1641), 50% (4 out of 8 SNPs, p-value = 0.2734) and 72.7% (8 out of 11 markers, p-value = 0.0807). There is perfect concordance when considering only markers that are nominally significant in the GLGC dataset: 100% for total cholesterol (5 out of 5, p-value = 0.0312), 100% for HDL cholesterol (5 out of 5, p-value = 0.0312), 100% for LDL cholesterol (3 out of 3, p-value = 0.125) and 100% for triglycerides (4 out of 4, p-value = 0.0625).

### LD patterns and conditional analyses in European and Hispanic signals

For the signals that we identified in the Hispanic sample (both genome-wide significant and highly suggestive) and that have been previously reported in European populations, we explored in more detail the pattern of linkage disequilibrium (LD) between the lead SNP in Europeans (GLGC lead SNP) and the lead SNP in our Hispanic meta-analysis. Low LD between the European and Hispanic lead SNPs may indicate that the signals are independent or that the pattern of LD between the causal SNP and the surrounding markers is different in European and Hispanic populations. In the latter scenario, combining the data from both populations could provide higher fine mapping resolution. Supplemental File 5 shows a detailed analysis of the LD patterns observed between the lead SNPs in the European and Hispanic samples for 10 relevant regions (*CELSR2, GCKR, APOB, ABCA1, LPL, TRIB1, ZNF259, LIPC, CETP* and *NCAN/MAU2*). The LD patterns are reported as the r^2^ values observed in the 1000 Genomes CEU (obtained using the program SNAP) and MLX samples (Mexican Americans from LA, obtained using the program PLINK). We also estimated r^2^ values in one of our Mexico City samples, and observed very similar values to those of the MLX samples (data not shown). The table also shows the p-values of the lead European SNP in the Hispanic meta-analysis, and the lead Hispanic SNP in the European meta-analysis. Additionally, we carried out conditional analyses in the Hispanic samples, in which we conditioned on our lead SNP in the statistical analyses, and compared the p-values and effect sizes of the European lead SNP with and without conditioning on our lead SNP. These analyses are presented in Table 4.

### Meta-analysis of European and Hispanic datasets

The European (GLGC dataset) and Hispanic studies used a similar statistical approach, based on an analysis of association of SNPs to inverse normal transformed traits. We carried out an inverse variance fixed-effects meta-analysis of the European^4^ and Hispanic data. We identified five regions that reached genome-wide significance in the meta-analysis and are nominally significant (but not genome-wide significant) in both the European and Hispanic samples. Table 5 describes these regions in more detail. For total cholesterol, the genome-wide significant regions are located within the gene *FN1* on chromosome 2 (lead SNP rs1250229, p-meta = 1.04 × 10^−8^) and *SAMM50* on chromosome 22 (lead SNP rs2235776, p-meta = 2.85 × 10^−8^). For HDL cholesterol, there are two genome-wide significant regions, corresponding to *LOC100996634* on chromosome 6 (lead SNP rs884366, p-meta = 1.40 × 10^−8^) and *COPB1* on chromosome 11 (lead SNP rs7121538, p-meta = 2.11 × 10^−8^). For LDL cholesterol a genome-wide significant signal was observed in *LINC00324/CTC1/PFAS* (lead SNP rs4791641, p-meta = 1.13 × 10^−8^).

### Analysis of signals reported in previous GWA studies in Mexicans

There have been two recent GWA studies for lipid traits in Mexican samples^11^,^12^. In the first study, genome-wide significant signals were identified at *ZNF259, GCKR, and LPL* for triglycerides, and *CETP*, *LIPC*, *LOC55908* and *ABCA1* for HDL^11^). Additionally, this study identified a novel locus for triglycerides located within the *TMEM241* gene at chr18q11 (lead snp rs9949617, p = 2.4 × 10^−8^)^11^. The second study used a different analytical approach, in which only variants showing differences in frequency between European and Native American populations were included in the GWAS^12^. This GWAS identified genome-wide significant signals at the *LPL*, *ZNF259* and *SIK3* genes for triglycerides, *UGT8*, *SIK3*, *RORA* and *CETP* for HDL cholesterol, and *CELSR* and *CETP* for total cholesterol. Of these, *UGT8* and *RORA* are novel regions and the signal identified on *SIK3*, which is located close to the *APOA5/ZNF259* region on chromosome 11, corresponds to an intronic SNP that is common in Mexicans but not observed in a Finnish sample^12^. Supplemental File 6 reports the p-values of those loci in our meta-analysis. We could not replicate the signals reported for *TMEM241*, *UGT8*, *RORA* and *SIK3* in our meta-analysis.

### Gene-based and Pathway analyses

The results of the gene-based association analyses for all traits using the program KGG3 are shown in Supplemental File 7. In the gene-based tests, we observed genome-wide significance for *CELSR2*, *NCAN*, *KIAA1462*, *LOC102467074*, *RPL34* and the *APOA5/BUD13/ZNF259* region for total cholesterol, *CETP*, *ABCA1*, *LIPC*, *LPL*, *KIF3C* and the *APOA5/BUD13/ZNF259* region for HDL cholesterol, *CELSR2* for LDL cholesterol and *NCAN* and *APOA5/BUD13/ZNF259* region for triglycerides (Supplemental File 7). Supplemental Files 7 and 8 show the results of the pathway-based association analyses using the programs KGG3 (Supplemental File 7) and GSA-SNP (Supplemental File 8). Although both programs use different strategies to define gene-based p-values and rely on different databases to define the pathways, they identified numerous common pathways relevant to lipid metabolism (see Supplemental Files 7 and 8 for detailed information).

### eQTL analyses

We carried out genome-wide association analyses of eSNPs reported for LCL^28^, human liver^29^, adipose and muscle^30^ and lipid traits. The genome-wide signals observed using an FDR of 5% are reported in Supplemental File 9. For each tissue and lipids trait, we tested whether the eSNPs identified in the tissue showed an excess of low p-values for association with the trait beyond what is expected under the null hypothesis. We generated a Q-Q plot from the distribution of meta-analysis p-values for the eSNPs identified in each tissue, and quantified the improvement in false discovery rate (see Methods) to detect trait-associated SNPs using the tissue-specific eSNPs. Among the eSNPs identified in adipose, muscle and liver, we observed a consistent, though tissue-dependent, enrichment for associations with all lipids traits (Figure S8). In contrast, we observed no such enrichment for associations with total cholesterol and LDL cholesterol among the LCL eSNPs (Figure S8). The leftward shifts of the observed distribution from the diagonal (“null”) line corresponding to FDR < 0.25, FDR < 0.10, and FDR < 0.05 provide a quantification of enrichment^34^. Taken together, these results suggest that tissue-specific regulatory variation can be used to detect lipid-associated SNPs with improved power.

## Discussion

We carried out a meta-analysis of three GWA studies for lipid traits including 4,383 individuals of Mexican/Mexican American ancestry. We identified genome-wide significant signals in the following regions: *CELSR2* and *NCAN* for total cholesterol, *CETP*, *ABCA1* and *ZNF259/APOA5* for HDL cholesterol, *CELSR2* for LDL cholesterol, and *ZNF259/APOA5* and *MAU2*, which is located close to the *NCAN* gene on chromosome 19, for triglycerides (Table 2). For *CELSR2*, *TRIB1*, *ZNF259/APOA5* and *NCAN/MAU2* we observed genome-wide or suggestive signals for more than one lipid trait and the lead SNPs were in some cases different. Our conditional analyses indicate that in these regions we are capturing the same signal, instead of independent loci (Supplemental File 2). We followed-up all of our genome-wide significant lead SNPs, as well as lead SNPs in other regions showing suggestive association in our study (p < 10 − 5), in three independent Hispanic cohorts for which lipid data were available (WHI, MESA and IPM BioMe Biobank, for details see Materials and Methods section). We used the same analytical strategy for all the samples, testing the association of directly genotyped and imputed genetic markers with inverse normal transformed residuals obtained using a common set of covariates, assuming an additive genetic model. Combining the original and the replication samples (12,259 samples), we were able to confirm the genome-wide significant signals identified in our original analysis. Additionally, other suggestive regions reached genome-wide significance in this expanded analysis, including *ZNF259/APOA5* and *KANK2/DOCK6* for total cholesterol, *LPL* and *LIPC* for HDL cholesterol, *APOB* and *NCAN* for LDL cholesterol, and *GCKR* and *TRIB1* for triglycerides (Table 3). All genome-wide significant regions identified in the discovery sample (4,383 individuals) as well as regions reaching genome-wide significance in the larger Hispanic sample including the replication cohorts (12,259 individuals) have been previously associated with lipid traits. Another region between the genes *SCAMP1/AP3B1* showed borderline genome-wide significance for triglycerides (rs4588572, p = 7.4 × 10^−8^) in the meta-analysis including the replication cohorts. For this region, all the cohorts studied show concordant effects. Interestingly, this intergenic region has been associated with VLDL lipoproteins, insulin resistance and coronary disease in previous studies^35^,^36^ (STAMPEED, see Web Resources). However, the SNPs that have been associated with the aforementioned traits are located more than 300 Kbp from our lead SNP, and are not in LD with our lead SNP in Europeans or in Mexican-Americans (data not shown). Our lead SNP in this region, rs4588572, is present in the European GLGC dataset, but is not nominally associated with triglyceride levels (p = 0.1037).

The availability of imputed data obtained using dense 1000 Genomes reference panels made it possible to explore in detail the concordance between the signals observed in studies carried out in European and Hispanic samples. Our analyses of the lead SNPs of the European GLGC dataset in our Hispanic samples indicate that there is a remarkable concordance of effects in both populations for all lipid traits (concordance rates range between 72.4% and 85.1%, p-values range between 4 × 10^−5^ to 2 × 10^−10^), and the concordance is near perfect when restricting the comparisons to markers that are nominally significant in the Hispanic sample. Similar analyses for the markers that were selected for follow-up in the Hispanic meta-analysis also demonstrate an excess of concordance of effects for all traits, except LDL cholesterol. Many of the lead SNPs identified in our Hispanic sample are not present in the GLGC dataset, because it was not imputed using the latest 1000 Genomes reference panels. As a result, the sample sizes for these analyses were small (8−11 SNPs), and in most cases the results are not significant. We also observed very little evidence of heterogeneity in effect sizes between the European and Hispanic samples. Only a small fraction of the lead GLGC SNPs analyzed (between 5.4% and 12.1%) showed significant differences in effect sizes between the European and Hispanic datasets and there is a strong correlation of effect sizes across the two samples (Figure S9). Our results are in agreement with recent studies reporting that the majority of the common lead snps identified in European populations (in which most of the GWA studies have taken place) are also relevant for other population groups. For example, Coram *et al.*^10^ reported that loci showing genome-wide significance or suggestive evidence of association in Europeans had a strong enrichment in small p-values in the Women Health Initiative (WHI) African American and Hispanic cohorts. These authors also found strong correlation of genetic effects per allele in the WHI African American and Hispanic samples for loci with p ≤ 10^−5^ in Europeans. Teslovich *et al.*^5^ found that most of the lipid loci discovered in a large European meta-analysis show the same direction of association in East Asians, South Asians and African Americans. These authors also described that for the majority of the loci, there was no evidence of heterogeneity in effect sizes between the European and non-European populations^5^. In another study of GWAS-identified SNPs associated with lipid traits, Dumitrescu *et al.*^9^ reported that the majority of the loci identified in European populations generalized to African Americans, American Indians and Hispanics. In particular, 14/27 (52%), 10/19 (53%) and 12/14 (86%) of the SNPs identified in European GWAS for HDL, LDL and TG were also associated at a significance threshold of p < 0.05 in the Hispanic sample. These observations also apply to other traits. In a recent trans-ethnic meta-analysis focused on type 2 diabetes^37^, the authors described that only 3 out of 69 established autosomal susceptibility loci showed evidence of heterogeneity in allelic effects. Additionally, 34 out of 52 previously reported lead SNPs showed the same direction of effects in European, East Asian, South Asian and Hispanic samples (65.4% vs 12.5% expected by chance, p < 2.2 × 10^−16^).

For the significant regions that were identified in both the GLGC European dataset^4^ and our Hispanic samples, we carried out a detailed analysis of the LD patterns between the European and Hispanic lead SNPs (Supplemental File 5). We also performed conditional analyses to gain further insights about the presence of shared vs. independent signals in these regions (Table 4). The extent of LD between the European and Hispanic lead SNPs shows substantial variation, from very strong (r^2^ > 0.8, *CELSR2*, *ZNF256/APOA5*, *TRIB1*, *GCKR*) to moderate (r^2^ > 0.25, *LPL* and *APOB*) to very weak or non-existent (r^2^ < 0.1, *NCAN/MAU2*, *ABCA1*, *LIPC* and *CETP*). The analysis of LD patterns indicates that trans-ethnic meta-analyses may be extremely useful for fine-mapping efforts, in accordance to what has been shown in previous studies^35^. The conditional analyses are fully consistent with the expectations based on the observed pattern of LD between markers. In the regions showing strong LD between the European and Hispanic lead SNPs, the p-values of the European and Hispanic lead SNPs are of similar magnitude in the Hispanic sample, and after conditioning on the Hispanic lead SNP, the European lead SNP becomes non-significant. In the regions showing moderate LD, the p-values of the European lead SNP also change considerably (*LPL*) or become non-significant (*APOB*) after conditioning. Importantly, for three regions (*ABCA1* and *LIPC* for HDL cholesterol and *NCAN/MAU2* for triglycerides) there is strong evidence for the presence of independent signals in the European and Hispanic samples (i.e. there are at least two independent SNPs influencing lipid levels in these regions): the European and Hispanic lead SNPs are not in LD, and after conditioning the p-values of the European lead SNP in the Hispanic sample are more significant than the original p-values. Interestingly, for *ABCA1* and *LIPC*, although the European lead SNP and our lead SNP are not in LD, our lead SNPs are in strong LD with the lead SNPs described recently in a study in Mexicans^11^. For *ABCA1*, the r^2^ between our lead SNP, rs2472386, and the lead SNP in the aforementioned study, rs2278426, is 0.65 in the 1000 Genomes MXL sample. For *LIPC*, the r^2^ between our lead SNP, rs261334, and the lead SNP reported in that study, rs1077835, is 0.83.

Our meta-analysis of the European and Hispanic samples identified five genome-wide significant regions that are novel for their associated lipid traits (Table 5). For total cholesterol, the regions correspond to the genes *FN1* on chromosome 2 and *SAMM50* on chromosome 22. The lead SNP in the *FN1* region, rs1250229, was associated with LDL cholesterol in the GLGC meta-analysis^4^. This region has also been associated with cardiovascular disease (CAD), heart failure and myocardial infarction in previous studies (STAMPEED, see web resources)^38^. The gene *SAMM50* has been associated with CAD (NHLBI Family Heart Study, see web resources) and with non-alcoholic fatty liver disease (NAFLD)^39^. NAFLD is a spectrum of conditions associated with lipid deposition in hepatocytes, which is strongly associated with metabolic syndrome^40^. The SNP that has been associated with NAFLD in the Japanese study, rs2143571, is in LD with the lead SNP in the European-Hispanic meta-analysis, rs2235776 (r^2^ = 0.548 in CEU, r^2^ = 0.728 in MLX). For HDL cholesterol, two regions located on chromosomes 6 (*LOC100996634*) and 11 (*COPB1*) also reached genome-wide significance. Finally, for LDL cholesterol, a missense variant (rs4791641) located in the gene *PFAS* also surpassed the genome-wide significance threshold. To our knowledge, none of these three genes have been previously associated with lipid concentrations or other relevant traits, such as CAD. Very little functional information is available for the gene *LOC100996634*. The *COPB1* gene encodes a protein subunit of the coatomer complex associated with non-clathrin coated vesicles. According to UniProt (see Web resources), this complex is involved in lipid homeostasis by regulating the presence of perilipin family members PLIN2 and PLIN3 at the lipid droplet surface and promoting the association of adipocyte surface triglyceride lipase (PNPLA2) with the lipid droplet to mediate lipolysis. The gene *PFAS* encodes an enzyme that plays a role in the synthesis of inosine monophosphate (IMP). The five novel loci identified in the meta-analysis of the European and Hispanic samples need to be replicated in independent samples.

We followed up in our samples the lead SNPs described in two recent GWA studies for lipid traits in Mexican samples^11^,^12^, which are very relevant to our own study due to the similarities in ancestral composition. Of particular interest are the novel genome-wide regions described in these studies, which are located near or at the genes *TMEM241*^11^ and *SIK3*^12^ for triglycerides, and *UGT8* and *RORA* for HDL cholesterol^12^. We could not replicate the associations at *TMEM241*, *UGT8* and *RORA*: none of the lead SNPs described for these regions is nominally significant in our dataset (Supplemental File 6). The lead SNP at *SIK3*, rs139961185, is genome-wide significant in our sample (p = 1.71 × 10^−8^). However, LD and conditional analyses indicate that the signal observed in this region is primarily driven by the well-known association at *ZNF259/APOA5*, which is located approximately 150 kb apart from the *SIK3* lead SNP on chromosome 11. The r^2^ value between rs139961185 and rs964184 in the 1000 Genomes MXL samples is 0.181, and this relatively low LD seems to explain why the p-values of rs139961185 in the original Mexican study (p = 1.15 × 10^−12^) and our dataset (1.71 × 10^−8^) are substantially lower than the p-values of rs964184 in both studies (6.08 × 10^−33^ and 5.32 × 10^−37^, respectively). If the signal observed at rs139961185 is truly independent of rs964184, we would expect that the p-values for this marker would remain significant after conditioning on rs964184 (similarly to what was described for the European lead SNPs at *ABCA1*, *LIPC* and *NCAN/MAU2*), but this is not the case in our samples: After conditioning on rs964184, the p-value of rs139961185 becomes non-significant (p = 0.922).

In addition to the standard association analyses using directly genotyped and imputed data, we carried out gene-based and pathway analyses, as well as a genome-wide association analysis restricted to eSNPs reported for LCL, adipose, muscle and human liver (Supplemental Files 7–9). The gene-based and eSNP association analyses are attractive because of the reduced penalty for multiple testing in comparison with standard GWA studies. The results of the gene-based and eSNP association tests are in agreement with those observed in the primary GWAs, although there are now some regions that were not genome-wide significant in the original meta-analysis, but reach genome-wide significance in the gene-based (using Bonferroni correction) or eSNP association (using the FDR criterion) tests. For example, in the gene-based tests, genes in the *ZNF256/APOA5* region and the gene *RPL34* are genome-wide significant for total cholesterol, and the genes *LPL* and *LIPC* are genome-wide significant for HDL cholesterol (Supplemental File 7). These regions did not reach genome-wide significance in the original meta-analysis. Similarly, in the eSNP association tests, using an FDR of 5%, eSNPs in the *LPL*, *LIPC* and *LOC102467079* regions are genome-wide significant for HDL cholesterol, and eSNPs located in the *DOK6* and *TRIB1* regions are genome-wide significant for LDL cholesterol and triglycerides, respectively (Supplemental File 9). Of note, several eSNPs identified as significant in our analysis are either the lead SNPs of our meta-analysis (rs2238675 is our lead SNP in the *NCAN* region for total cholesterol, rs7499892 is our lead SNP in the *CETP* region for HDL cholesterol and rs660240 is our lead SNP in the *CELSR2* region for LDL cholesterol), or have p-values quite similar to those of our lead SNP (*CELSR2* eSNPs for total cholesterol, *CELSR2* and *DOK6* eSNPs for LDL cholesterol, *ABCA1, ZNF256/APOA5, CETP, LPL, LIPC* and *LOC102467079* eSNPs for HDL cholesterol, and *TRIB1* for triglycerides), or are the lead SNPs of the GLGC meta-analysis (rs646776 is the lead SNP in the *CELSR2* region for total cholesterol and LDL cholesterol). Thus, it is possible that at least some of the top meta-analysis signals exert a phenotypic effect through transcriptional regulation. Furthermore, the eQTLs reported for each tissue showed different levels of enrichment for top associations with lipids traits. For example, eQTLs identified in adipose, muscle and liver showed consistent departure from the null for LDL cholesterol and total cholesterol; in contrast, those mapped in LCLs did not reach significance for these traits (although they were highly significant for the remaining lipids phenotypes) (Figure S8). In general, however, the eQTLs mapped in these tissues led to improved false discovery rate. Additionally, the target genes they implicate are tissue-dependent (suggesting tissue-specific function) and provide plausible biological mechanisms for the observed associations.

As expected, the pathway-based tests showed a significant enrichment in pathways involved in lipid metabolism. Of note is the enrichment observed for gene sets involved in chylomicron mediated lipid transport, lysosomal degradation of glycoproteins (more particularly, degradation of chondroitin sulfate and dermatan sulfate), peroxisome proliferator-activated receptor-γ (PPARγ) signaling, as well as cell adhesion molecules and ABC transporters, for multiple lipid traits (Supplemental Files 7 and 8).

Our meta-analysis of lipid traits is one the largest efforts to characterize the architecture of lipid traits in Hispanics. However, it is important to mention some of the limitations of this study. Our sample size (4,383 individuals in the discovery sample and 7,876 in the replication cohorts) is still relatively small compared with similar efforts in European populations^4^. This implies that our discovery effort was only powered to identify loci with relatively large effects on lipid traits. Additionally, we should also note the potential consequences of differences in ancestral contributions between the discovery and replication samples. The three samples that were used for SNP discovery (Mexico City 1 and 2, and Starr County) primarily show Native American and European admixture, with very little evidence of an African contribution (see PCA plots in supplementary information). However, the replication samples include Hispanic individuals of Mexican ancestry and also of Caribbean origin. In particular, the BioMe Hispanics are predominantly from Puerto Rican, Dominican and Central/South American origin. It is known that there is substantial heterogeneity in the ancestral composition of Hispanic populations, with widely different Native American, European and African contributions depending on the geographic origin of the Hispanic individuals^41–43^. This heterogeneous ancestral composition may be associated with differential patterns of LD in the genomic regions being studied, which in turn may have a negative impact in our ability to replicate the signals identified in the discovery samples. Many of the markers that were followed up in the replication cohorts showed evidence of heterogeneity of effect sizes in the meta-analysis (see Table 2 and Supplemental File 3). Finally, the three discovery samples include both controls and individuals with type 2 diabetes. Here, we have reported the results obtained when including diabetes status as a covariate, but the results are quite similar when analyzing the data without diabetes status as a covariate.

In summary, we report here the results of a meta-analysis of lipid traits in three samples of Mexican ancestry, and the follow-up of the genome-wide and suggestive signals in independent Hispanic samples. We observed genome-wide significant signals in multiple regions that have been previously described in European studies. LD and conditional analyses indicate that for some of these regions we are capturing the same signals present in European populations. However, in other regions (*ABCA1* and *LIPC* for HDL cholesterol, and *NCAN/MAU2* for triglycerides), our lead SNPs are clearly independent of the lead SNPs described in European populations. We also identified a novel region between the genes *ACTBP2/AP3B1* that was borderline genome-wide significant for triglycerides. Our meta-analysis of the European GLGC and Hispanic datasets highlighted five novel regions associated with lipid traits. Further GWAS for lipid traits are needed in Hispanic populations, which show high prevalence of dyslipidemias. These studies will make it possible to identify genomic regions that are relevant for these populations, and will also facilitate the fine mapping of lipid regions by combining datasets from populations of diverse ancestry.

## Additional Information

**How to cite this article**: Below, J. E. *et al.* Meta-analysis of lipid-traits in Hispanics identifies novel loci, population-specific effects, and tissue-specific enrichment of eQTLs. *Sci. Rep.* **6**, 19429; doi: 10.1038/srep19429 (2016).

## Supplementary Material

Supplementary Information

Supplementary dataset 1

Supplementary dataset 2

Supplementary dataset 3

Supplementary dataset 4

## Figures and Tables

**Table 1:** Descriptive information about the three Hispanic samples included in the meta-analysis of lipid traits.

Sample	Mexico City 1*	Mexico City 2*	Starr County
N (F, M)	1310 (833,477)	1787 (895,892)	1286 (858,428)
Age (SD)	50.57 (8.269)	52.78 (10.12)	45.27 (13.25)
BMI	29.06 (4.64)	28.64 (4.91)	30.73 (6.42)
TCHOL (mg/dl)	211.30 (43.38)	190.16 (44.73)	194.13 (41.24)
HDL	46.04 (14.61)	45.19 (14.12)	47.38 (14.52)
LDL	132.07 (35.15)	130.04 (34.59)	114.43 (32.12)
TRIG	212.43 (142.11)	174.99 (113.39)	168.73 (131.91)
T2D Cases/Controls	967/343	898/889	536/750

^*^Average lipid values reflect lipid concentrations without correction for lipid-lowering treatment.

**Table 2:** Genome-wide significant signals identified in the meta-analysis of the three Hispanic samples (Mexico City samples 1 and 2, and Starr County).

GENE	CHR	POS	SNP	NEA	EA	P-META	BETA	SE	P-HET	P-SC	EAF-SC	P-MC1	EAF-MC1	P-MC2	EAF-MC2
TCHOL															
* CELSR2*	1	109817192	rs7528419	G	A	7.88E-12	0.192	0.028	0.361	6.89E-07	0.812	0.003	0.817	9.60E-05	0.823
* NCAN*	19	19336608	rs2238675	T	C	1.40E-08	0.197	0.035	0.579	0.061	0.901	2.09E-04	0.865	6.23E-05	0.874
HDL															
* ABCA1*	9	107601541	rs2472386	G	A	5.13E-10	0.140	0.023	0.651	0.001	0.473	0.009	0.416	2.22E-06	0.427
* ZNF259*/*APOA5*	11	116581641	rs2367970	A	G	5.82E-09	0.149	0.026	0.156	0.053	0.778	0.013	0.739	1.41E-07	0.739
* CETP*	16	57006590	rs7499892	T	C	4.16E-17	0.233	0.028	0.443	0.001	0.783	6.42E-05	0.775	2.77E-11	0.780
LDL															
* CELSR2*	1	109817838	rs660240	T	C	1.19E-12	0.203	0.029	0.551	2.89E-06	0.812	1.53E-04	0.818	8.35E-05	0.822
TRIG															
* ZNF259*/*APOA5*	11	116648917	rs964184	C	G	5.32E-37	0.289	0.023	0.600	6.12E-09	0.248	1.25E-15	0.364	4.44E-16	0.367
* MAU2*	19	19455750	rs8102280	A	G	9.86E-10	0.251	0.041	0.492	0.214	0.956	3.92E-05	0.907	6.37E-06	0.909

NEA: Non-effect allele, EA: Effect allele, P-META: P-value of the fixed effects meta-analysis in META, SE: Standard Error, P-HET: Heterogeneity P-value, P-SC: P-value Starr County, P-MC1: P-value Mexico City sample 1, P-MC2: P-value Mexico City sample 2, EAF: Effect allele frequency, TCHOL: Total cholesterol, HDL: HDL cholesterol, LDL: LDL cholesterol, TRIG: Triglycerides.

**Table 3:** Genome-wide signals identified in the follow-up of the genome-wide and suggestive signals observed in this study in three independent Hispanic samples (WHI, MESA and BioMe).

GENE	CHR	POS	LEAD SNP	NEA	EA	P-THIS STUDY (N = 4383)	EAF MC2*	P-WHI (N = 3587)	EAF WHI	P-MESA (N = 2127)	EAF MESA	P-BioMe (N = 2162)	EAF BioMe	P-META (N = 12259)	BETA	SE	P-HET	EFFECTS
TCHOL																		
*CELSR2*	1	109817192	rs7528419	G	A	7.88E-12	0.823	3.01E-06	0.792	3.81E-12	0.777	0.003	0.764	9.05E-27	0.172	0.016	0.013	++++
*ZNF259/APOA5*	11	116648917	rs964184	C	G	3.19E-07	0.367	5.50E-05	0.248	0.055	0.292	0.005	0.216	4.88E-13	0.103	0.014	0.674	++++
*KANK2/DOCK6*	19	11300365	rs138534124	C	T	2.89E-06	0.834	0.203	0.925	1.47E-04	0.927	0.339	0.964	7.86E-09	0.148	0.026	0.137	++++
*NCAN*	19	19336608	rs2238675	T	C	1.40E-08	0.874	2.73E-04	0.887	0.002	0.888	0.068	0.892	1.23E-13	0.164	0.022	0.399	++++
HDL																		
*LPL*	8	19891227	rs28526159	C	T	8.49E-08	0.462	0.132	0.618	0.140	0.615	0.010	0.703	8.41E-09	0.077	0.013	0.088	++++
*ABCA1*	9	107601541	rs2472386	G	A	5.13E-10	0.427	9.91E-04	0.436	0.005	0.460	0.829	0.488	8.37E-11	0.088	0.014	0.002	+++-
*ZNF259/APOA5*	11	116581641	rs2367970	A	G	5.82E-09	0.739	0.031	0.776	0.006	0.756	0.484	0.789	8.42E-10	0.097	0.016	0.034	++++
*LIPC*	15	58726744	rs261334	C	G	1.19E-07	0.587	0.004	0.384	1.08E-06	0.425	0.381	0.335	9.40E-13	0.094	0.013	0.019	++++
*CETP*	16	57006590	rs7499892	T	C	4.16E-17	0.780	2.64E-18	0.773	4.79E-17	0.754	3.78E-05	0.726	3.80E-49	0.243	0.016	8.83E-04	++++
LDL																		
*CELSR2*	1	109817838	rs660240	T	C	1.19E-12	0.822	7.27E-08	0.793	2.346E-13	0.777	0.022	0.745	1.25E-28	0.179	0.016	0.002	++++
*APOB*	2	21217490	rs13392272	C	T	6.01E-07	0.364	1.82E-05	0.387	0.026	0.343	0.059	0.350	2.16E-12	0.095	0.014	0.497	++++
*NCAN*	19	19336608	rs2238675	T	C	4.75E-06	0.874	0.019	0.887	0.032	0.888	0.129	0.892	2.33E-08	0.125	0.022	0.555	++++
TRIG																		
*GCKR*	2	27742603	rs780093	C	T	6.03E-06	0.304	9.61E-09	0.356	0.003	0.326	0.039	0.306	2.82E-15	0.108	0.014	0.320	++++
*TRIB1*	8	126491733	rs2954031	T	G	7.51E-07	0.649	4.50E-05	0.612	0.018	0.616	0.006	0.620	3.26E-13	0.098	0.013	0.791	++++
*ZNF259/APOA5*	11	116648917	rs964184	C	G	5.32E-37	0.367	2.07E-28	0.248	4.102E-17	0.292	3.27E-07	0.216	2.79E-83	0.275	0.014	0.044	++++
*MAU2*	19	19455750	rs8102280	A	G	9.86E-10	0.909	2.11E-07	0.956	0.005	0.953	0.017	0.954	3.38E-18	0.252	0.029	0.497	++++

^*^For the discovery sample, we show the allele frequency observed in the largest sample (Mexico City 2).

For the individual studies, we report the p-values and the effect allele frequencies (EAF). For the meta-analysis, we report the p-values (P), beta coefficients (BETA) and standard errors (SE), heterogeneity p-value (P-HET), and direction of effect. Complete information of all the markers that were followed up is provided in Supplementary File 3.

**Table 4:** Results of the conditional analysis evaluating the lead SNP reported by the European GLGC consortium before and after conditioning on the lead SNP identified in the Hispanic sample.

							RESULTS CONDITIONAL ANALYSIS		
GENE	CHR	CONDITIONING SNP (HISPANIC LEAD SNP)	TESTED SNP (GLGC LEAD SNP)	NEA	EA	P-VALUE TESTED SNP IN HISPANIC SAMPLE	P-COND	BETA-COND	SE-COND
TCHOL									
* CELSR2*	1	rs7528419	rs646776	C	T	3.91E-11	0.916	−0.003	0.028
* TRIB1*	8	rs2980885	rs2954029	A	T	1.44E-05	0.012	−0.056	0.022
* ZNF259*	11	rs964184	rs964184			3.19E-07			
* NCAN*	19	rs2238675	rs10401969	T	C	7.77E-05	0.003	−0.161	0.054
HDL									
* LPL*	8	rs28526159	rs13702	T	C	4.38E-07	0.009	0.065	0.025
* ABCA1*	9	rs2472386	rs1883025	C	T	1.94E-03	1.80E-05	−0.102	0.024
* ZNF259*	11	rs2367970	rs964184	G	C	1.09E-08	0.004	0.065	0.023
* LIPC*	15	rs261334	rs10468017	C	T	2.13E-04	2.65E-06	0.141	0.030
* CETP*	16	rs7499892	rs247616	C	T	7.61E-11	4.64E-04	0.083	0.024
LDL									
* CELSR2*	1	rs660240	rs646776	C	T	8.36E-12	0.847	−0.005	0.028
* APOB*	2	rs13392272	rs1367117	G	A	5.57E-06	0.832	−0.006	0.029
* NCAN*	19	rs2238675	rs10401969	T	C	4.45E-03	0.045	−0.111	0.055
TRIG									
* GCKR*	2	rs780093	rs1260326	T	C	4.22E-05	0.782	0.006	0.023
* TRIB1*	8	rs2954031	rs2954022	C	A	2.21E-06	0.956	−0.001	0.022
* ZNF259*	11	rs964184	rs10790162	A	G	3.80E-24	0.071	−0.051	0.028
* MAU2*	19	rs8102280	rs10401969	T	C	2.99E-04	5.42E-05	−0.219	0.054

The table shows the non-effect and effect alleles (NEA, EA), original p-value of the tested SNP (GLGC lead SNP) in the Hispanic sample, and the p-value (P-COND), beta (BETA-COND) and SE (SE-COND) after conditioning on the Hispanic lead SNP.

**Table 5:** Genome-wide signals identified in the meta-analysis of the European GLGC dataset and the Hispanic dataset.

GENE	CHR	SNP	POS	NEA	EA	P-META	BETA	SE	EFFECT	P-HISP	P-EUR
TCHOL											
* FN1*	2	rs1250229	216304384	T	C	1.04E-08	0.023	0.004	++	1.75E-04	2.38E-07
* SAMM50*	22	rs2235776	44377999	T	C	2.85E-08	0.026	0.005	++	8.96E-04	9.94E-06
HDL											
* LOC100996634*	6	rs884366	109574095	A	G	1.40E-08	0.021	0.004	++	0.026	1.67E-07
* COPB1*	11	rs7121538	14504463	A	C	2.11E-08	0.026	0.005	++	0.017	4.47E-07
LDL											
* PFAS*	17	rs4791641	8161149	T	C	1.13E-08	0.021	0.004	++	0.034	1.31E-07

We report the p-value, beta and standard errors (SE) in the meta-analysis, as well as the p-values in the Hispanic and European datasets.
