# Supplementary material for: Meta-analysis of lipid-traits in Hispanics identifies novel loci, population-specific effects, and tissue-specific enrichment of eQTLs
Source: Sci Rep. 2016 Jan 19;6:19429. doi: 10.1038/srep19429 (PMC4726092; doi:10.1038/srep19429)
Supplement: Supplementary Information [file srep19429-s1.pdf]

Supplemental Figures

Meta-analysis of lipid-traits in Hispanics identifies novel loci, population-specific effects, and tissue-specific enrichment of eQTLs

Jennifer E. Below[1,13]*, Esteban J. Parra[2,13]**, Eric R. Gamazon[3], Jason Torres[3], S. Krithika[2], Sophie Candille[4], Yingchang Lu[5,6], Ani Manichakul[7], Jesus Peralta-Romero[8], Qing Duan[9], Yun Li[9], Andrew P. Morris[10], Omri Gottesman[5], Erwin Bottinger[5], Xin-Qun Wang[11], Kent D. Taylor[12], Y.-D. Ida Chen[12], Jerome I. Rotter[12], Stephen S. Rich[7], Ruth J.F Loos[5,6], Hua Tang[4], Nancy J. Cox[3], Miguel Cruz[8], Craig L. Hanis[1], Adan Valladares-Salgado[8].

[1]Division of epidemiology, Human Genetics & Environmental Sciences, University of Texas School of Public Health, Houston, Texas, USA.

[2]Department of Anthropology, University of Toronto at Mississauga, Mississauga, Ontario, Canada.

[3]Section of Genetic Medicine, Department of Medicine, University of Chicago, Illinois, USA.

[4]Department of Genetics, Stanford University School of Medicine, Stanford, California, USA

[5]The Charles Bronfman Institute for Personalized Medicine, The Icahn School of Medicine at Mount Sinai, New York, New York, USA.

[6]The Genetics of Obesity and Related Metabolic Traits Program, The Icahn School of Medicine at Mount Sinai, New York, New York, USA.

[7]Center for Public Health Genomics, University of Virginia, Charlottesville, Virginia, USA

[8]Unidad de Investigación Médica en Bioquímica, Hospital de Especialidades, Centro Médico Nacional Siglo XXI, IMSS, Mexico City, Mexico.

[9]Deparment of Genetics and Department of Biostatistics, University of North Carolina at Chapel Hill, Chapel Hill, North Carolina, USA.

[10]Wellcome Trust Centre for Human Genetics, University of Oxford, Oxford, United Kingdom

[11]Department of Public Health Sciences, University of Virginia, Charlottesville, Virginia, USA.

[12]Institute of Translational Genomics and Population Sciences, Los Angeles Biomedical Research Institute at Harbor/UCLA Medical Center, Torrance, California, USA.

[13]These authors contributed equally to this work, and are co-corresponding authors of this manuscript

* jennifer.e.below@uth.tmc.edu

** esteban.parra@utoronto.ca

SUPPLEMENTAL FIGURES:

Figure S1. Principal Component (PC) plots of Hispanic samples included in this study.

Figure S1A) PC plot of Mexico City samples with relevant reference samples: Axis 1 vs. Axis 2.

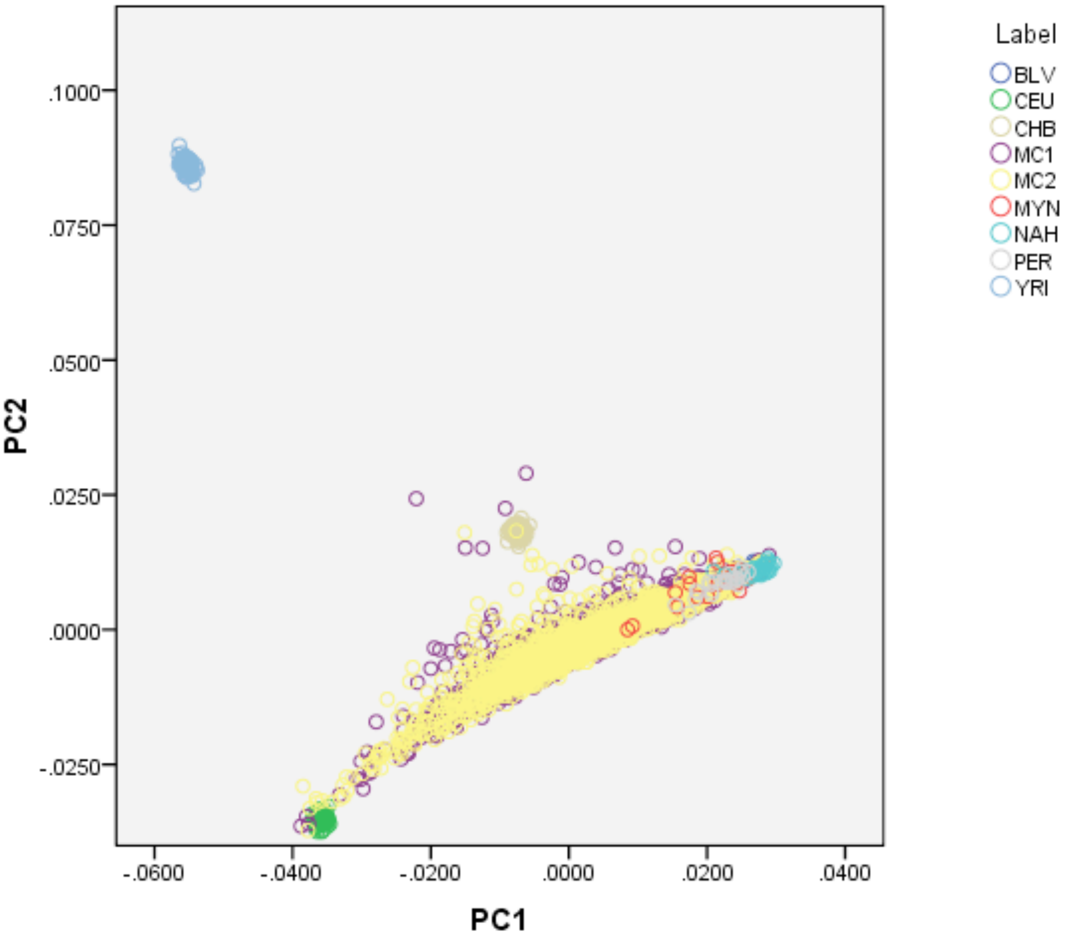

Abbreviations: BLV (Bolivia), CEU (Utah Residents), CHB (Han Chinese from Beijing), MC1 (Mexico City sample 1), MC2 (Mexico City sample 2), MYN (Mayan), NAH (Nahua), PER (Peru), YRI (Yoruba in Ibadan, Nigeria)

Figure S1B) PC plot of Mexico City samples with relevant reference samples: Axis 1 vs. Axis 3.

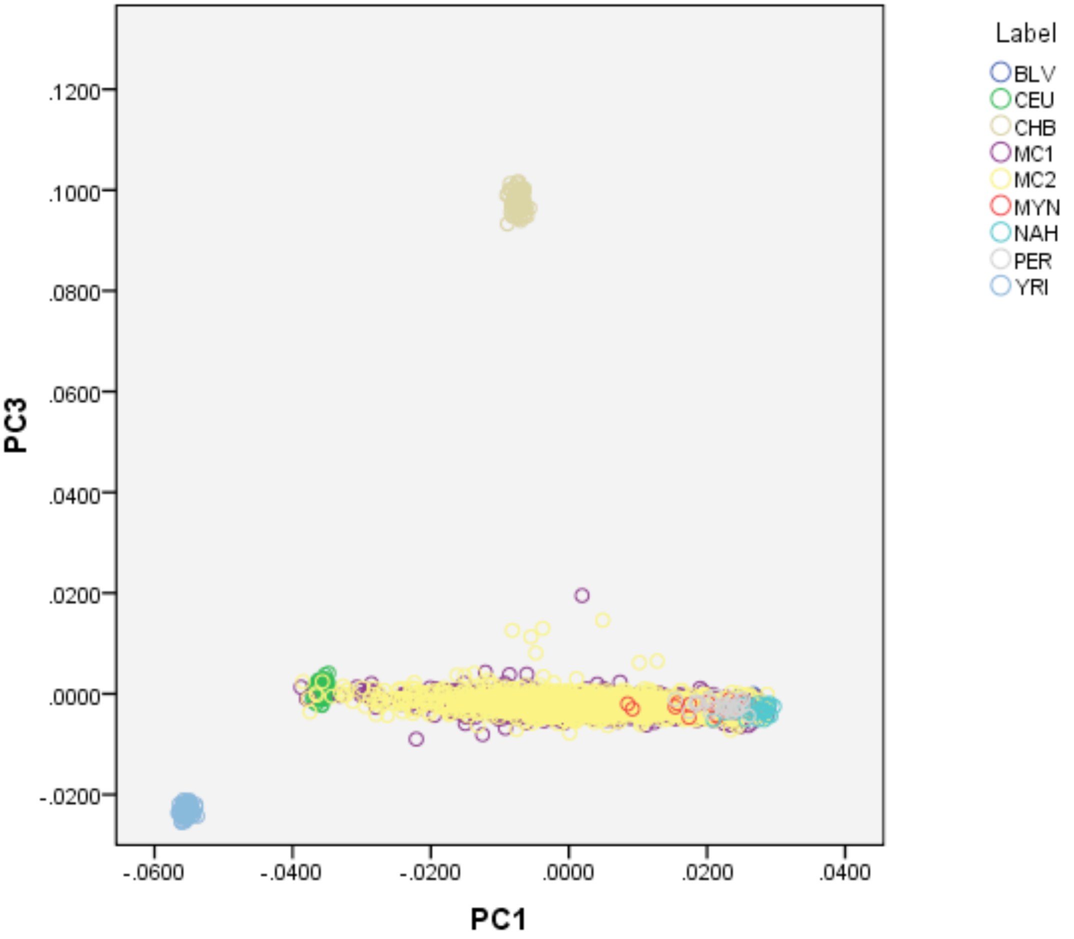

Figure S1C) PC plot of Mexico City samples with relevant reference samples: Axis 1 vs. Axis 4.

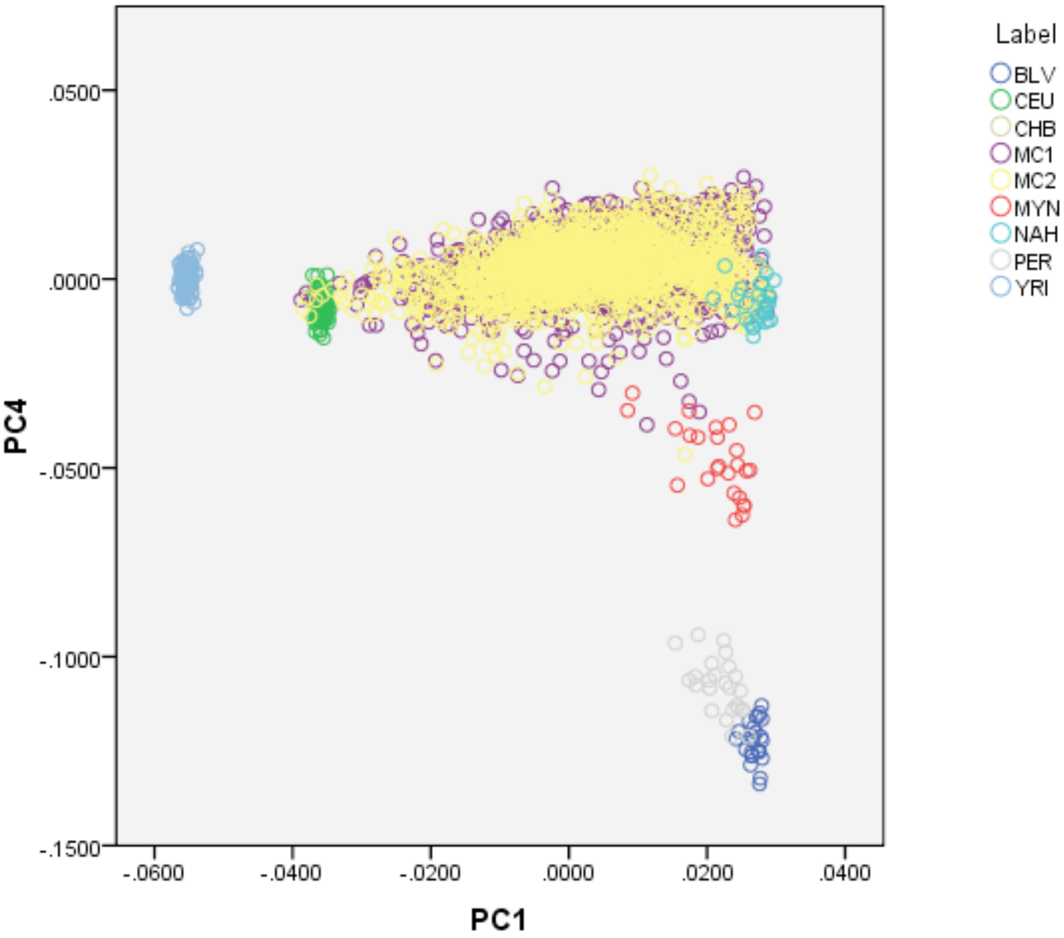

Figure S1D) PC plot of Starr County samples with relevant reference samples. Axis 1 vs. Axis 2.

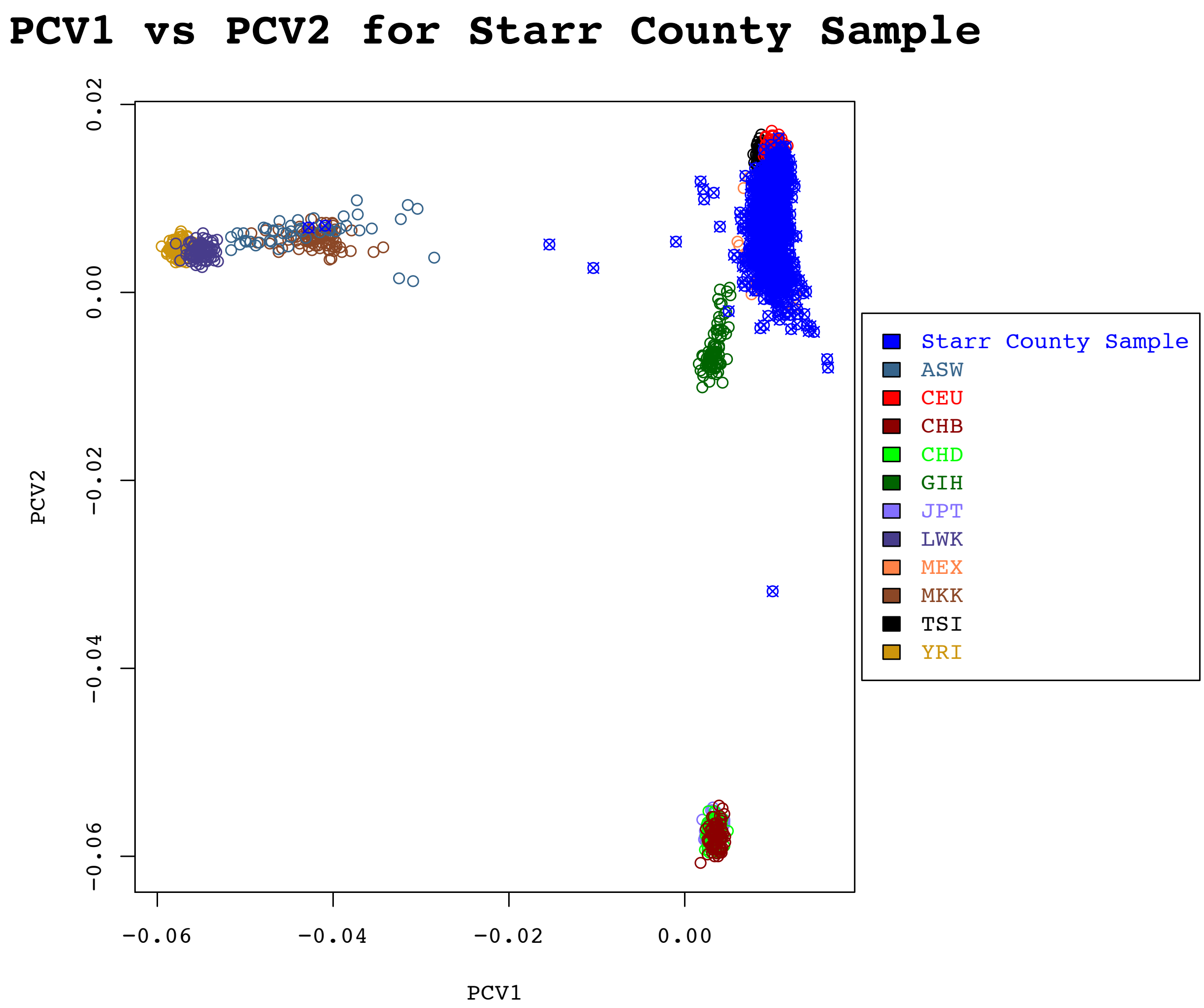

Abbreviations: ASW (Americans of African ancestry in SW USA), CEU (Utah residents), CHB (Han Chinese from Beijing), CHD (Chinese in Metropolitan Denver, Colorado), GIH (Gujarati Indian from Houston, Texas), JPT (Japanese in Tokyo, Japan), LWK (Luhya in Webuye, Kenya), MEX (Mexican ancestry from Los Angeles, USA), MKK (Maasai in Kinyawa, Kenya), TSI (Tuscans in Italy), YRI (Yoruba in Ibadan, Nigeria)

Figure S1E) PC plot of Starr County samples with relevant reference samples. Axis 1 vs. Axis 3.

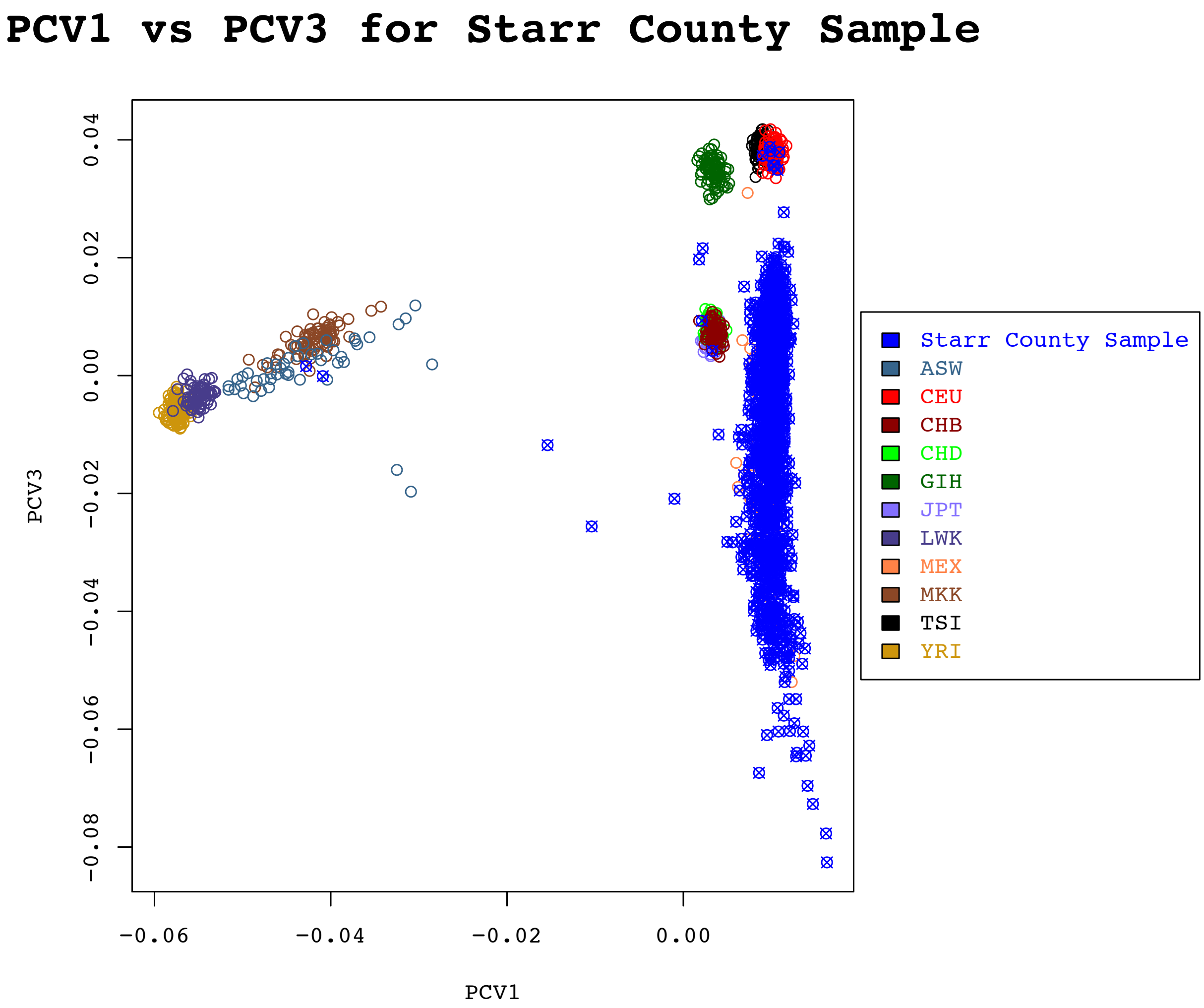

Figure S1F) PC plot of Starr County samples with relevant reference samples. Axis 1 vs. Axis 4.

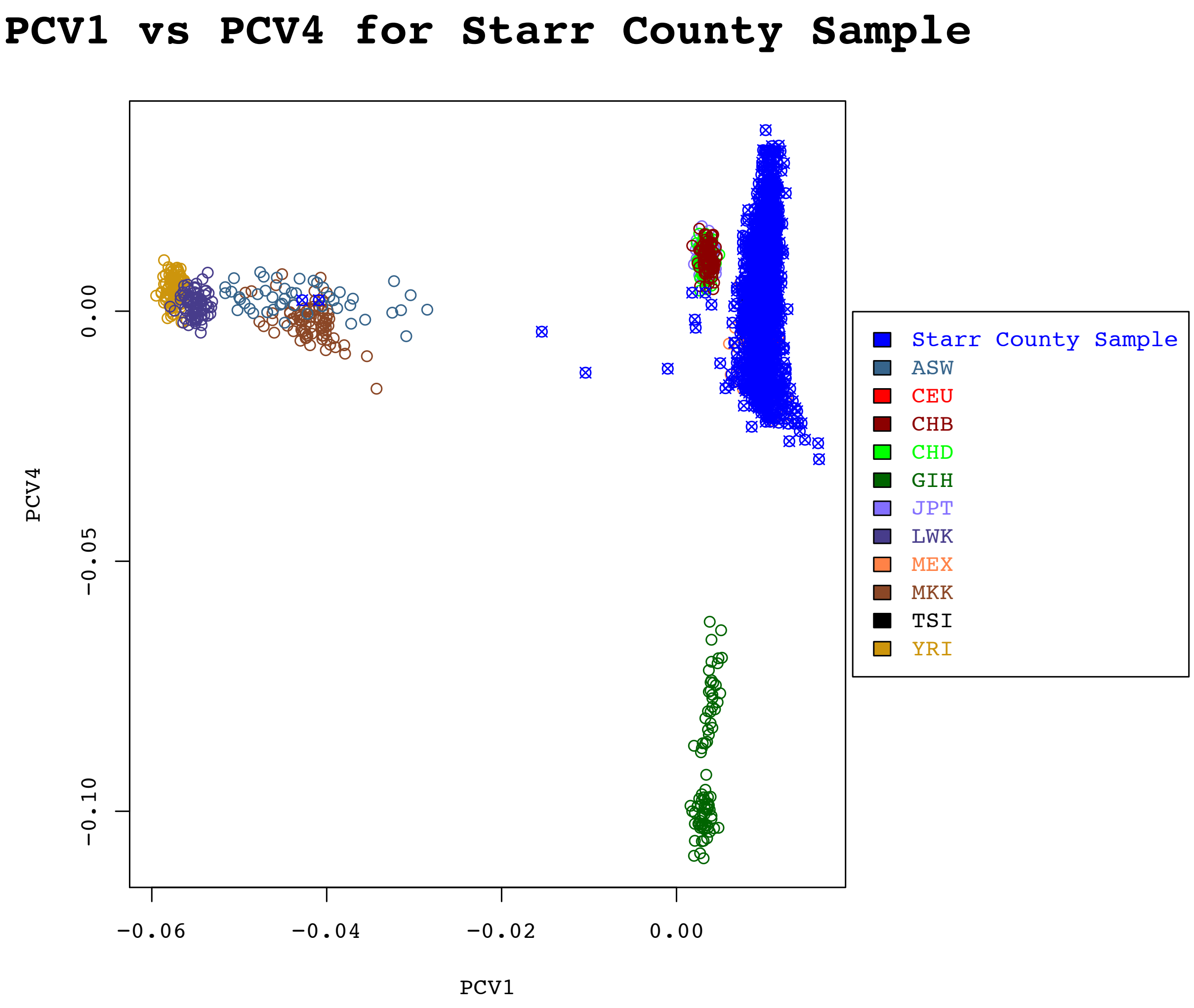

Figure S2. Manhattan plots of meta analysis results.

Figure S2A) Manhattan plot of total cholesterol meta analysis results.

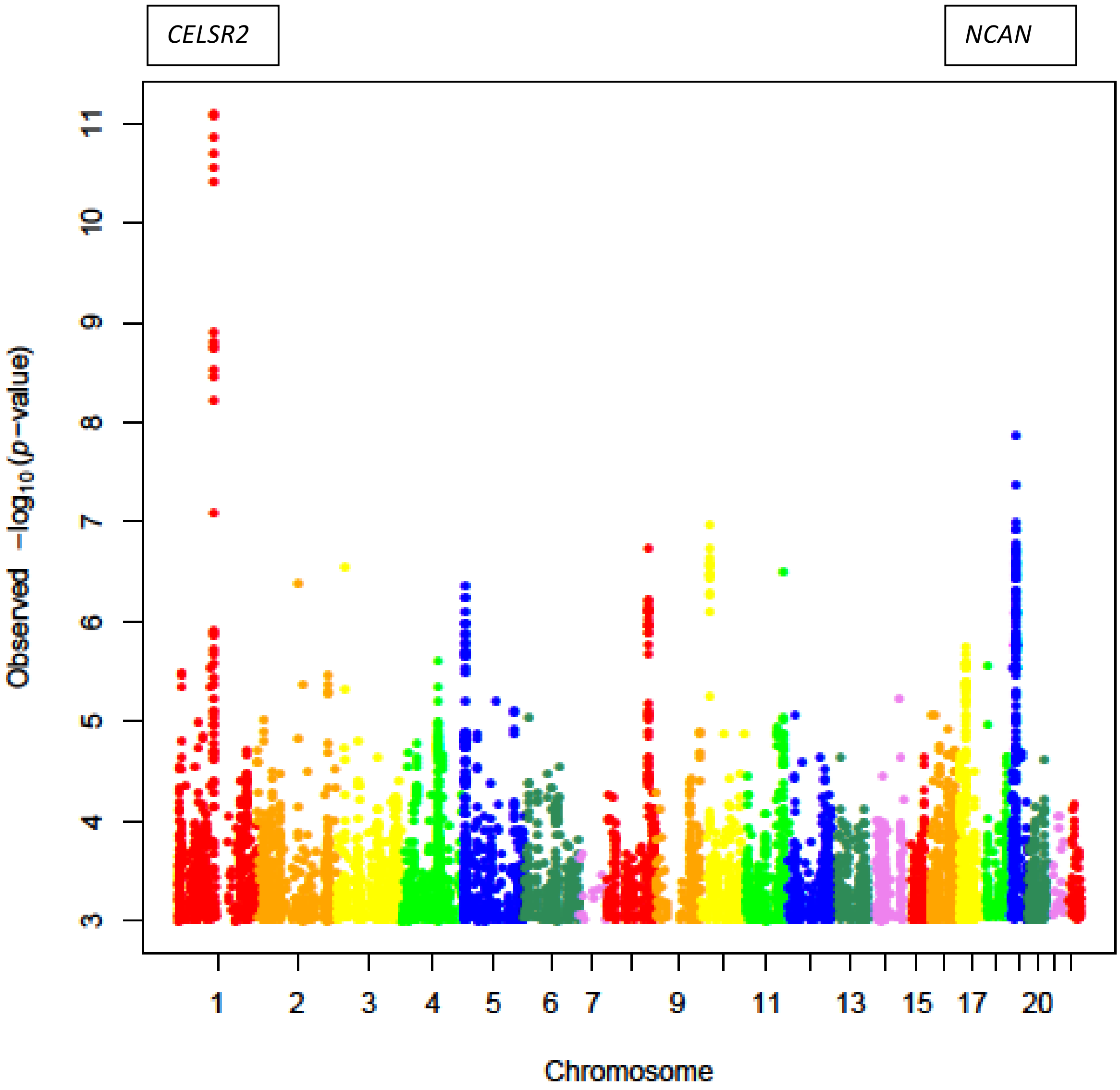

Figure S2B) Manhattan plot of HDL cholesterol meta analysis results.

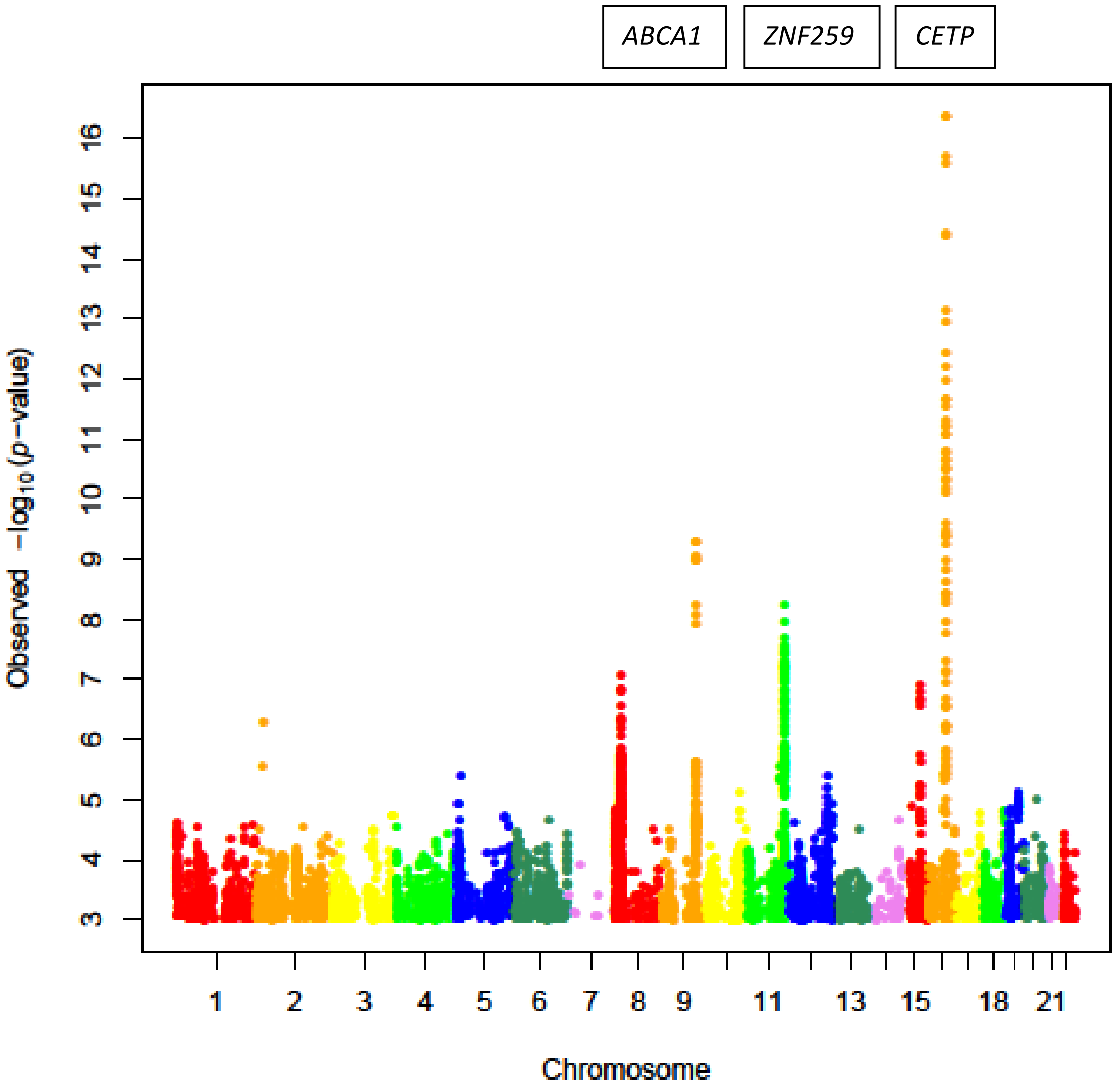

Figure S2C) Manhattan plot of LDL cholesterol meta analysis results.

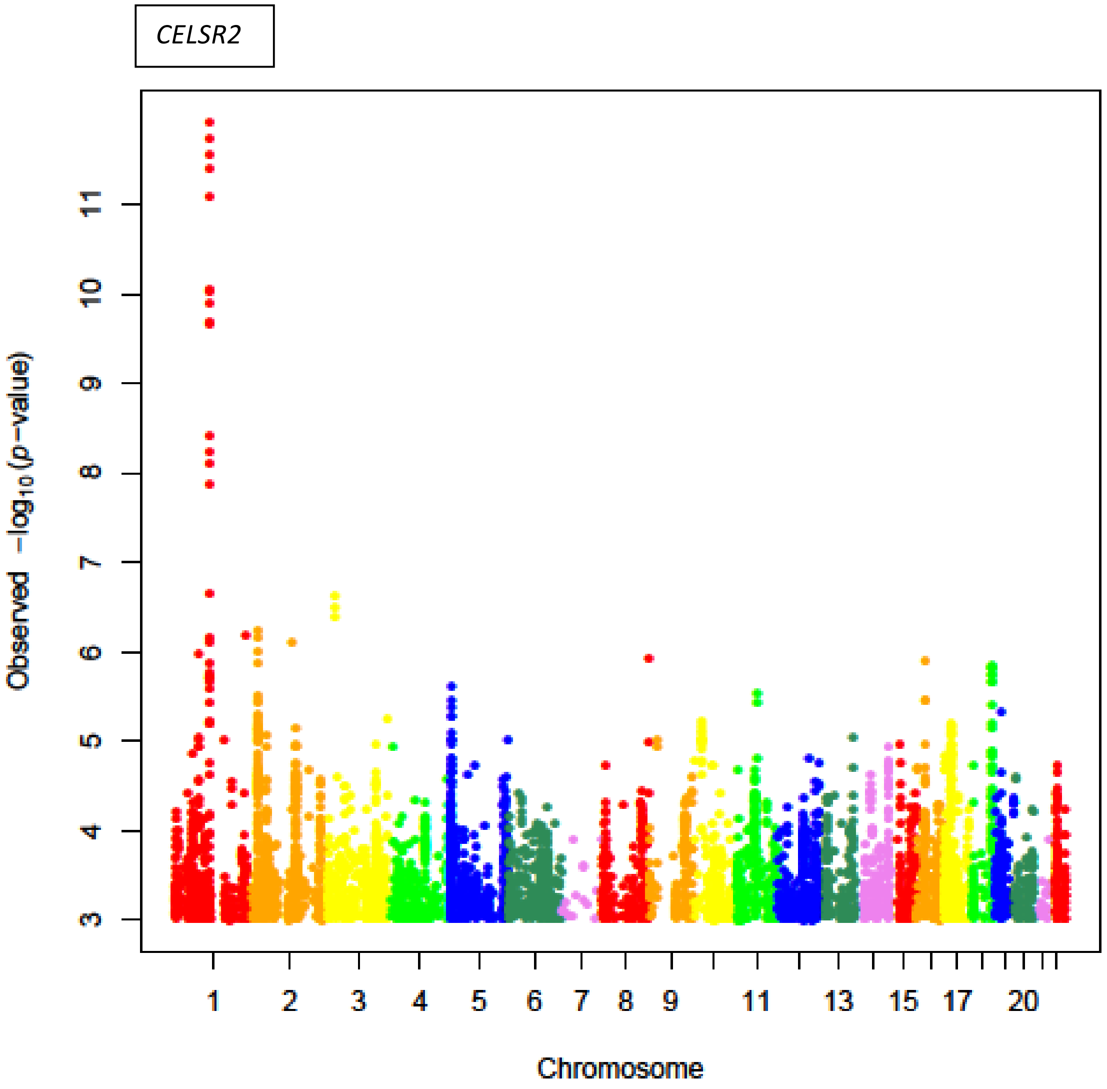

Figure S2D) Manhattan plot of triglycerides meta analysis results.

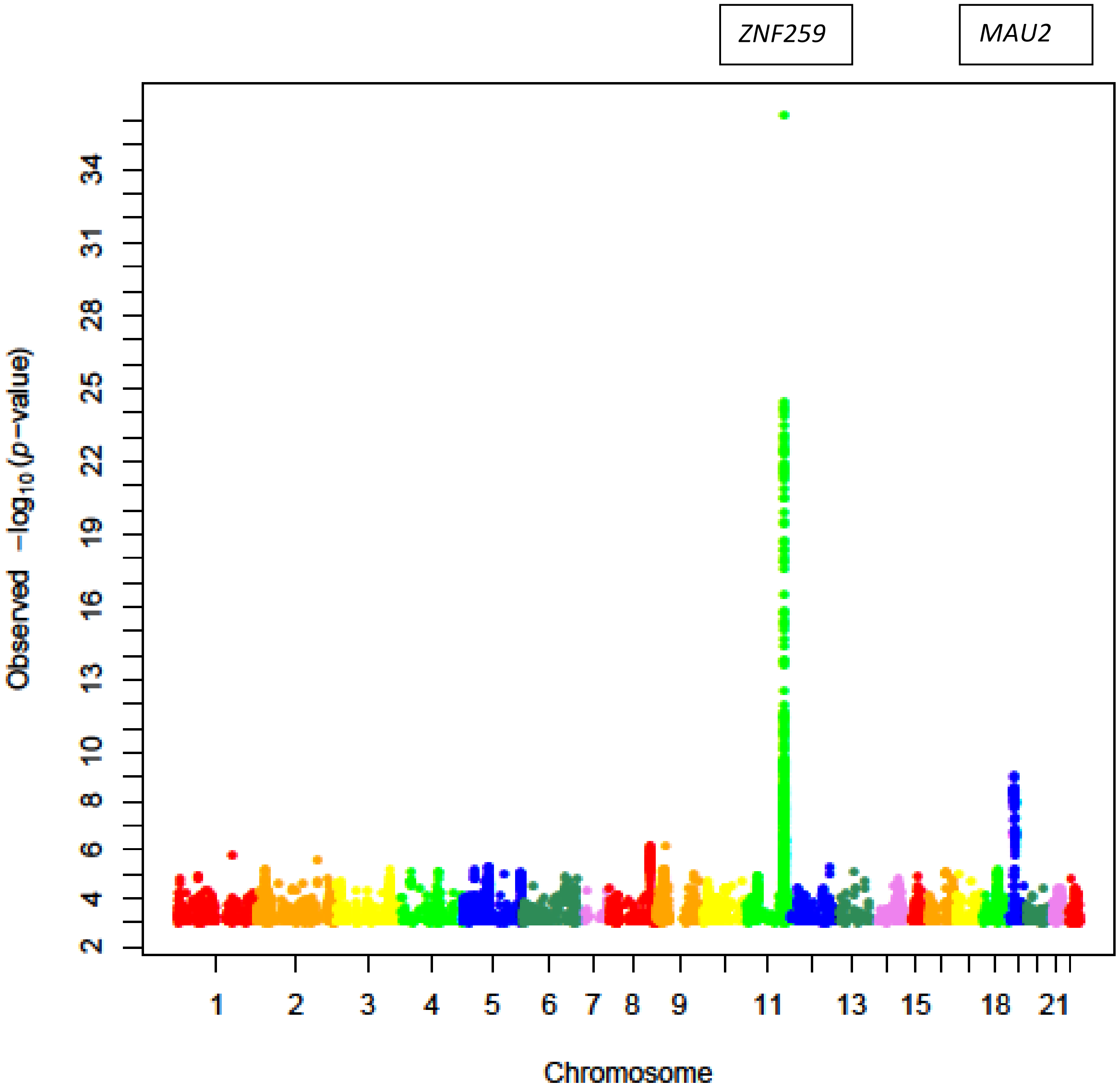

Figure S3. QQ plots.

Figure S3A) QQ plot of total cholesterol meta analysis results.

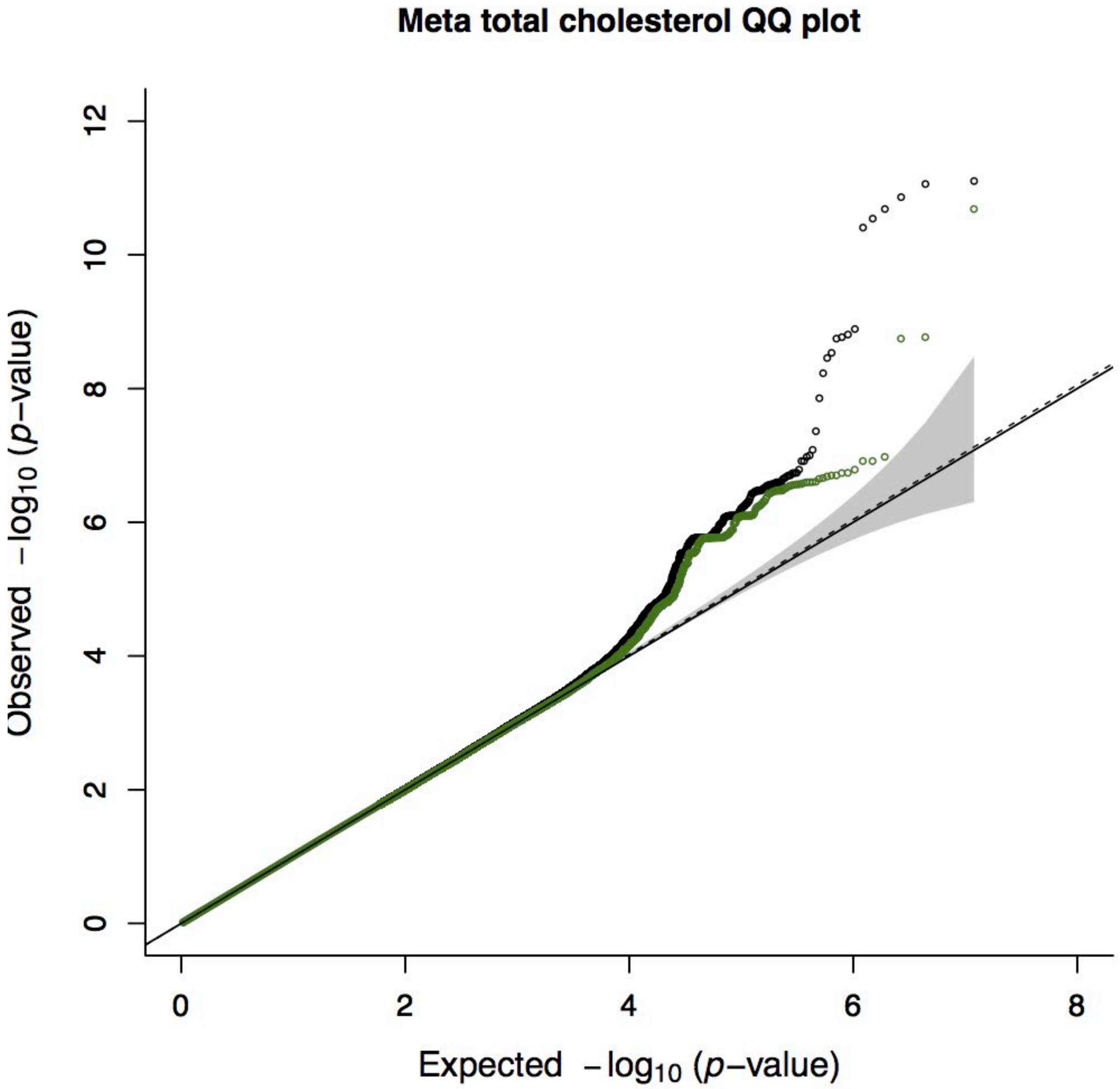

These plots show two lines. The black line corresponds to the complete meta-analysis dataset. The green line corresponds to markers remaining after excluding known genome-wide signals and markers in LD with these signals. The threshold used was an r^2 value of 0.2 for the CEU 1000 Genomes sample. There is no evidence of genome-wide inflation for any trait: Lambda values are 1.01 for total cholesterol, HDL cholesterol, and triglycerides, and 1.00 for LDL cholesterol.

Figure S3B) QQ plot of HDL cholesterol meta analysis results.

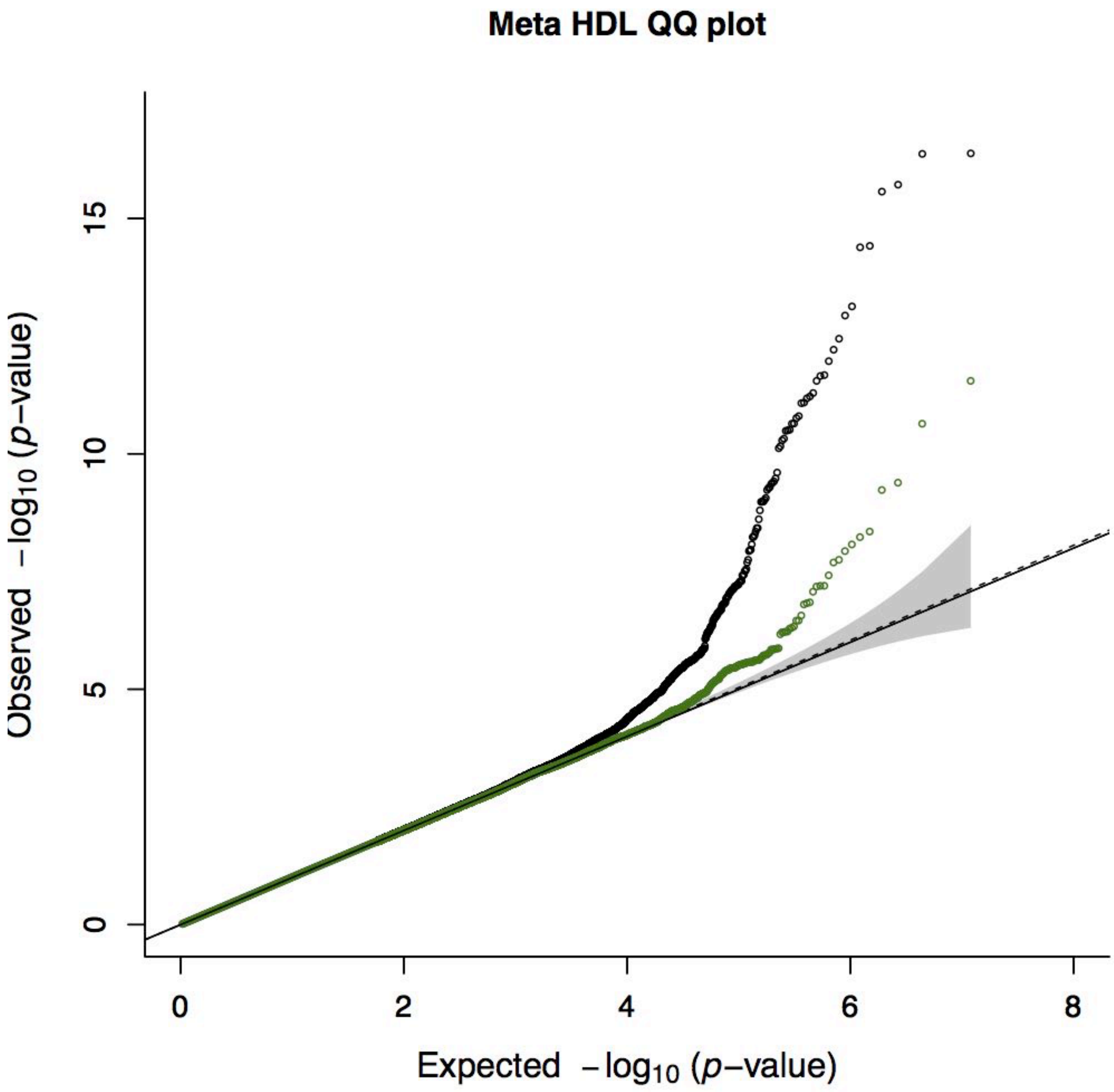

Figure S3C) QQ plot of LDL cholesterol meta analysis results.

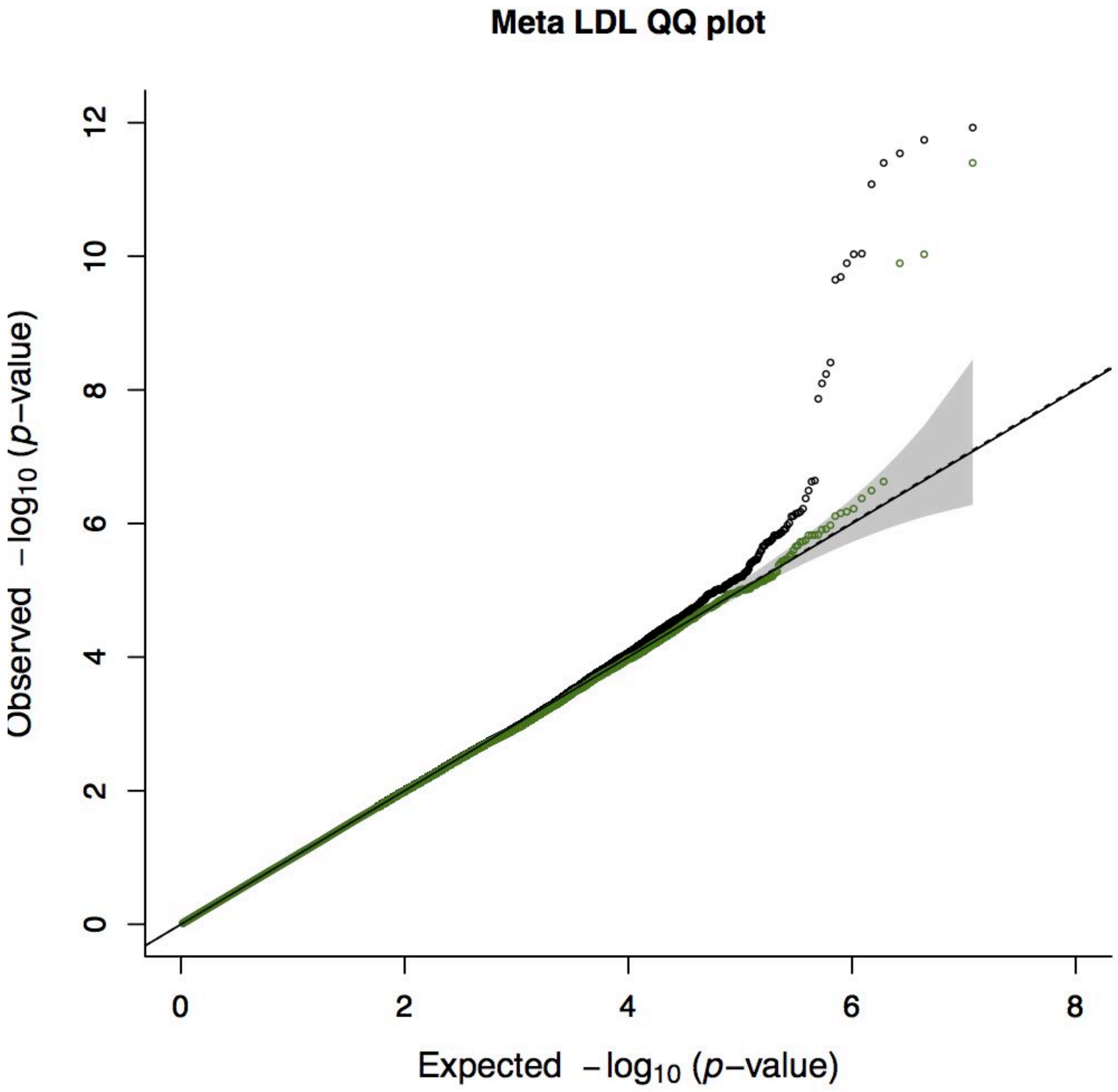

Figure S3D) QQ plot of triglycerides meta analysis results.

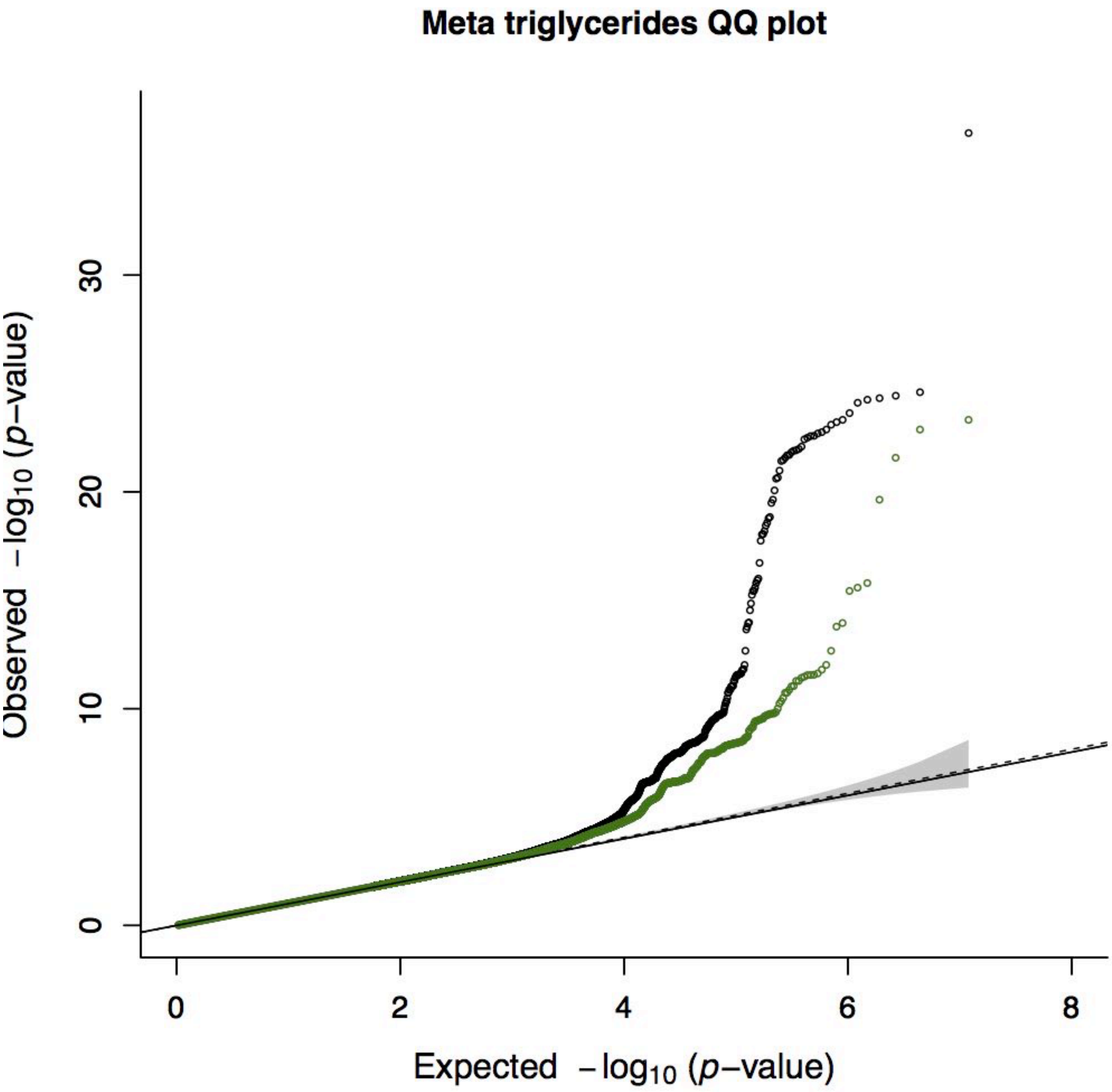

Figure S4. LocusZoom plots for top Hispanic total cholesterol meta analysis results.

Figure S4A) LocusZoom plot for meta analysis results of total cholesterol from chromosome 1, position 109717192 to 109917192.

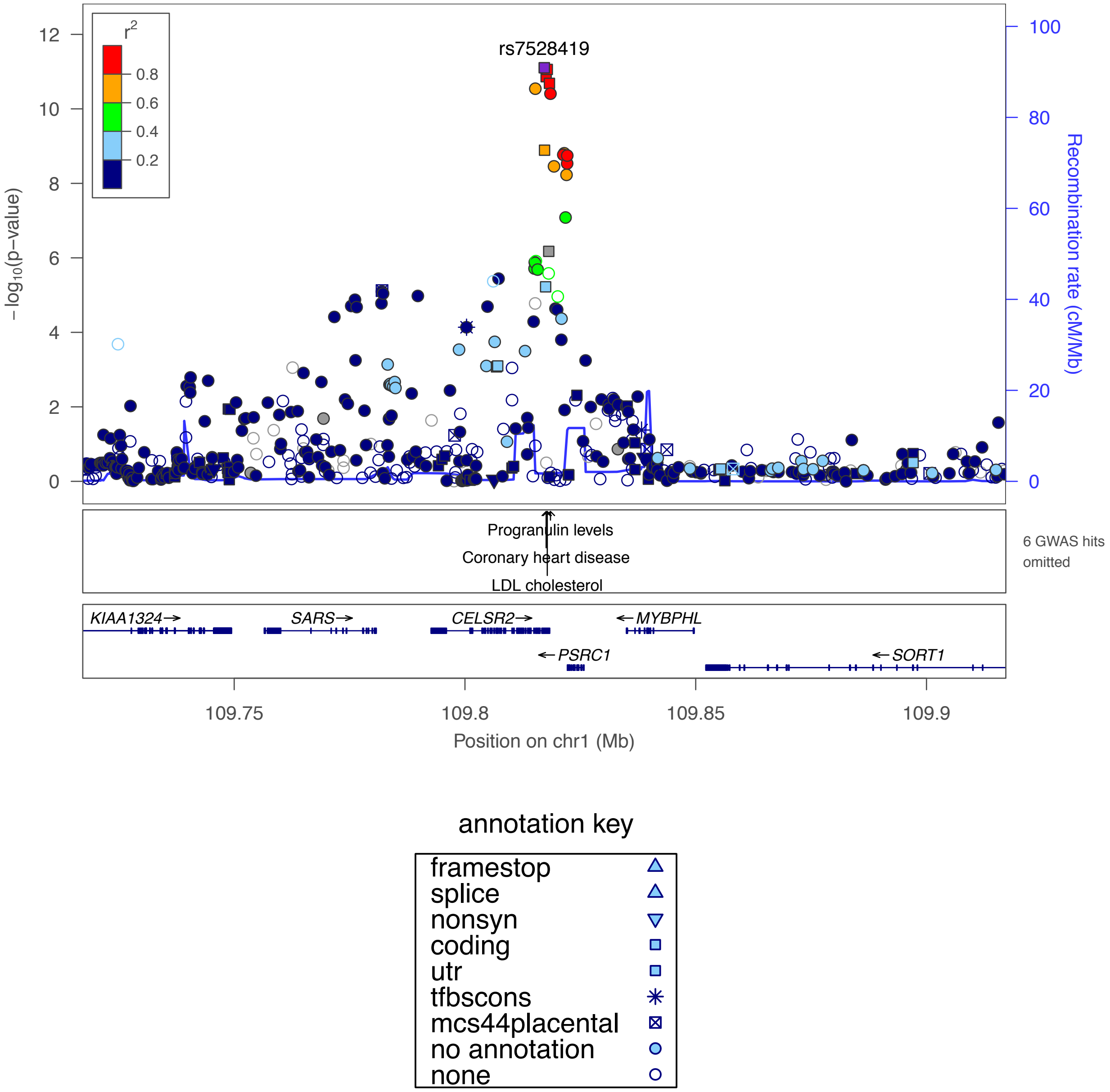

Figure S4B) LocusZoom plot for meta analysis results of total cholesterol from chromosome 11, position 116548917 to 116748917.

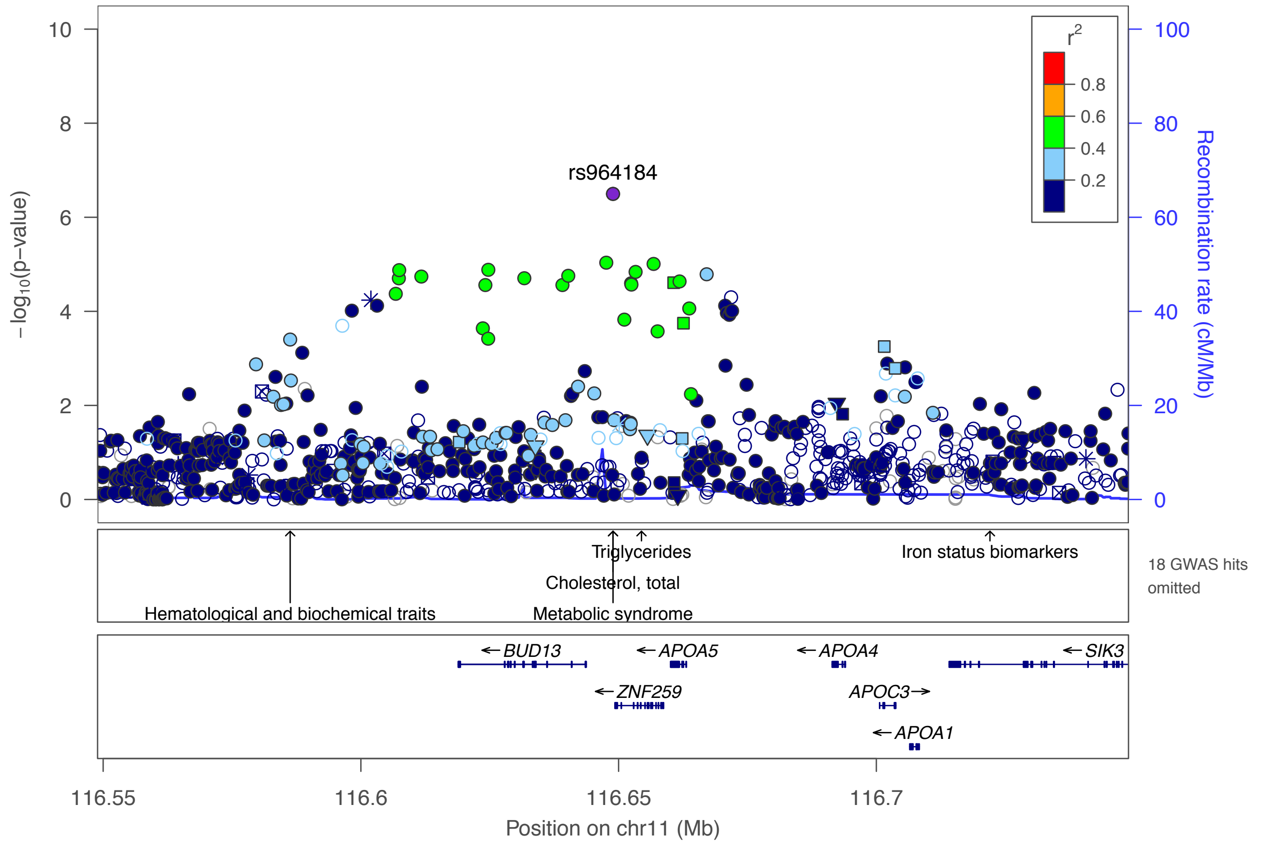

Figure S4C) LocusZoom plot for meta analysis results of total cholesterol from chromosome 19, position 11200365 to 11400365.

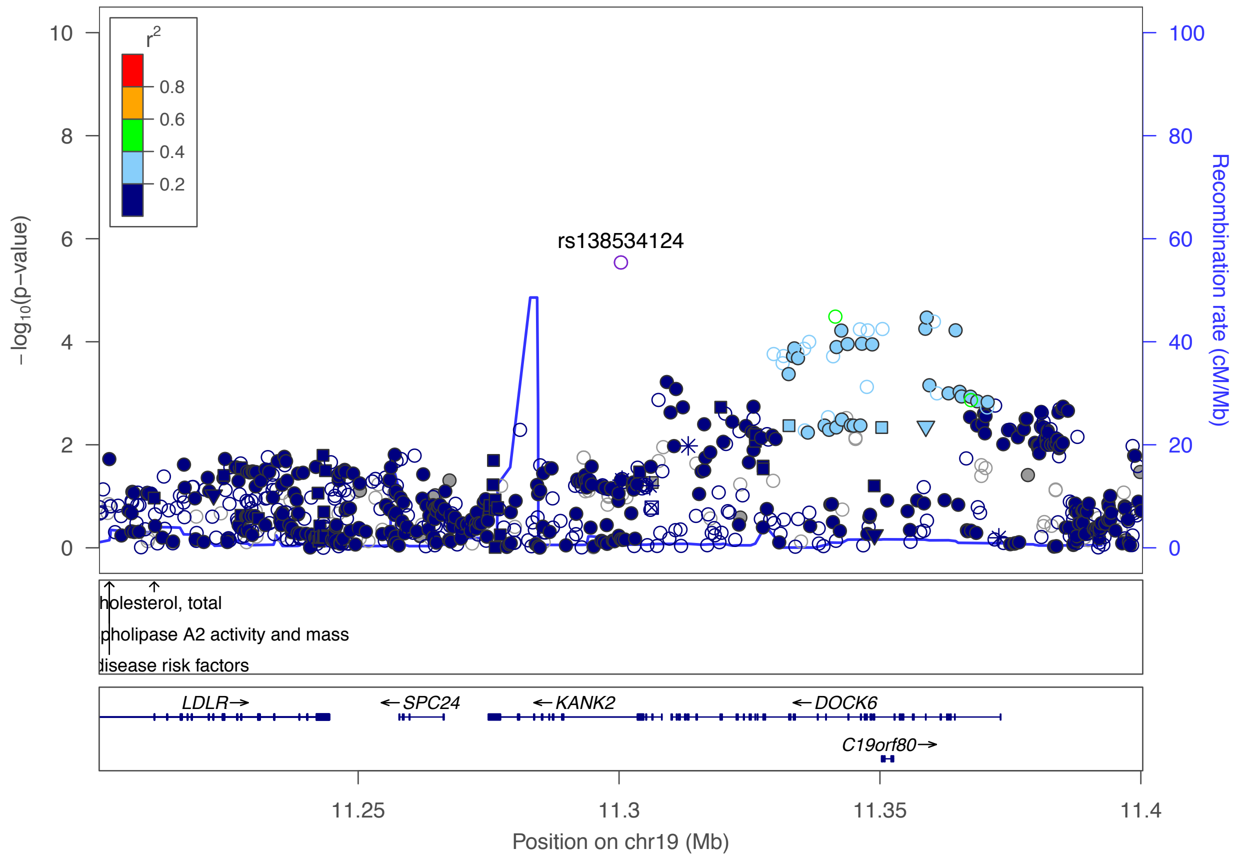

Figure S4D) LocusZoom plot for meta analysis results of total cholesterol from chromosome 19, position 19236608 to 19436608.

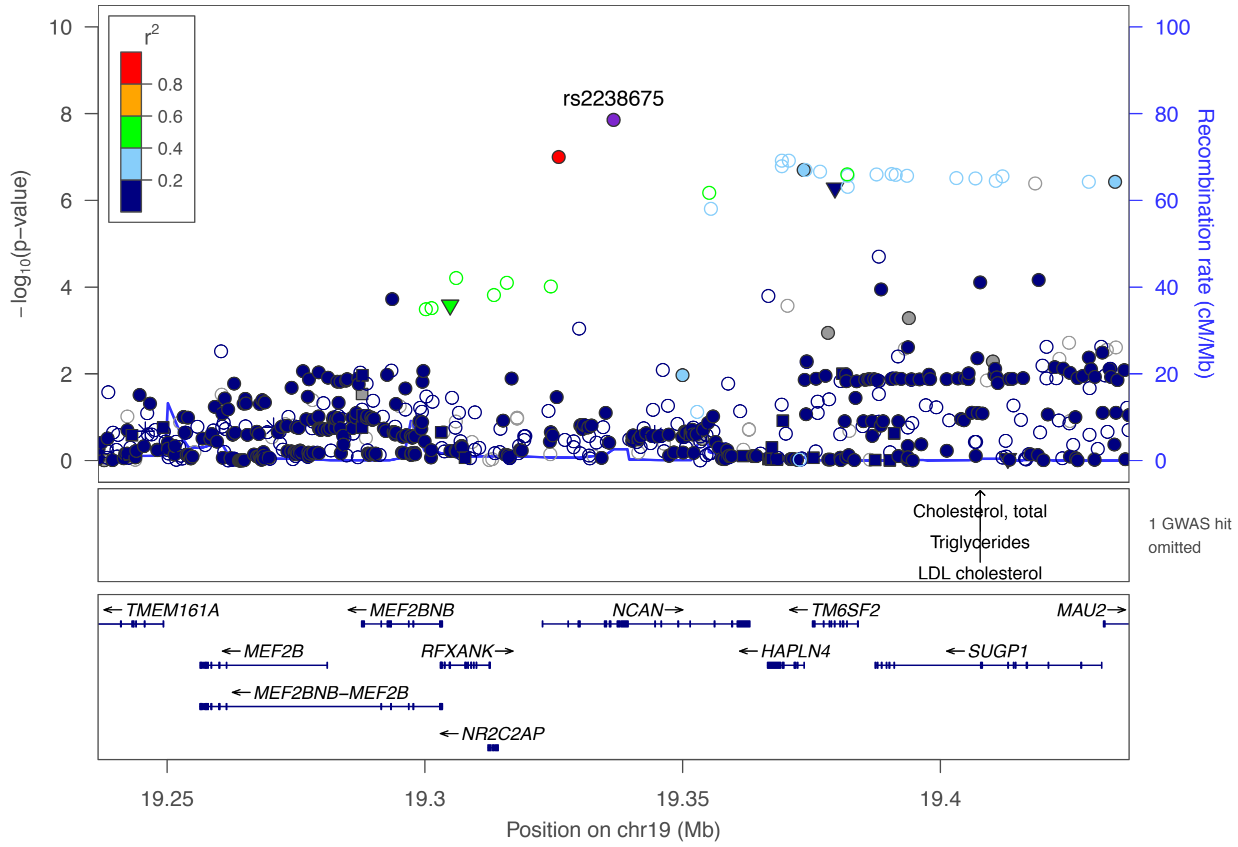

Figure S5. LocusZoom plots for top Hispanic HDL cholesterol meta analysis results.

Figure S5A) LocusZoom plot for meta analysis results of HDL cholesterol from chromosome 8, position 19791227 to 19991227.

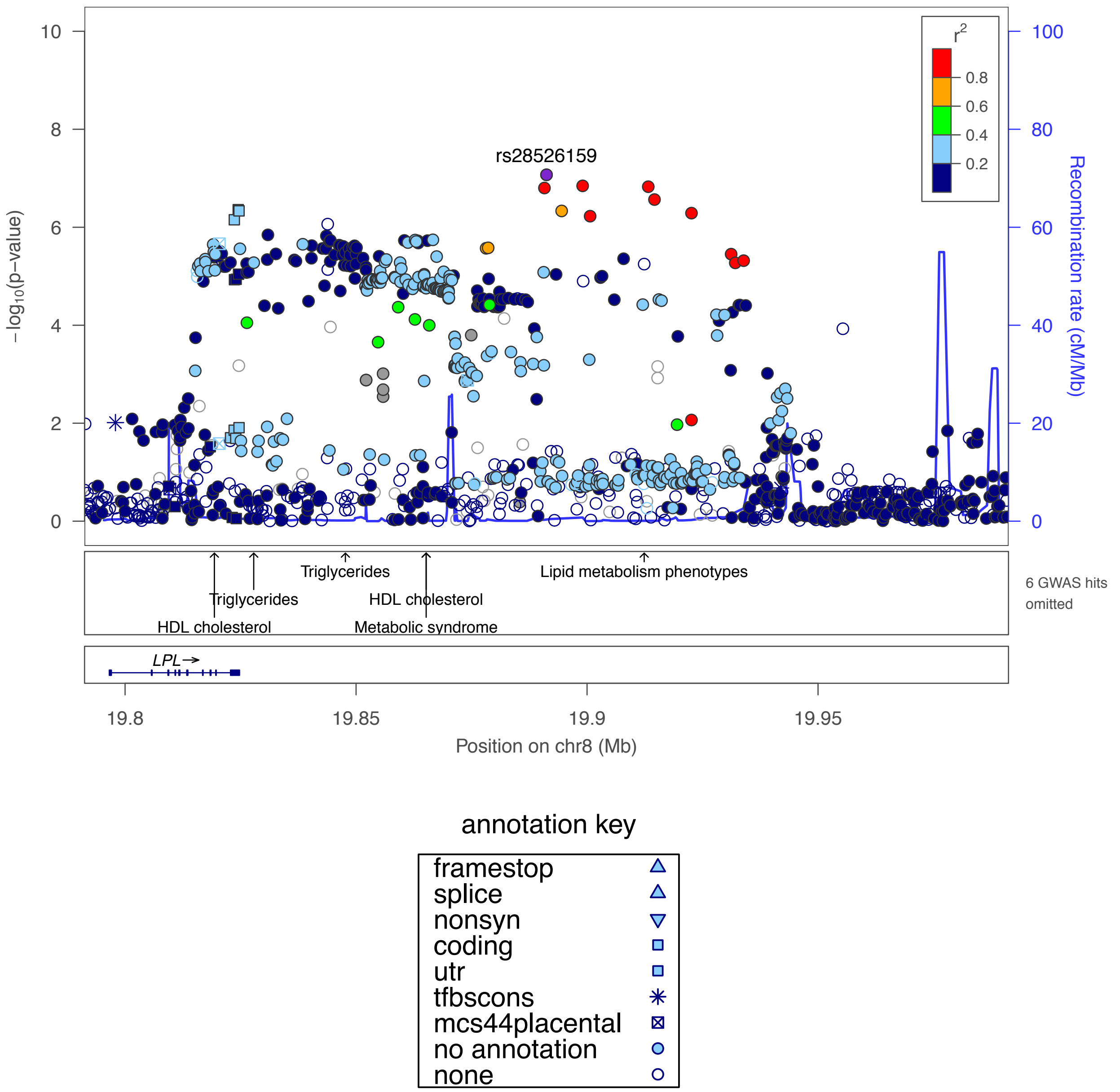

Figure S5B) LocusZoom plot for meta analysis results of HDL cholesterol from chromosome 9, position 107501541 to 107701541.

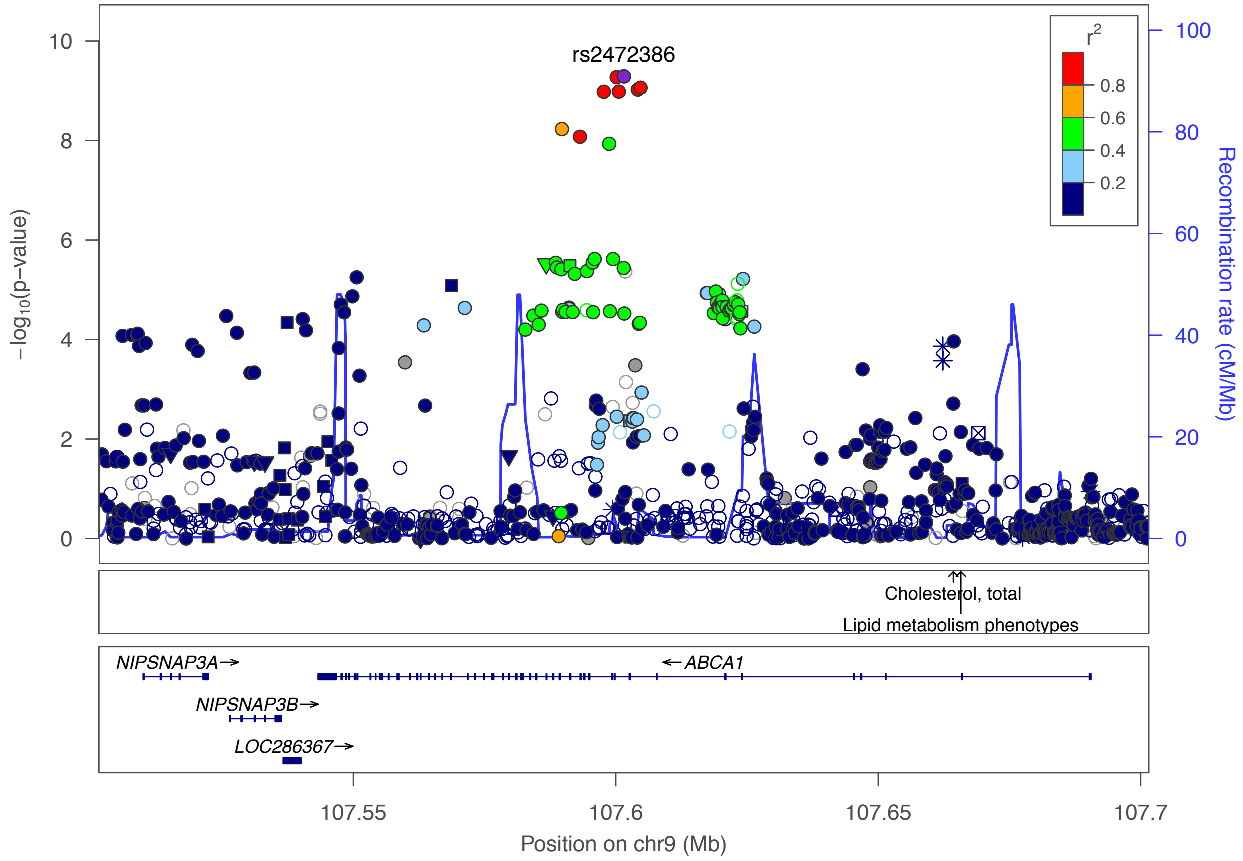

Figure S5C) LocusZoom plot for meta analysis results of HDL cholesterol from chromosome 11, position 116481641 to 116681641.

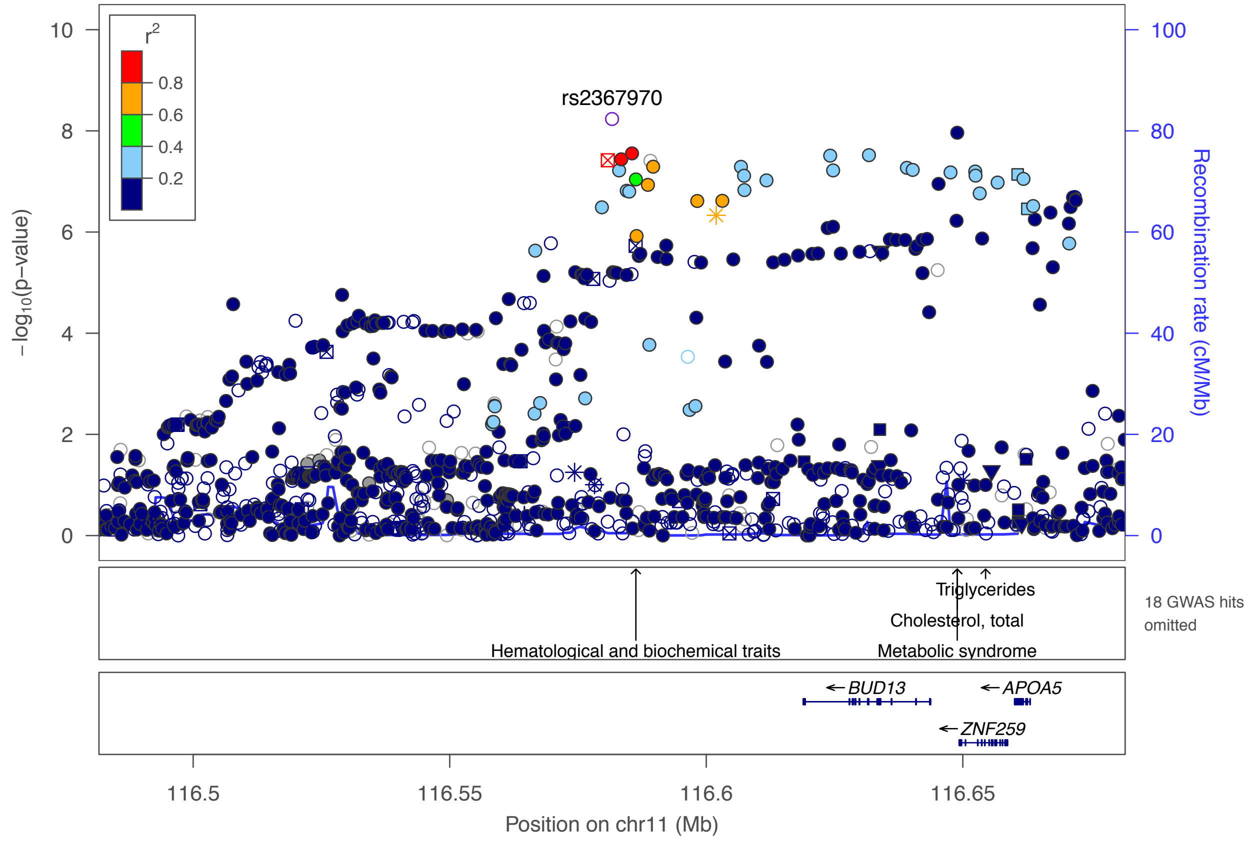

Figure S5D) LocusZoom plot for meta analysis results of HDL cholesterol from chromosome 15, position 58626744 to 58826744.

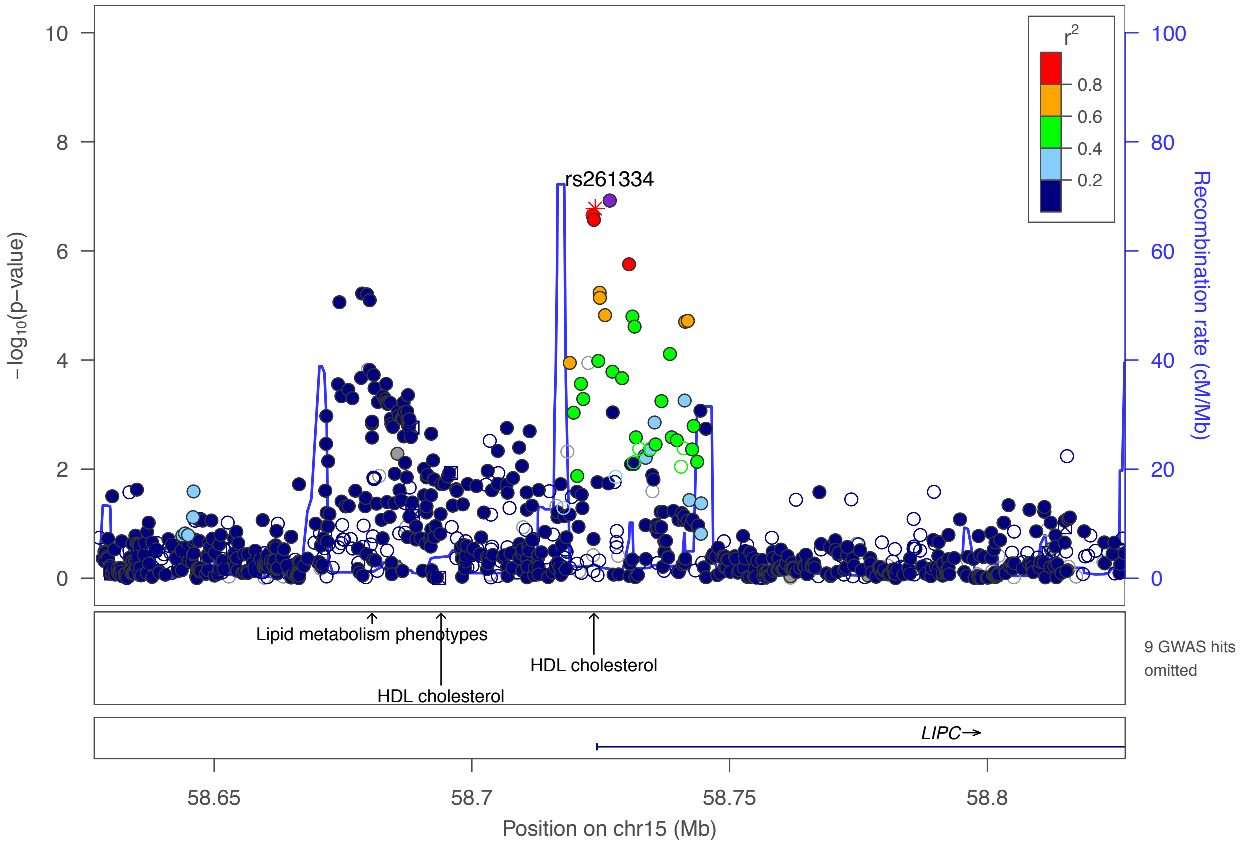

Figure S5E) LocusZoom plot for meta analysis results of HDL cholesterol from chromosome 16, position 56906590 to 57106590.

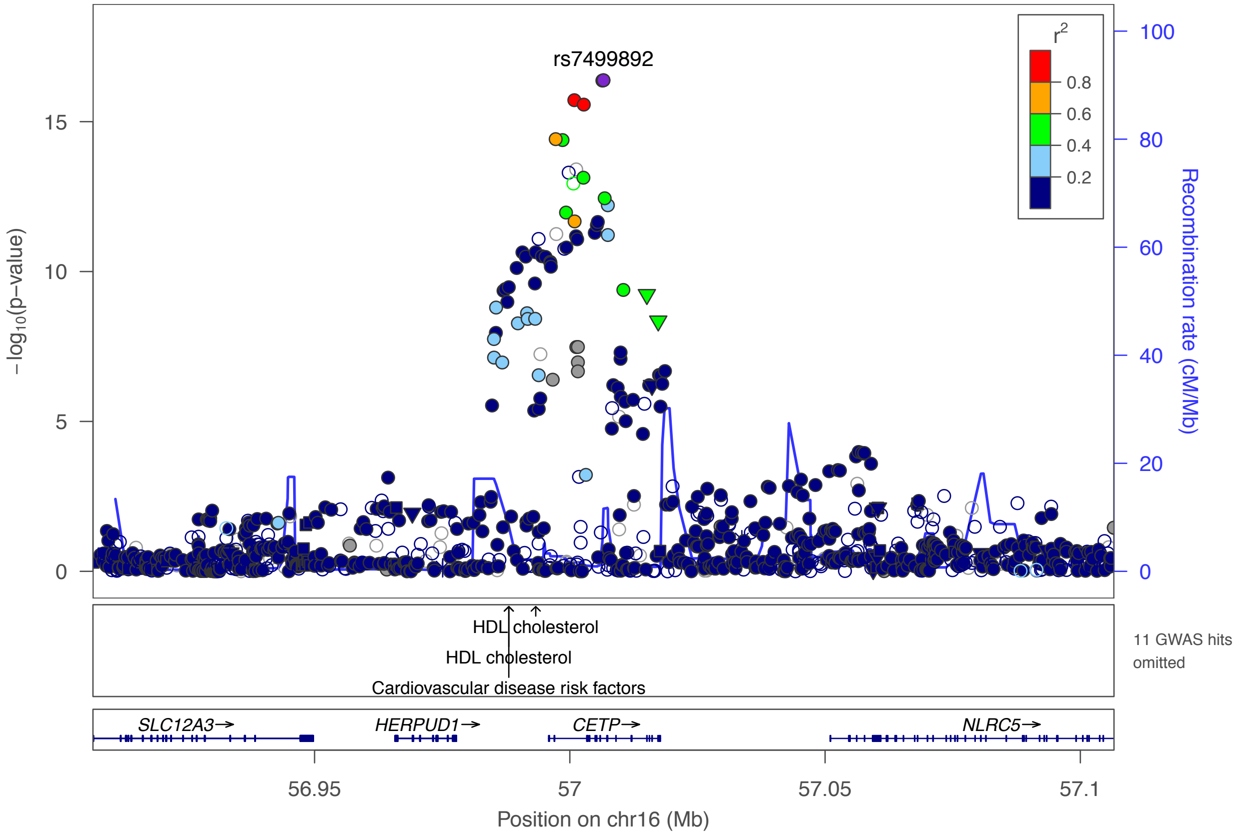

Figure S6. LocusZoom plots for top Hispanic LDL cholesterol meta analysis results.

Figure S6A) LocusZoom plot for meta analysis results of LDL cholesterol from chromosome 1, position 109717838 to 109917838.

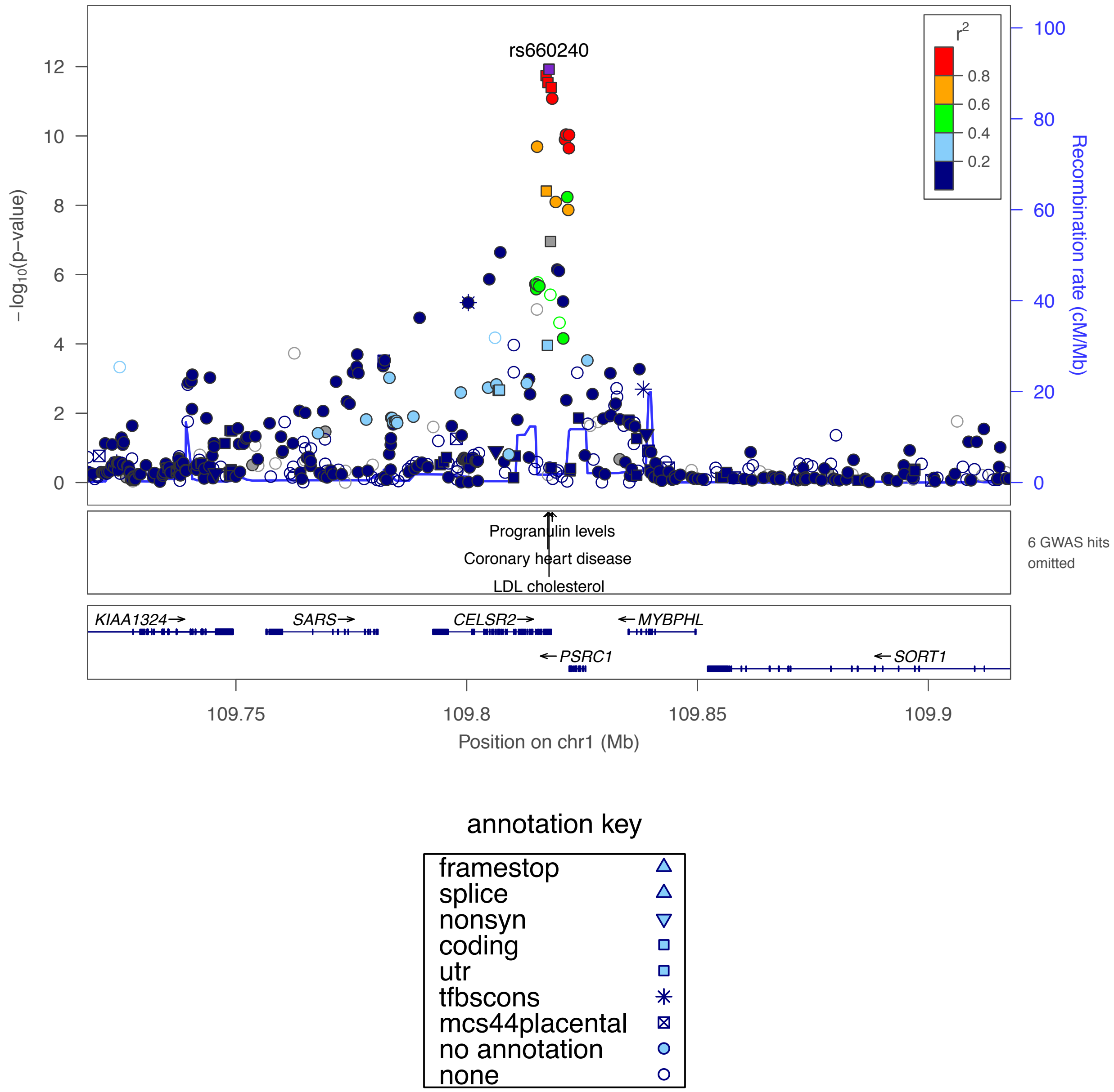

Figure S6B) LocusZoom plot for meta analysis results of LDL cholesterol from chromosome 2, position 21117490 to 21317490.

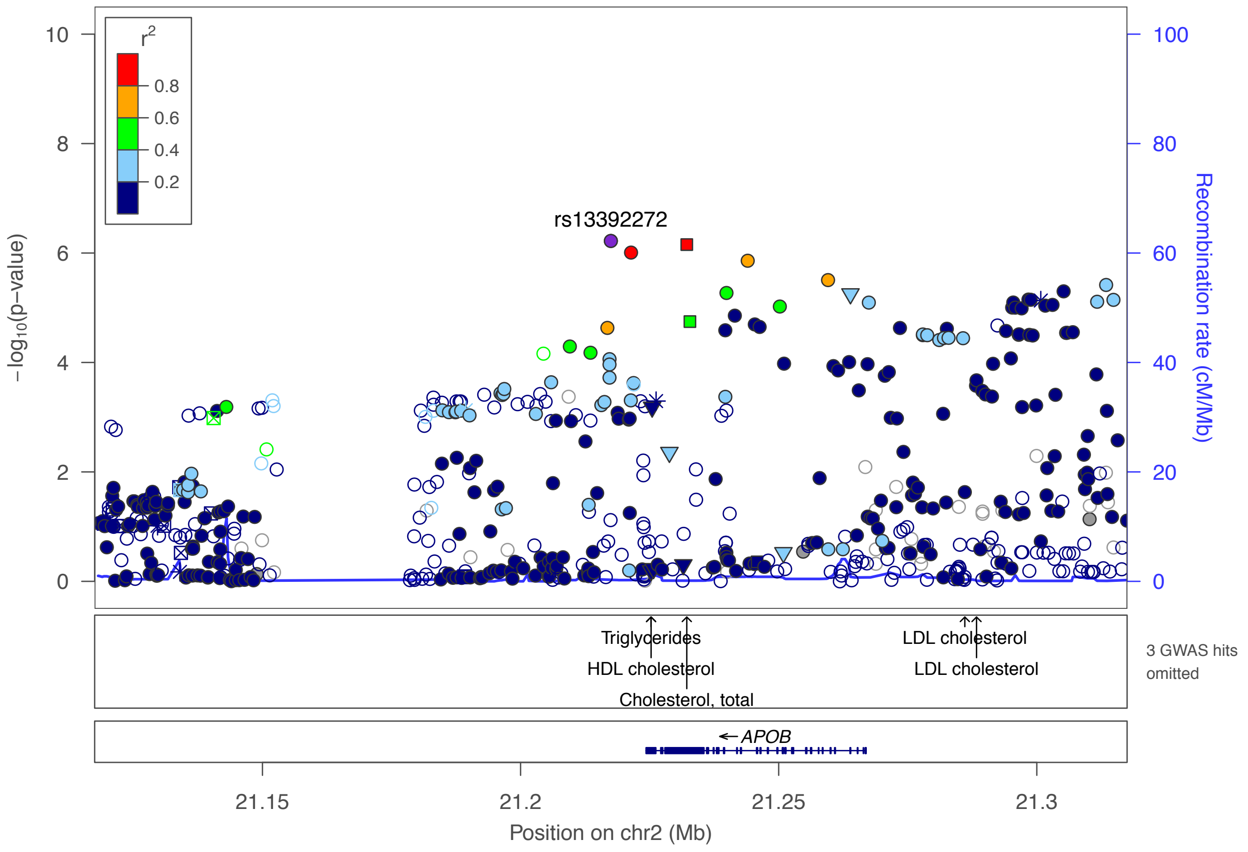

Figure S6C) LocusZoom plot for meta analysis results of LDL cholesterol from chromosome 19, position 19236608 to 19436608.

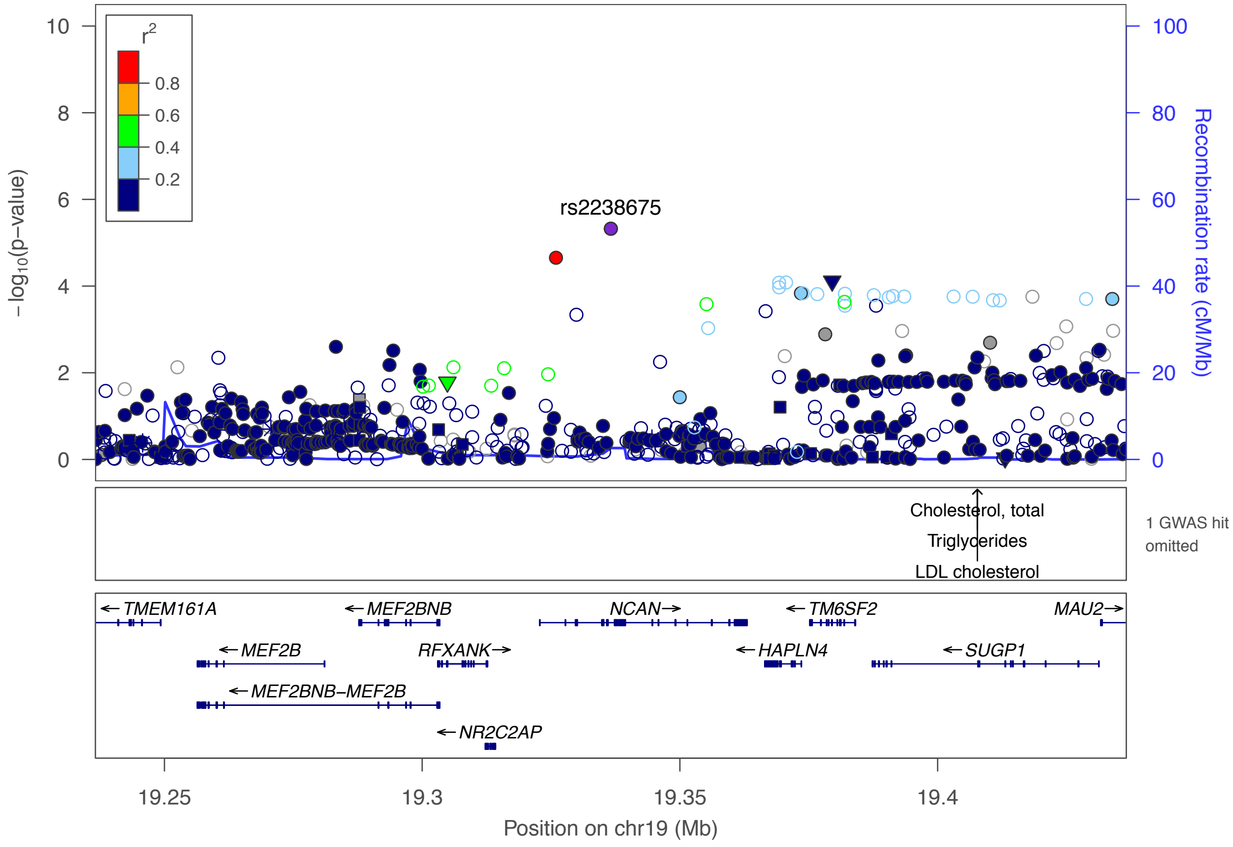

Figure S7. LocusZoom plots for top Hispanic triglycerides meta analysis results.

Figure S7A) LocusZoom plot for meta analysis results of triglycerides from chromosome 2, position 27642603 to 27842603.

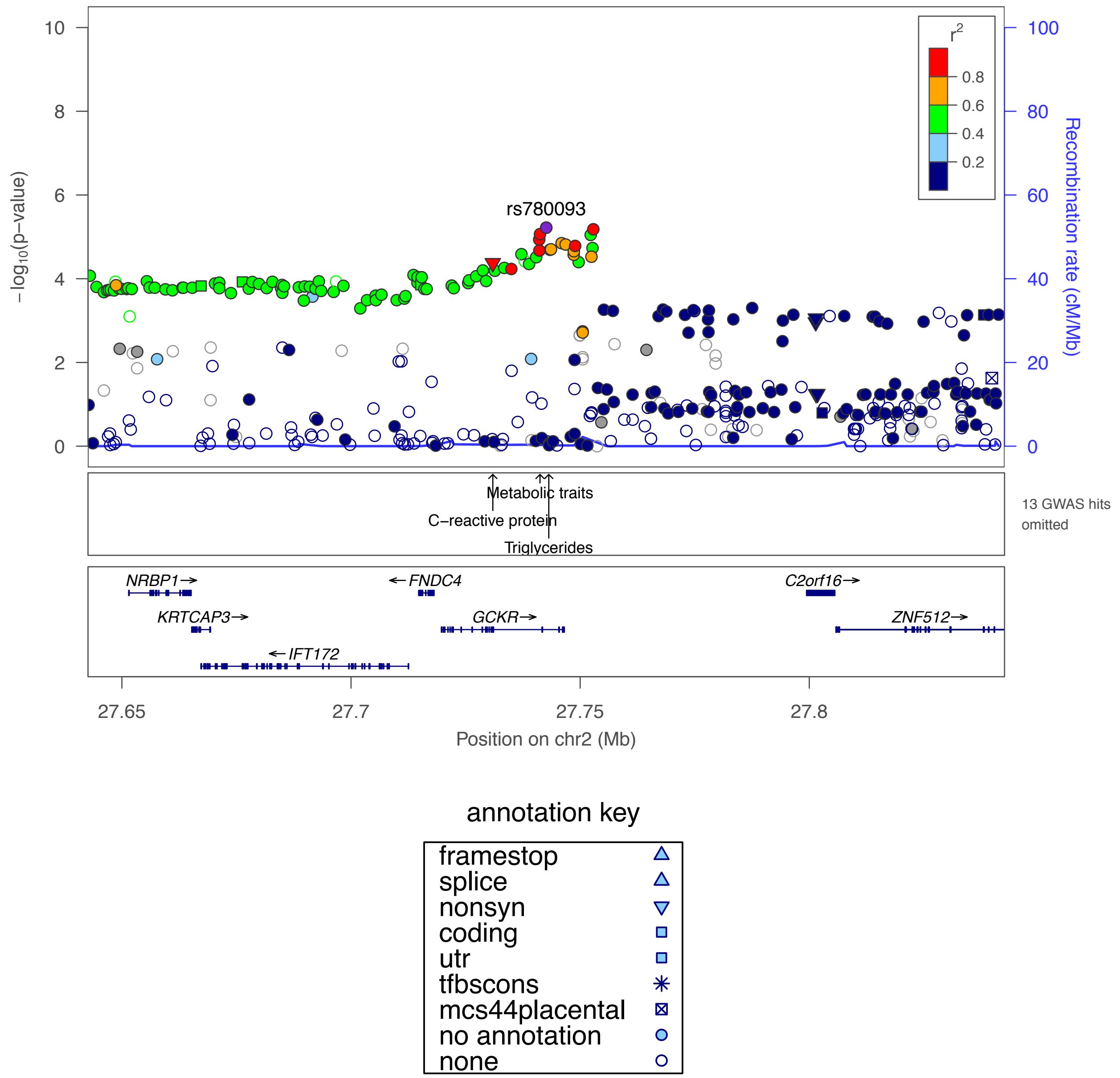

Figure S7B) LocusZoom plot for meta analysis results of triglycerides from chromosome 8, position 126391733 to 126591733.

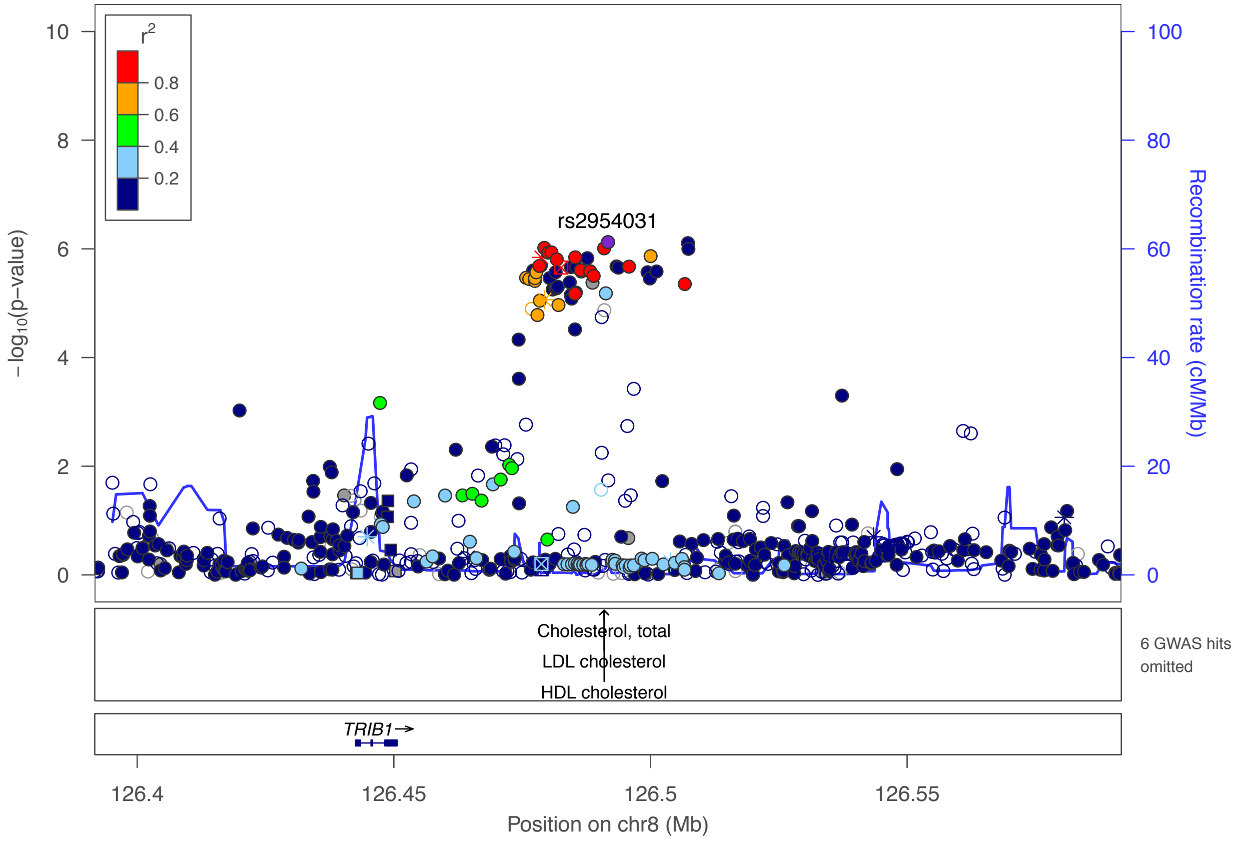

Figure S7C) LocusZoom plot for meta analysis results of triglycerides from chromosome 11, position 116548917 to 116748917.

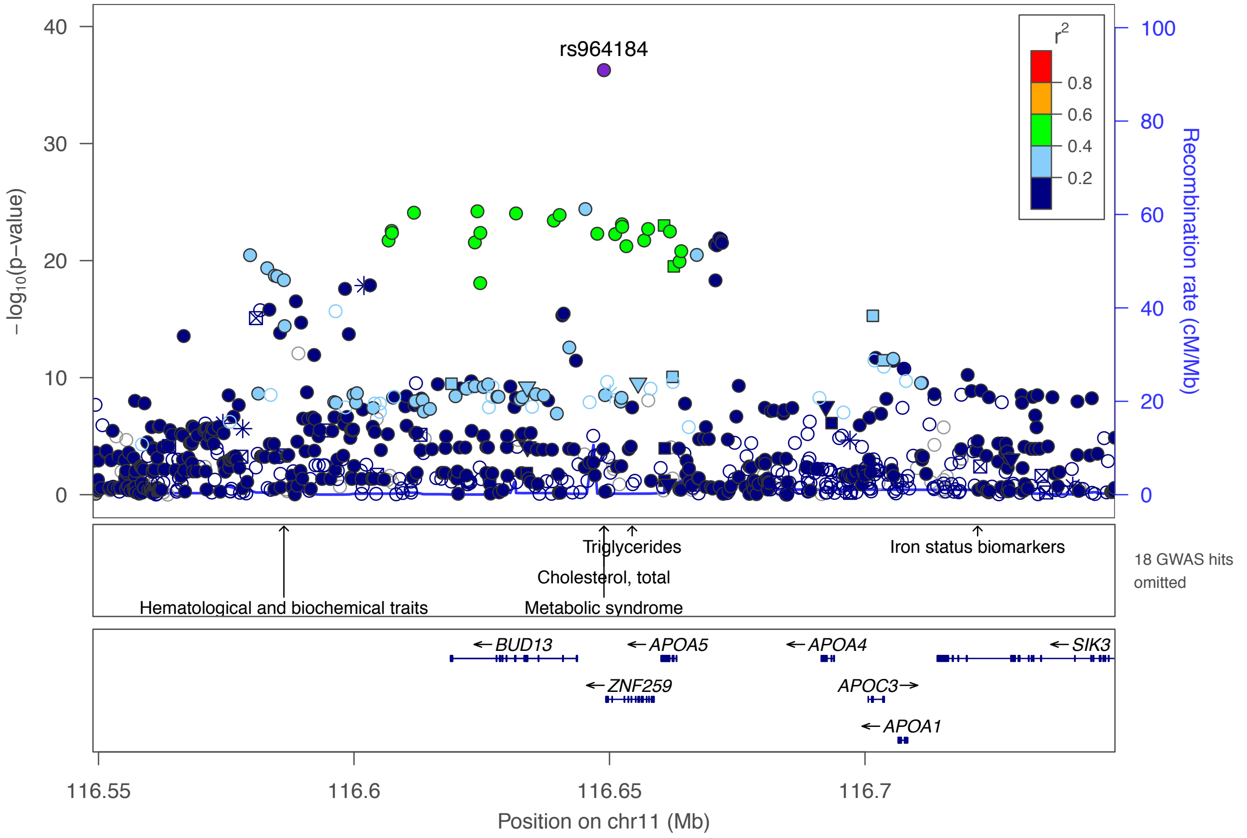

Figure S7D) LocusZoom plot for meta analysis results of triglycerides from chromosome 19, position 19355750 to 19555750.

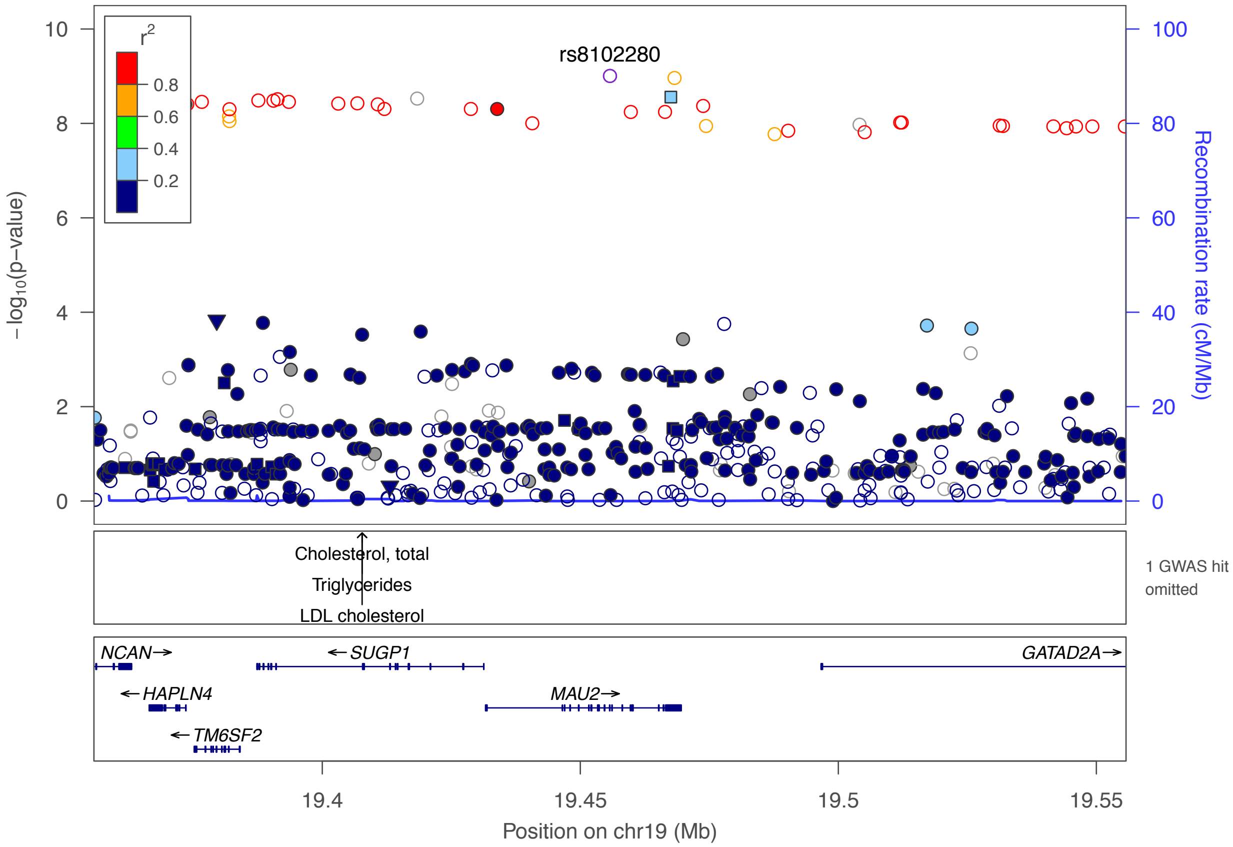

Figure S8. These figures show QQ plots for eSNPs reported in four tissues: Lymphoblastoid Cell Lines (LCL), liver, muscle and adipose tissue.

Figure S8A) QQ plots for eSNPS in LCL.

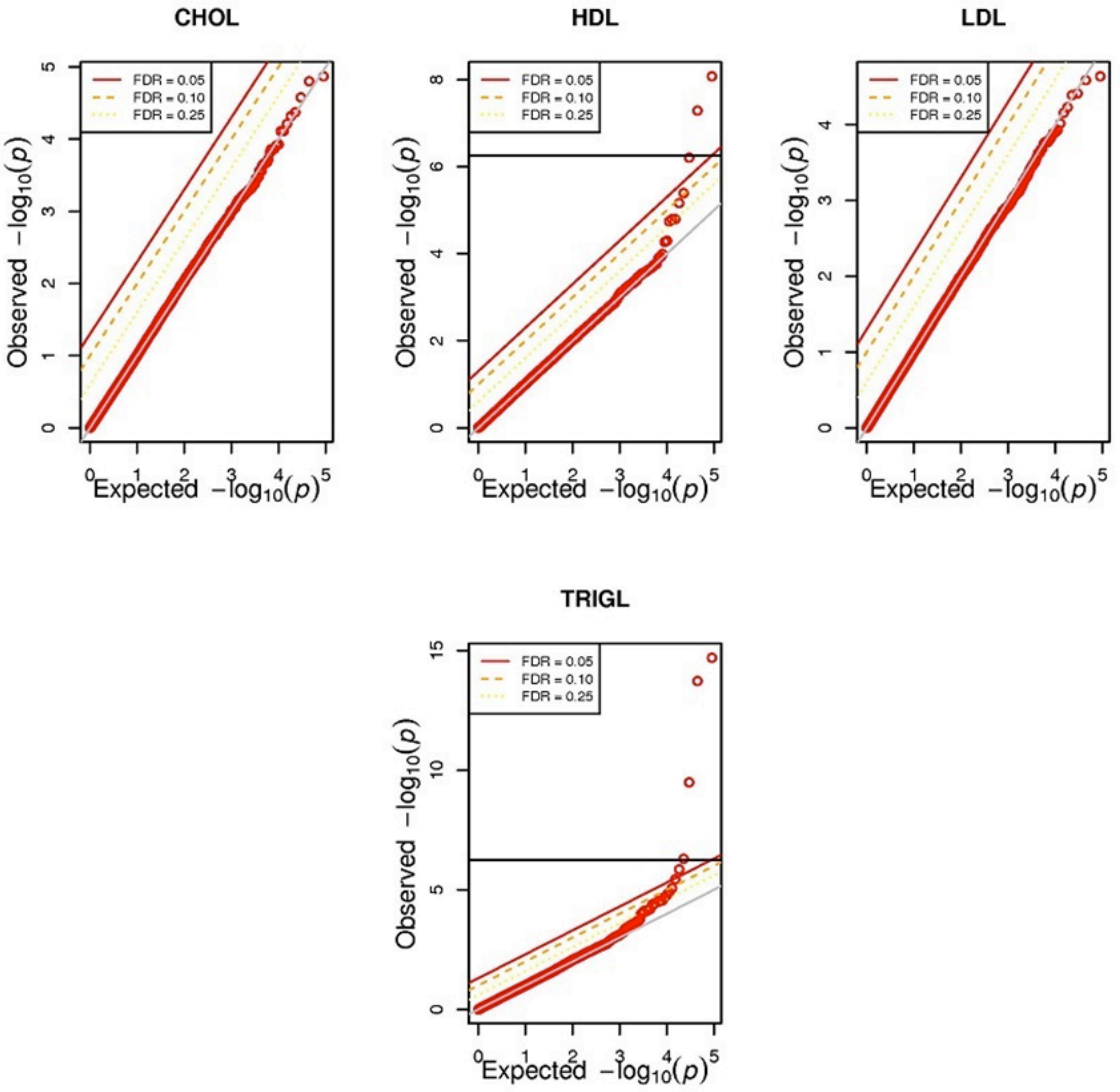

The plots show significance thresholds corresponding to False Discovery Rates of 0.05, 0.1 and 0.25. The plots also show the conventional Bonferroni-corrected threshold (black line). See Materials and Methods section for additional information.

Figure S8B) QQ plots for eSNPS in liver tissue.

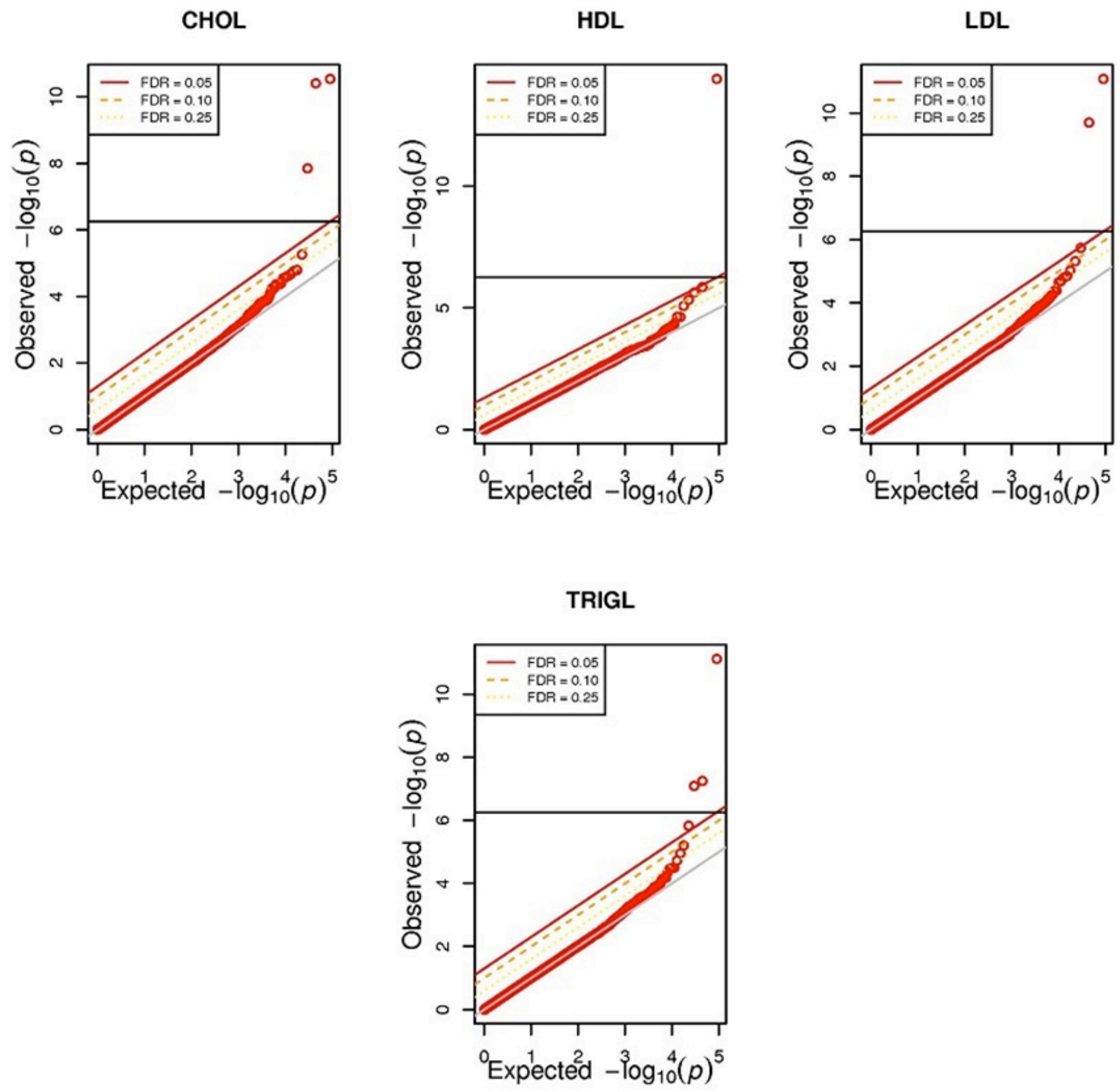

Figure S8C) QQ plots for eSNPS in muscle tissue.

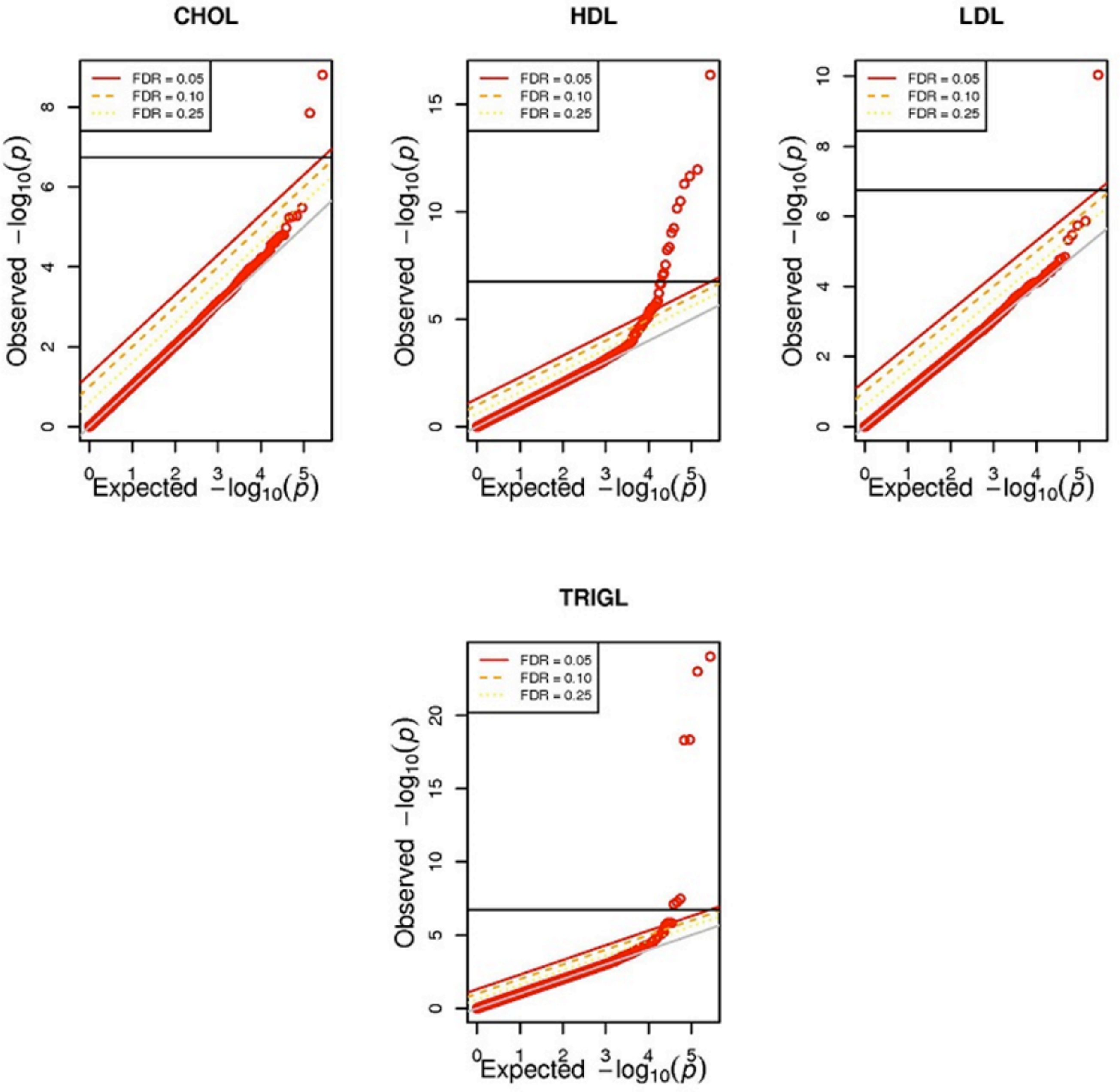

Figure S8D) QQ plots for eSNPS in adipose tissue.

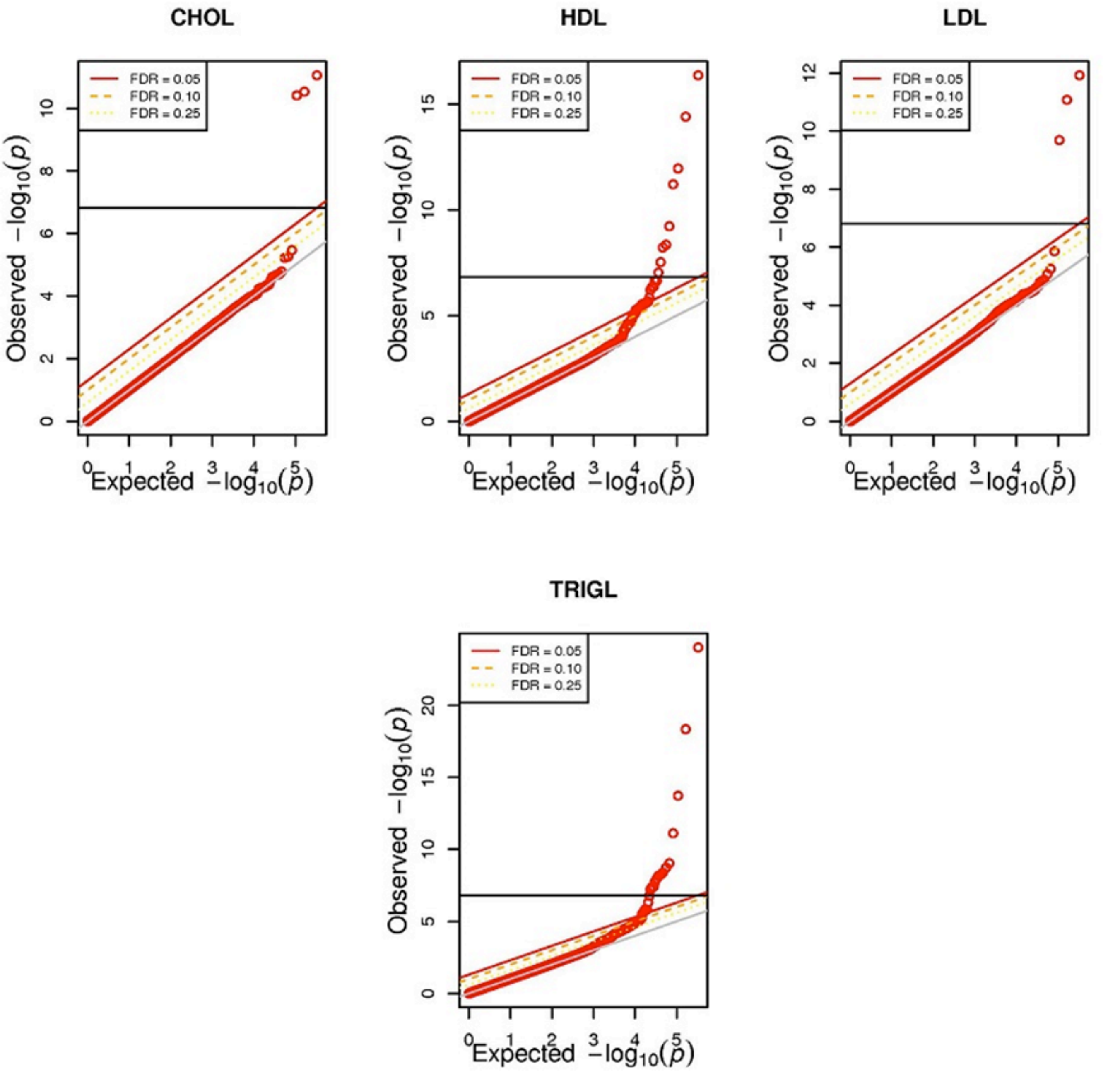

Figure S9. Correlation of estimates of effect size (beta) in the Hispanic and European meta-analyses.

Figure S9A) Correlation of estimates of effect size plot for total cholesterol.

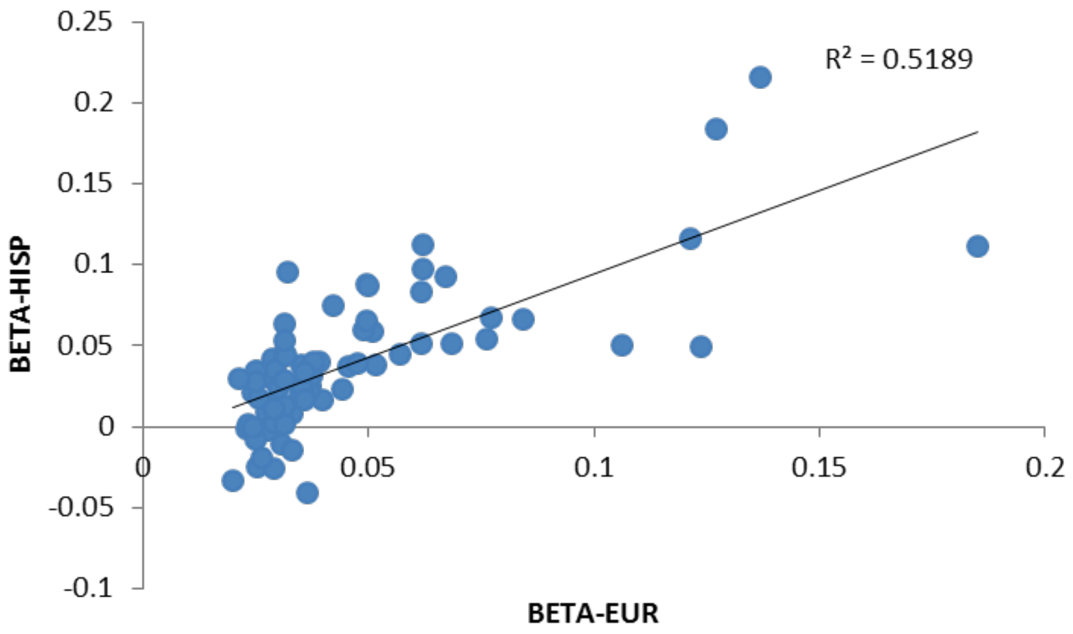

Figure S9B) Correlation of estimates of effect size plot for HDL cholesterol.

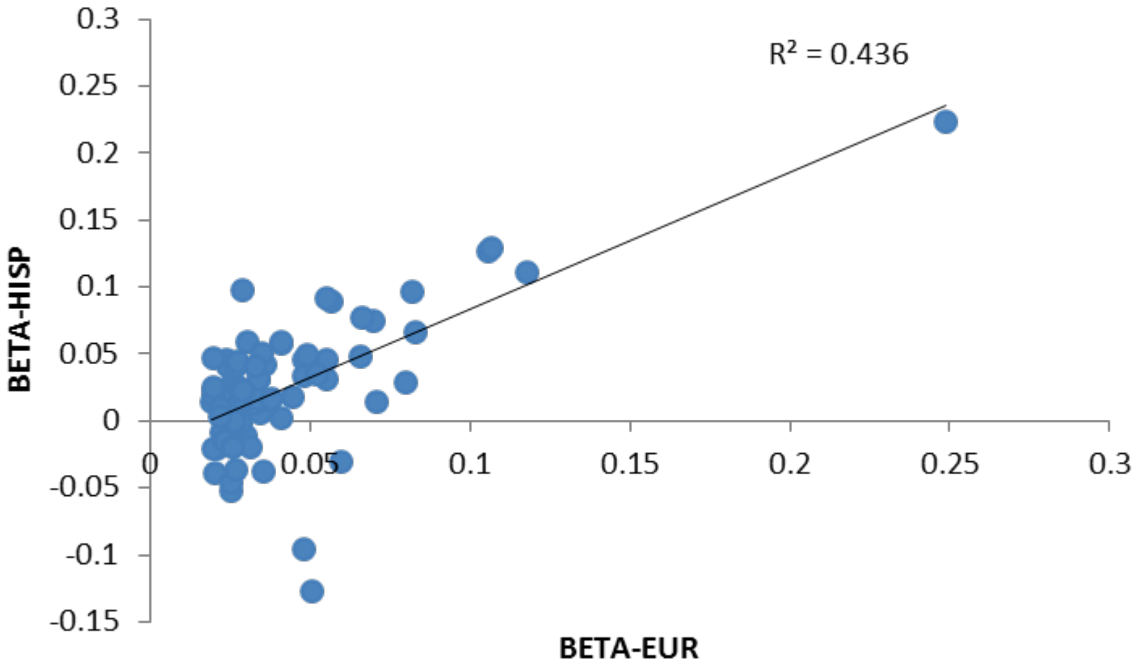

Figure S9C) Correlation of estimates of effect size plot for LDL cholesterol.

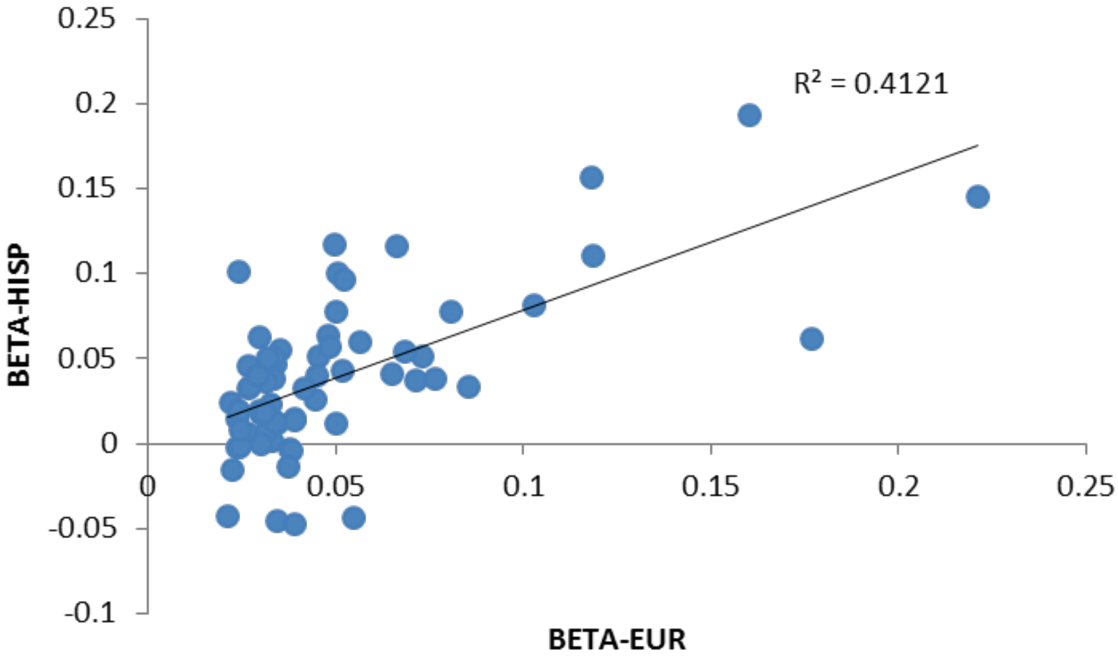

Figure S9D) Correlation of estimates of effect size plot for triglycerides

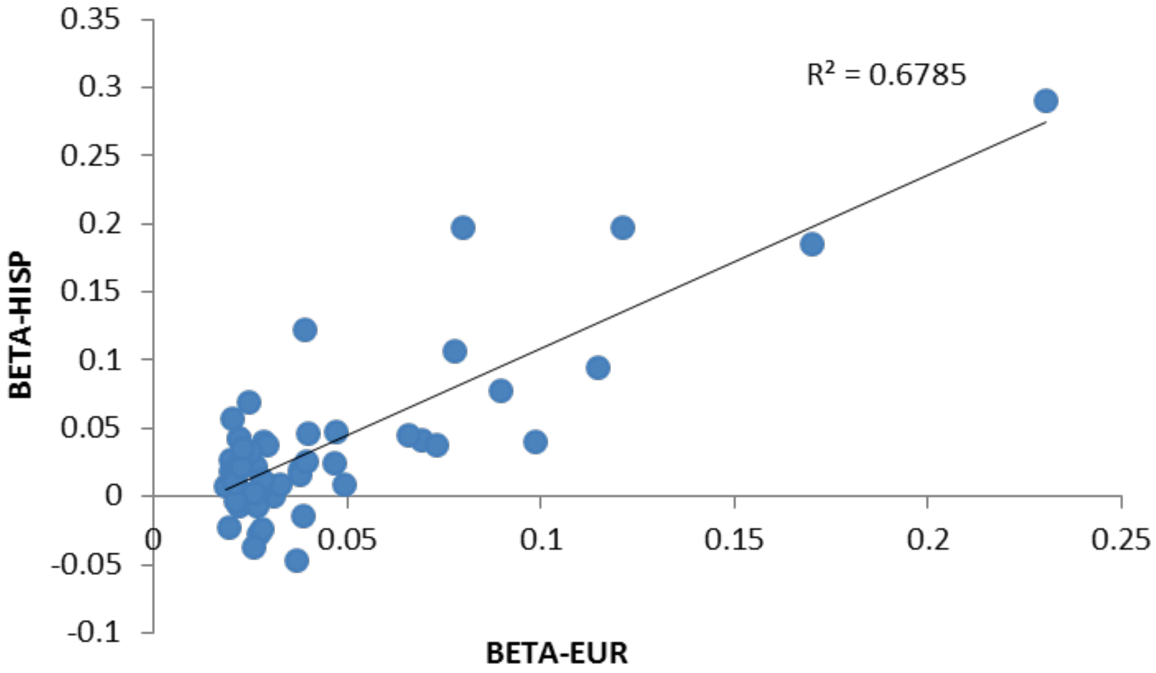

Supplemental File 1. List of genome-wide significant and suggestive markers that were followed-up in independent Hispanic samples.
NEA: Non-effect allele, EA: Effect allele, P-META: P-value of the meta-analysis, SE: Standard Error, P-HET: Heterogeneity P-value, P-SC: P-value Starr County, P-MC1: P-value Mexico City sample 1, P-MC2: P-value Mexico City sample 2, INFO-SC: Imputation info score Starr County, INFO-MC1: Imputation info score Mexico City sample 1, INFO-MC2: Imputation info score Mexico City sample 2, TCHOL: Total cholesterol, HDL: HDL cholesterol, LDL: LDL cholesterol, TRIG: Triglycerides.

GENE	CHR	POS	SNP	NEA	EA	P-META	BETA	SE	P-HET	P-SC	P-MC1	P-MC2	INFO-SC	INFO-MC1	INFO-MC2
**TCHOL**															
	1	14235500	rs10803433	G	T	3.19E-06	0.112	0.024	0.862	0.0027083	0.031137	0.0038432	0.87	0.78	1
	1	101757864	rs10875366	A	G	2.98E-06	0.112	0.024	0.620	0.049196	0.0010956	0.0040567	1	1	0.99
CELSR2	1	109817192	rs7528419	G	A	7.88E-12	0.192	0.028	0.361	6.89E-07	0.0027391	9.60E-05	0.98	0.91	0.99
LOC102724849	2	216320050	rs1250220	C	A	3.36E-06	0.105	0.023	0.173	0.11385	0.051383	1.44E-05	0.98	0.97	0.98
RPL34	4	109549678	rs3733620	G	A	2.51E-06	0.102	0.022	0.663	0.056335	0.0011534	0.0030554	0.99	0.98	0.96
	5	3162758	rs10462763	T	C	4.46E-07	0.219	0.043	0.385	0.094513	0.0008512	0.0002419	0.99	0.97	0.98
TNIP1	5	150442171	rs3792790	C	A	7.91E-06	0.101	0.023	0.839	0.044331	0.012533	0.0015385	0.98	0.97	0.95
JARID2	6	15341375	rs707833	A	G	8.93E-06	0.110	0.025	0.527	0.0057369	0.19347	0.0006294	0.98	0.87	0.96
TRIB1	8	126474306	rs2980885	A	G	1.84E-07	0.149	0.029	0.152	4.85E-06	0.07346	0.0088121	0.97	0.9	0.94
ZNF259/APOA5	11	116648917	rs964184	C	G	3.19E-07	0.116	0.023	0.435	0.023628	8.03E-05	0.0076052	0.99	0.99	0.99
	12	17800303	rs71461063	T	C	8.72E-06	0.238	0.054	0.692	0.0002765	0.026252	0.1255	0.98	0.94	0.77
	18	8847726	rs662429	C	A	2.75E-06	0.106	0.023	0.297	0.0001822	0.096665	0.0057004	0.99	0.99	0.98
KANK2/DOCK6	19	11300365	rs138534124	C	T	2.89E-06	0.161	0.034	0.699	0.020441	0.0014303	0.0078623	0.87	0.81	0.77
NCAN	19	19336608	rs2238675	T	C	1.40E-08	0.197	0.035	0.579	0.061159	0.0002087	6.23E-05	0.81	0.85	0.91
**HDL**															
	2	21578468	rs219558	T	G	2.82E-06	0.115	0.024	0.630	0.0011899	0.0096394	0.017465	1	0.84	0.99
LPL	8	19891227	rs28526159	C	T	8.49E-08	0.116	0.022	0.709	0.0010805	0.030115	0.0001838	0.99	0.99	0.98
ABCA1	9	107601541	rs2472386	G	A	5.13E-10	0.140	0.023	0.651	0.0013329	0.0091421	2.22E-06	0.92	0.9	0.99
	10	107061270	rs2930450	G	T	7.75E-06	0.203	0.045	0.493	0.0006364	0.014827	0.050934	0.92	0.87	0.8
ZNF259/APOA5	11	116581641	rs2367970	A	G	5.82E-09	0.149	0.026	0.156	0.052695	0.013147	1.41E-07	0.94	0.91	0.97
NOS1	12	117779856	rs56257650	C	A	4.01E-06	0.112	0.024	0.072	0.0002104	0.0006753	0.2648	0.98	0.88	0.82
LIPC	15	58726744	rs261334	C	G	1.19E-07	0.116	0.022	0.435	0.000963	0.09369	6.35E-05	0.97	0.92	0.99
	15	60351885	rs60314318	C	T	2.35E-06	0.162	0.034	0.341	0.0037603	0.1819	0.0001598	0.96	0.83	0.94
LOC102467079	16	52130521	rs11646391	C	G	3.57E-06	0.244	0.053	0.330	6.19E-05	0.0231	0.11443	0.99	0.98	0.88
CETP	16	57006590	rs7499892	T	C	4.16E-17	0.233	0.028	0.443	0.0005266	6.42E-05	2.77E-11	0.86	0.75	0.99
ZNF607	19	38198540	rs62108344	T	C	7.37E-06	0.137	0.031	0.628	0.0006032	0.050706	0.019649	0.97	0.96	0.96
**LDL**															
ST6GALNAC3	1	76676058	rs12064344	T	C	1.06E-06	0.218	0.045	0.966	0.0040446	0.011268	0.0024285	1	1	0.97
CELSR2	1	109817838	rs660240	T	C	1.19E-12	0.203	0.029	0.551	2.89E-06	0.000153	8.35E-05	0.99	0.91	0.98
APOB	2	21217490	rs13392272	C	T	6.01E-07	0.113	0.023	0.075	0.0005564	8.56E-05	0.09813	1	0.97	0.98
THSD7B	2	138315429	rs140546204	G	A	7.10E-06	0.147	0.033	0.880	0.016973	0.0033889	0.013233	0.97	0.96	0.94
TPRG1	3	188864748	rs76543661	T	C	5.67E-06	0.288	0.063	0.719	0.0016955	0.096064	0.0032955	0.92	0.81	0.76
	5	3172052	rs13190086	C	T	2.53E-06	0.172	0.036	0.632	0.090871	0.0015254	0.0014386	1	0.98	0.99
LINGO2	9	28018301	rs13295840	G	T	9.93E-06	0.113	0.026	0.278	0.0003681	0.0058366	0.17954	0.98	0.84	0.72
ITGBL1	13	102179256	rs9585723	T	G	9.44E-06	0.195	0.044	0.681	0.019526	0.035179	0.0011936	0.97	0.96	0.96
ZNF771	16	30423751	rs4232893	A	G	1.24E-06	0.208	0.043	0.161	0.064316	0.083104	5.24E-06	1	0.75	0.99
DOK6	18	67284055	rs11662540	G	T	1.47E-06	0.115	0.024	0.473	0.0002261	0.034017	0.010295	0.98	0.93	0.95
NCAN	19	19336608	rs2238675	T	C	4.75E-06	0.161	0.035	0.877	0.077709	0.0045498	0.001534	0.81	0.85	0.91
**TRIG**															
GCKR	2	27742603	rs780093	C	T	6.03E-06	0.103	0.023	0.914	0.0039874	0.023052	0.0060867	0.98	0.98	0.99
	3	165650549	rs4419424	A	G	7.36E-06	0.101	0.023	0.887	0.0072204	0.0060448	0.015482	0.99	0.99	0.99
	4	32616342	rs13136088	A	G	8.86E-06	0.172	0.039	0.486	0.021562	0.079151	0.0003071	1	0.94	0.95
CAMK2D	4	114557476	rs2189367	G	C	8.16E-06	0.104	0.023	0.764	0.038441	0.015115	0.0012496	0.99	0.99	0.99
RAI14	5	34752228	rs17593349	T	C	6.62E-06	0.147	0.033	0.603	0.0040059	0.091535	0.001194	0.96	0.93	0.95
ACTBP2/AP3B1	5	77631634	rs4588572	G	A	4.80E-06	0.099	0.022	0.645	0.0008363	0.045337	0.0078954	0.97	0.94	0.95
UIMC1	5	176395190	rs353503	G	T	8.96E-06	0.100	0.022	0.624	0.035196	0.0013081	0.013767	0.99	0.99	0.99
	7	91101465	rs4131680	G	A	5.95E-06	0.114	0.025	0.688	0.0008257	0.10045	0.0052147	0.99	0.92	1
TRIB1	8	126491733	rs2954031	T	G	7.51E-07	0.112	0.023	0.843	0.023227	0.0051862	0.000517	0.99	0.96	0.99
	9	18380820	rs79186818	G	A	5.93E-06	0.211	0.047	0.532	9.34E-05	0.031746	0.11536	0.97	0.95	0.88
	9	26500798	rs62542129	T	C	6.63E-06	0.098	0.022	0.035	0.0032918	2.70E-05	0.30235	0.99	0.99	0.97
ZNF259/APOA5	11	116648917	rs964184	C	G	5.32E-37	0.289	0.023	0.600	6.12E-09	1.25E-15	4.44E-16	0.99	0.99	0.99
	16	57387682	rs34993999	C	A	7.70E-06	0.206	0.046	0.515	0.0008375	0.22026	0.0025456	0.9	0.82	0.95
	18	44337708	rs3809959	G	C	6.46E-06	0.102	0.023	0.600	0.0009764	0.075555	0.00535	0.99	0.99	0.98
MAU2	19	19455750	rs8102280	A	G	9.86E-10	0.251	0.041	0.492	0.21368	3.92E-05	6.37E-06	0.89	0.92	0.99

Supplemental File 2. Conditional analyses for regions in which different SNPs were associated with different lipid traits in the Hispanic meta-analysis.
The conditioning SNP was the lead SNP for each lipid trait. The tested SNP is the lead SNP in the same region for an alternative lipid trait (indicated in parenthesis)

		**Original p-values of conditioning and tested SNPs**				**Results conditional analysis**		
GENE	**CHR**	**Conditioning SNP**	**P-value conditioning SNP**	**Tested SNP**	**P-value tested SNP**	**P-value**	**BETA**	**SE**
**TCHOL**								
CELSR2	1	rs7528419	7.88307E-12	rs660240 (lead SNP for LDL)	8.73E-12	0.880503	0.004208	0.027998
TRIB1	8	rs2980885	1.84444E-07	rs2954031 (lead SNP for TRIG)	3.45E-05	0.02032	-0.052256	0.022516
ZNF259/APOA5	11	rs964184	3.18508E-07	rs2367970 (lead SNP for HDL)	0.00591623	0.838687	0.005182	0.025458
NCAN	19	rs2238675	1.39521E-08	rs8102280 (lead SNP for TRIG)	1.73E-06	0.608329	-0.020732	0.040453
**HDL**								
ZNF259/APOA5	11	rs2367970	5.82003E-09	rs964184 (lead SNP for TCHOL and TRIG)	1.09E-08	0.003767	0.065491	0.022598
**LDL**								
CELSR2	1	rs660240	1.18694E-12	rs7528419 (lead SNP for TCHOL)	1.81E-12	0.897669	-0.00364	0.028312
NCAN	19	rs2238675	4.74612E-06	rs8102280 (lead SNP for TRIG)	0.000514251	0.927602	-0.003708	0.040819
**TRIG**								
TRIB1	8	rs2954031	7.51468E-07	rs2980885 (lead SNP for TCHOL)	4.68E-05	0.01857	-0.067289	0.028579
ZNF259/APOA5	11	rs964184	5.32042E-37	rs2367970 (lead SNP for HDL)	1.72E-16	0.050106	0.049113	0.025068
MAU2	19	rs8102280	9.86263E-10	rs2238675 (lead SNP for TCHOL and LDL)	5.73E-08	0.311588	-0.035131	0.034719

Supplemental File 3. Follow-up of the genome-wide and suggestive signals observed in this study in three independent Hispanic samples (WHI, MESA and BioMe). For the individual studies, we report the p-values and the effect allele frequencies. For the meta-analysis, we report the p-values, beta coefficients and standard errors, heterogeneity p-value, and direction of effect. Note: For the discovery sample, we show the allele frequency observed in the largest sample (Mexico City 2)

GENE	CHR	POS	LEAD SNP	NEA	EA	P-THIS STUDY (N=4,715)	EAF MC2	P-WHI (N=3,587)	EAF WHI	P-MESA (N=2,127)	EAF MESA	P-BioMe (N=2,162)	EAF BioMe	P-META (N=12,591)	BETA	SE	P-HET	EFFECTS
TCHOL																		
	1	14235500	rs10803433	G	T	3.19E-06	0.307	0.377	0.407	0.960	0.419	0.490	0.463	0.046	0.029	0.014	2.82E-04	+---
	1	101757864	rs10875366	A	G	2.98E-06	0.730	0.446	0.696	0.558	0.720	0.776	0.743	0.006	0.039	0.014	0.002	+-++
CELSR2	1	109817192	rs7528419	G	A	7.88E-12	0.823	3.01E-06	0.792	3.81E-12	0.777	0.003	0.764	9.05E-27	0.172	0.016	0.013	++++
LOC102724849	2	216320050	rs1250220	C	A	3.36E-06	0.683	0.022	0.605	0.557	0.591	0.417	0.544	4.12E-05	0.055	0.013	0.012	++-+
RPL34	4	109549678	rs3733620	G	A	2.51E-06	0.466	0.079	0.662	0.399	0.355	0.401	0.778	0.042	0.028	0.014	5.27E-05	+--+
	5	3162758	rs10462763	T	C	4.46E-07	0.071	0.945	0.055	0.637	0.057	0.228	0.048	5.29E-04	0.097	0.028	0.002	+--+
TNIP1	5	150442171	rs3792790	C	A	7.91E-06	0.627	0.482	0.572	0.101	0.592	0.378	0.575	5.17E-05	0.053	0.013	0.057	++++
JARID2	6	15341375	rs707833	A	G	8.93E-06	0.290	0.477	0.320	0.270	0.326	0.502	0.363	0.002	0.044	0.014	0.006	+++-
TRIB1	8	126474306	rs2980885	A	G	1.84E-07	0.172	0.833	0.216	0.166	0.198	0.260	0.191	2.70E-05	0.070	0.017	0.005	++++
ZNF259/APOA5	11	116648917	rs964184	C	G	3.19E-07	0.367	5.50E-05	0.248	0.055	0.292	0.005	0.216	4.88E-13	0.103	0.014	0.674	++++
	12	17800303	rs71461063	T	C	8.72E-06	0.967	0.866	0.939	0.152	0.960	0.320	0.950	0.012	0.076	0.030	8.84E-04	+-+-
	18	8847726	rs662429	C	A	2.75E-06	0.644	0.079	0.655	0.948	0.625	0.347	0.592	4.02E-05	0.055	0.013	0.028	++-+
KANK2/DOCK6	19	11300365	rs138534124	C	T	2.89E-06	0.834	0.203	0.925	1.47E-04	0.927	0.339	0.964	7.86E-09	0.148	0.026	0.137	++++
NCAN	19	19336608	rs2238675	T	C	1.40E-08	0.874	2.73E-04	0.887	0.002	0.888	0.068	0.892	1.23E-13	0.164	0.022	0.399	++++
HDL																		
	2	21578468	rs219558	T	G	2.82E-06	0.730	0.891	0.655	0.803	0.636	0.884	0.538	0.004	0.039	0.014	0.003	++++
LPL	8	19891227	rs28526159	C	T	8.49E-08	0.462	0.132	0.618	0.140	0.615	0.010	0.703	8.41E-09	0.077	0.013	0.088	++++
ABCA1	9	107601541	rs2472386	G	A	5.13E-10	0.427	9.91E-04	0.436	0.005	0.460	0.829	0.488	8.37E-11	0.088	0.014	0.002	+++-
	10	107061270	rs2930450	G	T	7.75E-06	0.07	0.880	0.106	0.544	0.122	0.293	0.172	0.202	0.028	0.022	1.81E-04	+---
ZNF259/APOA5	11	116581641	rs2367970	A	G	5.82E-09	0.739	0.031	0.776	0.006	0.756	0.484	0.789	8.42E-10	0.097	0.016	0.034	++++
NOS1	12	117779856	rs56257650	C	A	4.01E-06	0.666	0.208	0.768	0.858	0.773	0.526	0.852	8.49E-05	0.061	0.016	0.051	++++
LIPC	15	58726744	rs261334	C	G	1.19E-07	0.587	0.004	0.384	1.08E-06	0.425	0.381	0.335	9.40E-13	0.094	0.013	0.019	++++
	15	60351885	rs60314318	C	T	2.35E-06	0.905	0.482	0.835	0.925	0.860	0.079	0.848	0.026	0.042	0.019	1.08E-04	++--
LOC102467079	16	52130521	rs11646391	C	G	3.57E-06	0.963	0.460	0.929	0.421	0.923	0.621	0.916	0.004	0.075	0.026	0.002	+++-
CETP	16	57006590	rs7499892	T	C	4.16E-17	0.780	2.64E-18	0.773	4.79E-17	0.754	3.78E-05	0.726	3.80E-49	0.243	0.016	8.83E-04	++++

ZNF607	19	38198540	rs62108344	T	C	7.37E-06	0.863	0.404	0.792	0.380	0.804	0.480	0.787	0.003	0.051	0.017	0.004	+++-
LDL																		
ST6GALNAC3	1	76676058	rs12064344	T	C	1.06E-06	0.054	0.310	0.107	0.804	0.082	0.348	0.093	0.094	0.040	0.024	4.13E-05	+-+-
CELSR2	1	109817838	rs660240	T	C	1.19E-12	0.822	7.27E-08	0.793	2.346E-13	0.777	0.022	0.745	1.25E-28	0.179	0.016	0.002	++++
APOB	2	21217490	rs13392272	C	T	6.01E-07	0.364	1.82E-05	0.387	0.026	0.343	0.059	0.350	2.16E-12	0.095	0.014	0.497	++++
THSD7B	2	138315429	rs140546204	G	A	7.10E-06	0.132	0.447	0.110	0.099	0.143	0.805	0.146	2.23E-04	0.073	0.020	0.019	+++-
TPRG1	3	188864748	rs76543661	T	C	5.67E-06	0.966	0.578	0.935	0.812	0.943	0.526	0.932	0.032	0.067	0.031	7.93E-04	++--
	5	3172052	rs13190086	C	T	2.53E-06	0.103	0.790	0.105	0.365	0.108	0.753	0.092	0.015	0.053	0.022	6.56E-04	++--
LINGO2	9	28018301	rs13295840	G	T	9.93E-06	0.282	0.611	0.310	0.159	0.290	0.824	0.248	7.42E-04	0.050	0.015	0.015	+++-
ITGBL1	13	102179256	rs9585723	T	G	9.44E-06	0.056	0.315	0.109	0.454	0.125	0.690	0.170	0.139	0.032	0.021	2.54E-04	+--+
ZNF771	16	30423751	rs4232893	A	G	1.24E-06	0.066	0.606	0.130	0.371	0.134	0.588	0.197	0.013	0.050	0.020	3.04E-04	++-+
DOK6	18	67284055	rs11662540	G	T	1.47E-06	0.688	0.801	0.631	0.443	0.628	0.141	0.617	0.020	0.032	0.014	1.28E-04	+-+-
NCAN	19	19336608	rs2238675	T	C	4.75E-06	0.874	0.019	0.887	0.032	0.888	0.129	0.892	2.33E-08	0.125	0.022	0.555	++++
TRIG																		
GCKR	2	27742603	rs780093	C	T	6.03E-06	0.304	9.61E-09	0.356	0.003	0.326	0.039	0.306	2.82E-15	0.108	0.014	0.320	++++
	3	165650549	rs4419424	A	G	7.36E-06	0.319	0.280	0.404	0.270	0.425	0.115	0.491	1.42E-05	0.057	0.013	0.106	++++
	4	32616342	rs13136088	A	G	8.86E-06	0.919	0.715	0.892	0.360	0.893	0.633	0.904	0.085	0.038	0.022	4.45E-04	+---
CAMK2D	4	114557476	rs2189367	G	C	8.16E-06	0.268	0.681	0.451	0.047	0.382	0.553	0.412	1.10E-04	0.052	0.013	0.024	++++
RAI14	5	34752228	rs17593349	T	C	6.62E-06	0.862	0.816	0.877	0.904	0.881	0.222	0.909	0.002	0.063	0.020	0.007	+--+
SCAMP1/AP3B1	5	77631634	rs4588572	G	A	4.80E-06	0.448	0.002	0.639	0.059	0.652	0.742	0.757	7.37E-08	0.072	0.013	0.185	++++
UIMC1	5	176395190	rs353503	G	T	8.96E-06	0.332	0.984	0.453	0.998	0.460	0.039	0.534	4.83E-04	0.046	0.013	0.008	+0-+
	7	91101465	rs4131680	G	A	5.95E-06	0.215	0.360	0.384	0.376	0.360	0.236	0.421	0.004	0.041	0.014	0.002	+-++
TRIB1	8	126491733	rs2954031	T	G	7.51E-07	0.649	4.50E-05	0.612	0.018	0.616	0.006	0.620	3.26E-13	0.098	0.013	0.791	++++
	9	18380820	rs79186818	G	A	5.93E-06	0.046	0.640	0.076	0.961	0.069	0.221	0.055	0.005	0.075	0.027	0.002	+--+
	9	26500798	rs62542129	T	C	6.63E-06	0.415	0.065	0.458	0.913	0.429	0.466	0.441	6.96E-05	0.052	0.013	0.039	++-+
ZNF259/APOA5	11	116648917	rs964184	C	G	5.32E-37	0.367	2.07E-28	0.248	4.102E-17	0.292	3.27E-07	0.216	2.79E-83	0.275	0.014	0.044	++++
	16	57387682	rs34993999	C	A	7.70E-06	0.052	0.154	0.088	0.019	0.064	0.655	0.081	6.34E-05	0.105	0.026	0.008	+++-
	18	44337708	rs3809959	G	C	6.46E-06	0.334	0.892	0.428	0.304	0.418	0.749	0.433	0.002	0.041	0.013	0.008	+-++
MAU2	19	19455750	rs8102280	A	G	9.86E-10	0.909	2.11E-07	0.956	0.005	0.953	0.017	0.954	3.38E-18	0.252	0.029	0.497	++++

Supplemental File 5. LD patterns (expressed as r2 values in the Mexican American Los Angeles reference sample and the European CEU reference sample) observed for lead SNPs observed in this study (lead SNP-Hisp), and the European GLGC meta-analysis (Lead SNP-GLGC). We also report the p-values of each lead SNP in the Hispanic and European datasets.

Gene	chr	pos	Lead SNP-Hisp	pval-Hisp	pval-GLGC	Lead SNP-GLGC	pos	pval-GLGC	pval-Hisp	Same direction of effect?	r2-MLX	r2-CEU
**TCHOL**												
CELSR2	1	109817192	rs7528419	7.88E-12	5.60E-110	rs646776	109818530	4.77E-187	3.91E-11	Yes	0.948	0.927
TRIB1	8	126474306	rs2980885	1.84E-07	8.26E-14	rs2954029	126490972	2.42E-65	1.44E-05	Yes	0.125	0.204
ZNF259	11	116648917	rs964184	3.19E-07	2.84E-55	rs964184	116648917	2.84E-55	3.19E-07	Yes	1.000	1.000
NCAN	19	19336608	rs2238675	1.40E-08	2.10E-36	rs10401969	19407718	4.13E-77	7.77E-05	Yes	0.085	0.350
**HDL**												
LPL	8	19891227	rs28526159	8.49E-08	2.51E-22	rs13702	19824492	1.28E-160	4.38E-07	Yes	0.265	0.251
ABCA1	9	107601541	rs2472386	5.13E-10	4.74E-08	rs1883025	107664301	1.50E-65	1.94E-03	Yes	0.013	0.031
ZNF259	11	116581641	rs2367970	5.82E-09	5.03E-10	rs964184	116648917	6.09E-48	1.09E-08	Yes	0.226	0.228
LIPC	15	58726744	rs261334	1.19E-07	5.73E-68	rs10468017	58678512	1.21E-188	2.13E-04	Yes	0.012	0.035
CETP	16	57006590	rs7499892	4.16E-17	1.91e-541	rs247616	56989590	1.15E-802	7.61E-11	Yes	0.042	0.104
**LDL**												
CELSR2	1	109817838	rs660240	1.19E-12	9.00E-265	rs646776	109818530	1.63E-272	8.36E-12	Yes	0.948	0.927
APOB	2	21217490	rs13392272	6.01E-07	1.46E-118	rs1367117	21263900	9.48E-183	5.57E-06	Yes	0.304	0.280
NCAN	19	19336608	rs2238675	4.75E-06	3.93E-27	rs10401969	19407718	2.65E-54	4.45E-03	Yes	0.085	0.350
**TRIG**												
GCKR	2	27742603	rs780093	6.03E-06	6.17E-220	rs1260326	27730940	2.29E-239	4.22E-05	Yes	0.908	0.901
TRIB1	8	126491733	rs2954031	7.51E-07	2.81E-109	rs2954022	126482621	2.23E-113	2.21E-06	Yes	0.877	0.966
ZNF259	11	116648917	rs964184	5.32E-37	6.55E-224	rs10790162	116639104	1.10E-249	3.80E-24	Yes	0.505	0.630
MAU2	19	19455750	rs8102280	9.86E-10	NA	rs10401969	19407718	9.70E-70	2.99E-04	Yes	0.000	NA

Supplemental File 6. P-values of markers reported in previous GWA studies for lipid traits in Mexicans (references 11 and 12) in our meta-analysis. We also report the LD patterns between the lead SNPs reported in the Mexican study and the lead SNP of our meta-analysis.

GENE	Lead SNP in Mexican GWAS	CHR	POS	Study	EA	p-val in original Mexican study	pval in our Hispanic sample	Same direction of effect?	Lead SNP in our study	pos	r2-MLX	Note
**TRIG**												
GCKR	rs1260326	2	27730940	11	T	2.20E-13	4.22E-05	Yes	rs780093	27742603	0.908	
LPL	rs12678919	8	19844222	11	T	2.70E-10	1.60E-04	Yes	NA			
LPL	rs79236614	8	19860460	12	C	3.79E-08	2.89E-04	Yes	NA			
TRIB1,LINC00861	rs4360309	8	126523523	12	T	1.60E-06	0.260	Yes	rs2954031	126491733	0.148	
ZNF259	rs964184	11	116648917	11,12	G	5.5E-35, 6.08E-33	5.32E-37	Yes	rs964184	116648917	1.000	
SIK3	rs139961185	11	116807343	12	A	1.15E-12	1.71E-08	Yes	rs964184	116648917	0.181	marker not significant when conditioning for rs964184
**HDL**												
ABCA1	rs9282541	9	107620835	11	G	6.40E-26	3.90E-05	Yes	rs2472386	107601541	0.053	
ABCA1	rs4149310	9	107589134	11	A	5.54E-08	0.898	No	rs2472386	107601541	0.652	
LIPC	rs1077835	15	58723426	11	G	2.10E-14	2.22E-07	Yes	rs261334	58726744	0.834	
CETP	rs1532624	16	57005479	11	A	1.39E-24	2.22E-12	Yes	rs7499892	57006590	0.116	
HERPUD1,CETP	rs9989419	16	56985139	12	G	2.71E-09	1.78E-08	Yes	rs7499892	57006590	0.337	
CETP	chr16:56997349:I	16	56997349	12	C	6.75E-20	5.61E-12	Yes	rs7499892	57006590	0.564	
CETP	rs5880	16	57015091	12	G	1.76E-16	5.87E-10	Yes	rs7499892	57006590	0.487	
LOC55908/DOCK6	rs2278426	19	11350488	11	C	3.44E-09	4.55E-05	Yes	NA			
**TCHOL**												
CELSR	rs3902354	1	109819296	12	A	1.16E-08	3.49E-09	Yes	rs7528419	109817192	0.600	
CETP	chr16:56997349:I	16	56997349	12	C	1.58E-08	2.84E-03	Yes	NA			
CETP	rs118146573	16	57000938	12	G	3.79E-10	2.15E-05	Yes	NA			
**Novel regions**												
**TG**												
TMEM241	rs9949617	18	20879217	11	G	2.40E-08	0.930	No	NA			
**HDL**												
UGT8,NDST4	rs78557978	4	115638601	12	G	4.09E-08	0.549	Yes	NA			
RORA	rs148533712	15	61244884	12	C	3.41E-08	0.724	Yes	NA			
